# Design, Synthesis, and Antimalarial Evaluation of Novel Quinazolin-4(3*H*)‑one Derivatives with Molecular Modeling Insights into Target Selectivity

**DOI:** 10.1021/acsomega.6c04943

**Published:** 2026-06-10

**Authors:** Igor José dos Santos Nascimento, Karla Joane da Silva Menezes, Margarida Cochicho Leonardo, Inês Morais, Carolina Silva Dias Vieira, Sara Silva Pereira, Sofia Cortes, Rui Moreira, Fátima Nogueira, Ricardo Olimpio de Moura

**Affiliations:** † Postgraduate Program of Pharmaceutical Sciences, Pharmacy Department, State University of Paraíba, Campina Grande, Paraíba 58429-500, Brazil; ‡ Global Health and Tropical Medicine (GHTM), Associate Laboratory in Translation and Innovation Towards Global Health (LA-REAL), Instituto de Higiene e Medicina Tropical (IHMT), 50106 Universidade NOVA de Lisboa (Unl), Rua da Junqueira 100, Lisbon 1349-008, Portugal; § Postgraduate Program in Development and Technological Innovation in Medicines, State University of Paraíba (UEPB), Campina Grande, Paraíba 58429-500, Brazil; ∥ Católica Biomedical Research Centre, Católica Medical School, 59207 Universidade Católica Portuguesa, Oeiras 2780-156, Portugal; ⊥ Research Institute for medicines (iMed.ULisboa),Faculty of Pharmacy, Universidade de Lisboa, Lisbon 1649-003, Portugal

## Abstract

Malaria is a tropical disease caused by protozoa of the genus Plasmodium and is responsible for several deaths worldwide. Current therapies are limited by the high incidence of adverse effects and the rising prevalence of parasite drug resistance, underscoring the need for research into new chemical scaffolds that can overcome these limitations and for the identification of novel drug targets to advance antimalarial development. In this context, quinazolines show promising potential as new antimalarials. Therefore, in this study, a new series of quinazolin-4­(3*H*)-one analogs was synthesized and evaluated for antiplasmodial activity. As a result, 36 compounds were synthesized and characterized, of which **3d**, **3e**, **4a**, and **4e** showed promising activity in assays against *P. falciparum*-3D7HT-GFP (IC_50_ = 4.36, 2.26, 1.70, and 4.10 μM) with no cytotoxicity (CC_50_ = 44.56, 22.91, 26.42, and 48.15 μM) and acceptable selectivity indexes (10.22, 10.14, 15.55, and 11.75). The compounds were evaluated against *Leishmania donovani* and *Trypanosoma congolense* but did not inhibit these parasites, suggesting that this chemical scaffold is selective for plasmodial targets. Accordingly, molecular modeling proposed *N*-myristoyltransferase (NMT) as a potential target and identified the specific binding mode of compound **4a** for *P. falciparum* NMT (PfNMT) relative to *L. donovani* NMT (LdNMT), *T. congolense* NMT (TcNMT), and *Homo sapiens* NMT (HsNMT). It was found that the presence of Leu^411^, Leu^369^, Ser^337^, and Phe^336^ in PfNMT, substituted by Met^412^, Val^370^, Val^338^, and Tyr^337^ in LdNMT, and Met^445^, Tyr^370^, Ile^371^, and Tyr^370^ in TcNMT, may be related to the predicted target selectivity and the greater affinity of **4a** for PfNMT. Finally, molecular dynamics simulations suggest that **4a** is most stable against PfNMT, and MM-PBSA calculations indicate a binding energy for this target (Δ*G_binding_* = −130.873 kJ/mol). These findings corroborate the promising potential of **4a** and support its proposed selectivity against PfNMT, yielding a scaffold that can be explored in subsequent optimization studies.

## Graphical abstract

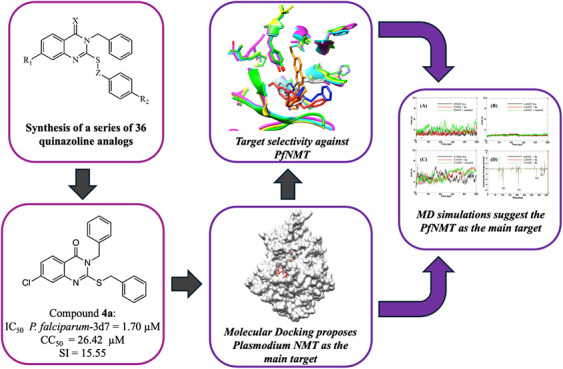

## 1 Introduction

Malaria is a life-threatening disease affecting tropical and subtropical regions worldwide.^1^ This disease is transmitted to humans by 5 species of parasites of the genus *Plasmodium*, with *P. falciparum* and *P. vivax* being the most dangerous. The first has a high prevalence in Africa, and the second is mainly found in countries outside Sub-Saharan Africa. Other species that infect humans include *P. knowlesi*, *P. ovale*, and *P. malariae*.^2^ The World Health Organization (WHO) estimates that approximately 282 million malaria cases occurred worldwide in 2024, corresponding to an incidence rate of 64.0 cases per 1,000 inhabitants at risk, up from 60.4 cases per 1,000 inhabitants in 2023.^1^ Furthermore, 50% of global malaria deaths are concentrated in four countries: Niger, the Democratic Republic of Congo, the United Republic of Tanzania, and Nigeria, with 38.6% of all global deaths in Nigeria related to children.^1–3^ This is related to the severity of the disease, which can range from mild symptoms such as fever, chills, and headache, similar to other febrile illnesses, to death if not identified and treated early.^2,4^

Antimalarial therapies are clinically classified by their chemical structure and the life-cycle stage they target. Among the most prominent are artemisinin derivatives, including Artemisinin-based combination therapies (ACTs), and quinine derivatives, including aminoquinolines.^5,6^ ACTs are usually considered first-line treatment for severe *P. falciparum* malaria due to their rapid action and high efficacy.^6^ Some options include artemether-lumefantrine (AL) as a first-line treatment in the African market and artesunate-amodiaquine as a second-line option. Other less popular alternatives include atovaquone-proguanil (Malarone), dihydroartemisinin-piperaquine (DHA-PPQ), and quinine combined with clindamycin or doxycycline.^7^ Additionally, combinations of chloroquine with tafenoquine or primaquine are used against *Plasmodium vivax*.^7^ However, such therapies are associated with side effects that can limit their use, as with quinine derivatives such as primaquine, mefloquine, amodiaquine, and chloroquine, which are frequently associated with gastrointestinal effects, dizziness, and cardiotoxicity.^6,8,9^ Furthermore, the effectiveness of these therapies has been increasingly threatened by the parasite’s growing resistance to current drugs, highlighting the need to develop new molecules that can overcome resistance mechanisms by exploring new chemical scaffolds and drug targets.^6,10–12^

Quinoline analogues (Figure 1) are frequently explored for antimalarial activity, as quinine derivatives have demonstrated this nucleus to be the main one for this purpose. It is a heterocycle with the chemical formula C_9_H_7_N, featuring a pyridine ring fused to a benzene ring.^13,14^ In this context, quinazolines and quinazolin-4­(3*H*)-one (Figure 1) share a similar structure with quinolines but differ in that a pyrimidine ring replaces the pyridine ring. They have attracted great interest in medicinal chemistry and drug development since the 19th century,^15,16^ showing vast therapeutic potential, including applications as anticancer,^17^ antiviral,^18^ antituberculosis,^19^ and anti-inflammatory^20^ agents. Importantly, these heterocycles are also being explored for their potential against malaria^21–23^ due to their structural similarity to quinolines, which provides new opportunities in drug design.^24,25^ Then, quinazoline-4­(3*H*)-one-sulfonamide^26^ and 4-quinazolin-(3*H*)-one derivatives^27^ (Figure 1) have been reported to display significant antimalarial activity against *P. falciparum* chloroquine-resistant strains, with potency in the submicromolar concentration range. These findings highlight the potential of quinazolines as promising chemical scaffolds for exploring molecular diversity, identifying novel drug targets against Plasmodium, and overcoming resistance mechanisms, thereby contributing to the development of safer and more innovative antimalarial therapies.^28–31^

**Figure 1:**
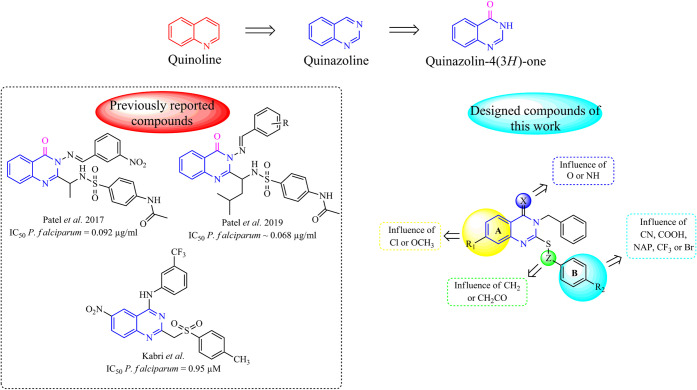
Chemical structure of the nucleus quinoline, quinazoline, and quinazolin-4­(3*H*)-one; promising analogs developed by Patel et al.,^27^ Patel et al.,^26^ and Kabri et al.^29^ The designed compounds of this work, highlighting the modifications at rings A and B, and the influence of other chemical groups.

In view of the antimalarial potential of quinazolines and their versatility in medicinal chemistry, a new series of quinazolin-4­(3*H*)-one compounds was designed to explore their structural diversity and identify new *hit* compounds for antimalarial development. Here, we report new compounds that exploit hydrogen-bond acceptor and donor groups, as well as those that contribute to lipophilicity, to identify useful structure–activity relationships (SAR) that can be applied in the development of new antimalarials (Figure 1). Furthermore, molecular modeling was used to suggest potential drug targets for this class of molecules, as well as structural features that could be related to target selectivity and contribute to antimalarial activity while being detrimental to activity against other protozoa, such as *Leishmania donovani* and *Trypanosoma congolense*, proposing selectivity against plasmodial *N*-myristoyltransferase (NMT). Our findings suggest that compound **4a** has potential predicted selectivity against *P. falciparum*. Thus, we present a compound that can be further optimized to search for novel antimalarial drugs that exploit innovative mechanisms of action.

## 2 Results and Discussions

### 2.1 Chemistry

The quinazoline analogs were synthesized following the procedure described in Figure 2. Initially, the intermediate quinazolin-4­(3*H*)-one **1** and quinazolin-4­(3*H*)-imine **2** were synthesized using benzyl isothiocyanate with ethyl 2-aminobenzoate or 2-aminobenzonitrile in ethanol under reflux, yielding the respective quinazolines. For the synthesis of the 7-substituted quinazolines **3** and **4**, the acid 2-aminobenzoic substituted with 7-Cl or 7-OCH_3_ was used with benzyl isothiocyanate in methanol and triethylamine as a base. This procedure is consistent with other reports for the synthesis of the quinazoline nuclei,^32–34^ yielding 50–75%, and was confirmed by ^1^H NMR and ^13^C NMR (see Supporting Information Figures S1–S8). Next, the intermediate products **1**, **2**, **3**, and **4** reacted with the required benzyl bromides or 2-bromoacetophenones to provide the final products **1a**–**f** to **8a**–**c**. To obtain derivatives **1a**–**f** and **2a–f,** the intermediates **1** or **2** were reacted with the corresponding benzyl bromide in EtOH/DMF using sodium acetate as a base under reflux, yielding the final products in 50–94% yield. In addition, to obtain the analogs **5a**–**c** and **6a**–**c**, an S_n_2 reaction with the corresponding 2-bromoacetophenone under similar conditions yielded the compounds in 76–99% yield. On the other hand, to provide the derivatives **3a**–**f** and **4a**–**f**, the S_n_2 reaction was performed with the intermediates **3** or **4** reacted with the required benzyl bromide in methanol/DMF using sodium acetate as a base in reflux, providing the final compounds with 76–96% yield. Finally, in a similar procedure, an S_n_2 reaction of the corresponding 2-bromoacetophenone with the intermediate compounds **3** or **4** affords the final compounds **7a**–**c** and **8a**–**c** in 69–99% yield. These procedures are consistent with other works that used a similar S_n_2 reaction with alkyl halides.^32,34^ All compounds were confirmed by ^1^H NMR (two CH_2_ singlets between 4.41 and 5.58 ppm), ^13^C NMR (two signals between 34.98 and 51.46 ppm), and mass spectra by HRMS of all compounds, which indicate that the major fragment is consistent with the proposed molecular formula of the analogs. Finally, the complete characterization of all compounds is available in the Supporting Information (see Figures S9–S74 for NMR and Figures S75–S110 for HRMS)

**Figure 2:**
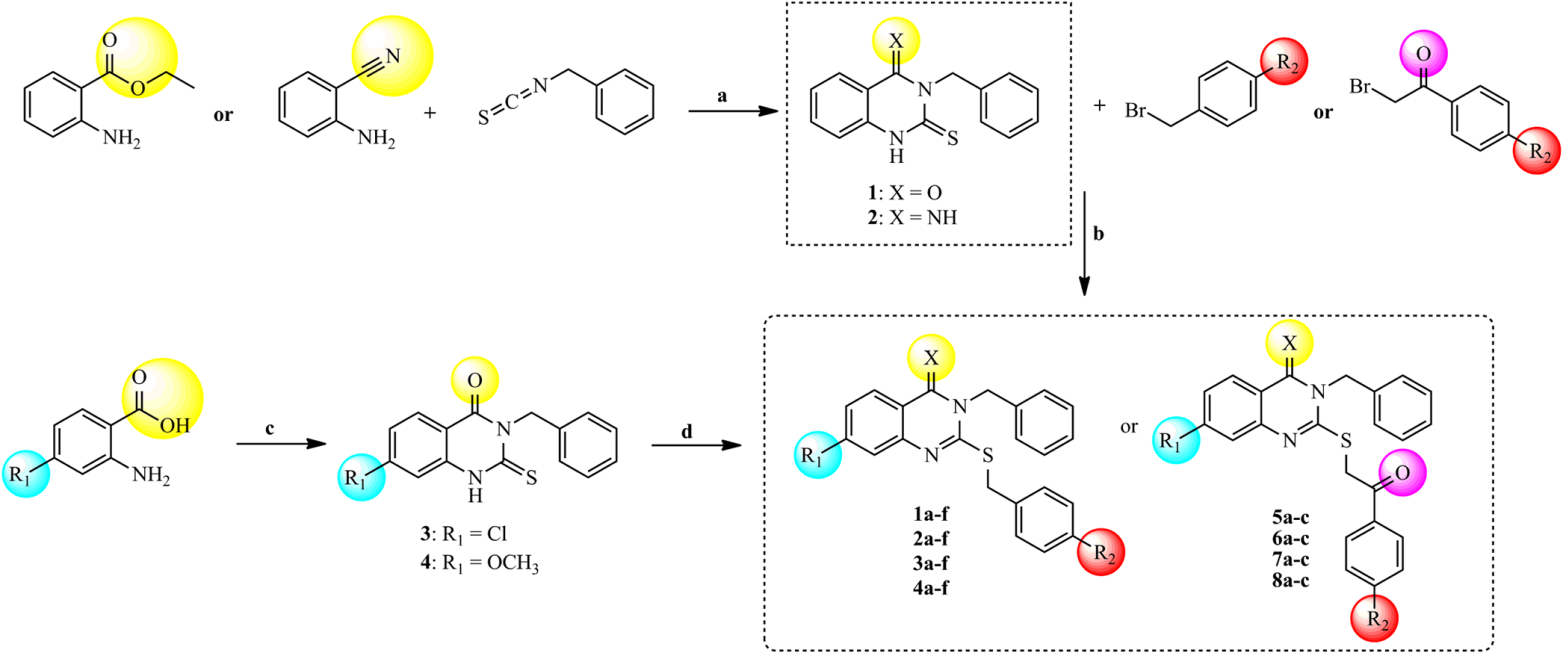
General procedure for the synthesis of compounds: a: EtOH/Reflux; b: EtOH/DMF/AcONa/Reflux; c: MeOH/N­(Et)­3/Reflux, benzyl thiocyanate; d: MeOH/DMF/AcONa/Reflux, benzyl bromides or 2-bromoacetophenones substituted.

### 2.2 Biological Evaluation

#### 2.2.1 Antimalarial Evaluation of the Initial Compounds **1a**–**f** to **2a**–**f**

The designed compounds were assayed against *the* *P. falciparum* 3D7HT-GFP strain, with chloroquine (CQ) as a positive control. The half-maximal inhibitory concentration (IC_50_) is shown in Table 1. First, compounds **1a**–**f** and **2a**–**f** were screened at a fixed concentration of 10 μM each to determine the inhibitory percentage (Ip). It was found that replacing O (**1a**–**f**) with NH (**2a**–**f**) had minimal effect on activity. For example, the Ip values for **1b** and **2b**; **1c** and **2c**; and **1d** and **2d** were similar (33 and 41%; 10% for both; 34 and 53%, respectively), indicating that the O-to-NH replacement was not relevant to activity. Furthermore, only **1e**, **2e**, and **2f** showed Ip greater than 70% and were selected for IC_50_ determination (see Supporting Information, Figure S111), yielding good results (3.95, 4.07, and 4.06 μM, respectively). Thus, a similar SAR was observed, as replacing O (**1e**) with NH (**2e**) provided similar results. On the other hand, replacing ring B with CF_3_ increased the compounds’ activity. In this way, replacing CN (**1b**), COOH (**1c**), NAP (**1d**), or Br (**1f**) with CF_3_ (**1e**) significantly improved activity. Similar results were observed in the series **2a**–**f**. In fact, the best results for **1e**, **2e,** and **2f** can be attributed to the best correlation between lipophilicity (Log P = 5.47, 5.49, and 5.15) and aqueous solubility (log S = −6.22, −6.27, and −6.34). These data suggest that increased lipophilicity with CF_3_ or Br groups is associated with improved antimalarial potential. In contrast, hydrophilic groups such as CN or COOH are detrimental to activity. These findings are consistent with other reports using a different scaffold with similar modifications.^35,36^ To confirm these findings, a new series of analogs was synthesized to improve the antimalarial potential of this scaffold.

**Table 1:**
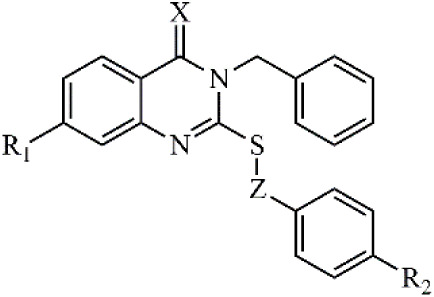
*In Vitro* Antiplasmodial Evaluation of Compounds **1a**–**f** and **8a**–**c** against *P. falciparum*-3D7HT-GFP (Ip and IC_50_) and Cell Cytotoxicity in the THP-1 Cell Line (CC_50_)

Compound	X	R_1_	Z	R_2_	Ip (%)^a^	IC_50_ (μM)^b^	CC_50_ (μM)^c^	SI^d^
**1a**	O	H	CH_2_	H	30	-	-	-
**1b**	O	H	CH_2_	CN	33	-	-	-
**1c**	O	H	CH_2_	COOH	10	-	-	-
**1d**	O	H	CH_2_	NAP	34	-	-	-
**1e**	O	H	CH_2_	CF_3_	85	3.95	31.58^e^	7.99
**1f**	O	H	CH_2_	Br	46	-	-	-
**2a**	NH	H	CH_2_	H	8	-	-	-
**2b**	NH	H	CH_2_	CN	41	-	-	-
**2c**	NH	H	CH_2_	COOH	10	-	-	-
**2d**	NH	H	CH_2_	NAP	53	-	-	-
**2e**	NH	H	CH_2_	CF_3_	72	4.07	19.27^f^	4.73
**2f**	NH	H	CH_2_	Br	70	4.06	9.30^e^	2.29
**3a**	O	Cl	CH_2_	H	82	4.07	24.40^e^	5.99
**3b**	O	Cl	CH_2_	CN	83	4.50	16.17^e^	3.59
**3c**	O	Cl	CH_2_	COOH	4	-	-	-
**3d**	O	Cl	CH_2_	NAP	83	4.36	44.56	10.22
**3e**	O	Cl	CH_2_	CF_3_	92	2.26	22.91^f^	10.14
**3f**	O	Cl	CH_2_	Br	86	5.44	20.96^e^	3.85
**4a**	O	OCH_3_	CH_2_	H	70	1.70	26.42^f^	15.55
**4b**	O	OCH_3_	CH_2_	CN	8	-	-	-
**4c**	O	OCH_3_	CH_2_	COOH	2	-	-	-
**4d**	O	OCH_3_	CH_2_	NAP	82	4.10	48.15	11.75
**4e**	O	OCH_3_	CH_2_	CF_3_	89	1.87	44.35^e^	23.71
**4f**	O	OCH_3_	CH_2_	Br	74	4.28	43.19^e^	10.10
**5a**	O	H	CH_2_CO	Cl	51	-	-	-
**5b**	O	H	CH_2_CO	CH_3_	32	-	-	-
**5c**	O	H	CH_2_CO	OCH_3_	33	-	-	-
**6a**	NH	H	CH_2_CO	Cl	65	-	-	-
**6b**	NH	H	CH_2_CO	CH_3_	50	-	-	-
**6c**	NH	H	CH_2_CO	OCH_3_	20	-	-	-
**7a**	O	Cl	CH_2_CO	Cl	61	-	-	-
**7b**	O	Cl	CH_2_CO	CH_3_	38	-	-	-
**7c**	O	Cl	CH_2_CO	OCH_3_	34	-	-	-
**8a**	O	OCH_3_	CH_2_CO	Cl	78	3.20	23.60^e^	7.38
**8b**	O	OCH_3_	CH_2_CO	CH_3_	74	2.39	16.02^f^	6.70
**8c**	O	OCH_3_	CH_2_CO	OCH_3_	77	3.78	11.47^e^	3.03
CQ	-	-	-	-	91	0.01	21.79^g^	2179

a Ip: inhibitory percentage.

b IC_50_: half-maximal inhibitory concentration.

c CC_50_: cytotoxic concentration that reduces 50% of cell growth.

d SI: selective index calculated by the ratio CC_50_/IC_50_.

e Crystals observed until 25 μM at 24 h and 48 h after incubation.

f Crystals observed until 50 μM at 24 h and 48 h after incubation.

g The CC_50_ of chloroquine (CQ) was used based on the reference by Rossi et al.^37^

#### 2.2.2 Hit-to-Lead Optimization: Modifications in Ring A (**3a**–**f** and **4a**–**f**) and Carbonyl Analogs (**5a**–**c**, **6a**–**c**, **7a**–**c**, and **8a**–**c**)

In the next step, 7-Cl and 7-OCH_3_ were introduced into ring A of the quinazolin-4­(3*H*)-one nucleus, and the Ip and IC_50_ values are shown in Table 1. All compounds showed Ip above 70% at 10 μM, except **3c**, **4b**, and **4c**, consistent with the previous finding that the hydrophilic groups CN and COOH are detrimental to antimalarial potential. Next, IC_50_ evaluation (see Supporting Information, Figures **S112** and **S113**) identified **3d**, **3e**, **4a**, **4d**, and **4e** as the most promising (IC_50_ values of 4.36, 2.26, 1.70, 4.10, and 1.87 μM, respectively), corroborating the previous assays and highlighting the importance of lipophilicity. Thus, replacing H with 7-Cl or 7-OCH_3_ in **1a**–**3a** or **4a** improved activity (IC_50_ values of 4.07 and 1.70 μM, respectively). In addition, substituting ring B of **3a** with CN (**3b**), NAP (**3d**), or Br (**3f**) yielded similar results (IC_50_ = 4.50, 4.36, and 5.44 μM), whereas COOH (**3c**) yielded an inactive analog. On the other hand, CF_3_ (**3e**) in ring B significantly increased activity (IC_50_ = 2.26 μM), corroborating previous findings. Similarly, replacing ring B of **4a** with CN (**4b**) or COOH (**4c**) yielded inactive analogs. However, substitution with NAP (**4d**) or Br (**4f**) reduced activity (IC_50_ = 4.10 and 4.28 μM, respectively), whereas CF_3_ (**4e**) showed similar activity (IC_50_ = 1.87 μM). Despite the increased lipophilicity of NAP and Br, the decrease in activity may reflect poor solubility. Next, the analogs **5a**–**c**, **6a**–**c**, **7a**–**c**, and **8a**–**c** were designed by adding the carbonyl and substituting ring B with lipophilic groups (Cl, CH_3_, and OCH_3_). After the initial screening, only **8a**–**c** showed Ip above 70% and similar IC_50_ values (3.20, 2.39, and 3.78 μM, respectively). In fact, the carbonyl group could be detrimental to antiplasmodial activity, even in the presence of lipophilic substituents such as Cl, CH_3_, or OCH_3_.

#### 2.2.3 Cytotoxicity Assay against THP-1 Reveals Compounds **3d**, **3e**, **4a**, and **4d** as the Most Promising

The cytotoxic concentration of 50% (CC_50_) of the most active compounds **1e**, **2e**, **2f**, **3a**, **3b**, **3d**, **3e**, **3f**, **4a**, **4d**, **4e**, **4f**, **8a**, **8b**, and **8c** was estimated using the human acute monocytic leukemia (THP-1) monocytic cell line, and the selectivity index (SI) was calculated as CC_50_/IC_50_ for each compound. The results are shown in Table 1 and Figures S114–S116 (see Supporting Information). The compounds had CC_50_ values ranging from 7.56 to 55.19 μM. In fact, the bromide analog **2f** and the carbonyl analogs **8a**–**c** exhibit low cellular viability (CC_50_ values of 9.30, 23.60, 16.02, and 11.47 μM, respectively), resulting in low SI (2.29, 7.38, 6.70, and 3.03, respectively). On the other hand, the nonsubstituted analog (**4a**), and those replaced by NAP (**3d** and **4d**), and CF_3_ (**3e** and **4e**), proved to be less cytotoxic (CC_50_ = 26.42, 44.56, 48.15, 22.91, and 44.35 μM) with acceptable SI > 10 (15.55, 10.22, 11.75, 10.14, and 23.71). These findings highlight the importance of CF_3_, which improves antimalarial potential without affecting cellular viability. In addition, the NAP group demonstrates its importance in increasing the analogs’ liposolubility, thereby enhancing their antimalarial potential without compromising cell viability. Curiously, replacing ring A with 7-OCH_3_ in place of 7-Cl provides compound **4a** with the best antimalarial potential (IC_50_ = 1.70 μM), with low cytotoxicity (CC_50_ = 26.42 μM) and acceptable selectivity (SI = 15.55). Finally, the next steps in this work were to evaluate the most promising compounds, **3d**, **3e**, **4a**, and **4d**, against trypanosomatids to highlight their antiprotozoal potential.

#### 2.2.4 Antitrypanosomatid Evaluation against *Leishmania donovani* and *Trypanosoma congolense* Reveals Antiplasmodial Selectivity for **3d**, **3e**, **4a**, and **4d**

To determine the antitrypanosomatid potential of the quinazoline analogs, the most promising compounds—**3d**, **3e**, **4a**, and **4d**—which were not cytotoxic, were selected for assay against *L. donovani* promastigotes. These forms were then treated for 48 h at fixed concentrations of 5, 10, and 20 μM, with amphotericin B at 2 μM serving as a positive control. The target compounds did not provide significant inhibition at these concentrations , with Ip < 50%. Compound **3e** showed the best results at 20 μM (37% inhibition), compared with **3d**, **4a**, and **4d** (3%, 19%, and 1%, respectively). By contrast, the positive control, amphotericin B, reduced promastigote growth by around 90% (Figure 3A). At the other concentrations, the results were similar. Similarly, compounds **3d**, **3e**, **4a**, and **4d** were tested against *T. congolense* by measuring parasite growth for 72 h in the presence or absence of the compounds (Figure 3B). The dynamics of hygromycin’s effect on *T. congolense* were as expected (50% killing in 24 h and 100% killing in 72 h), as were parasite doubling times across all conditions (7.7 h ± 0.58 for control, and between 7.8 ± 0.61 and 9 h ± 0.03 for experimental conditions). No significant effects on the proliferation or survival of *T. congolense* bloodstream forms were observed following treatment with any of the tested compounds (Figure 3C). Together, these data provide insight into the chemical scaffold’s antiplasmodial selectivity or target selectivity. The next step in this work was to perform molecular modeling assays to propose the biological target and the antiprotozoal selectivity of **3d**, **3e**, **4a**, and **4d.**

**Figure 3:**
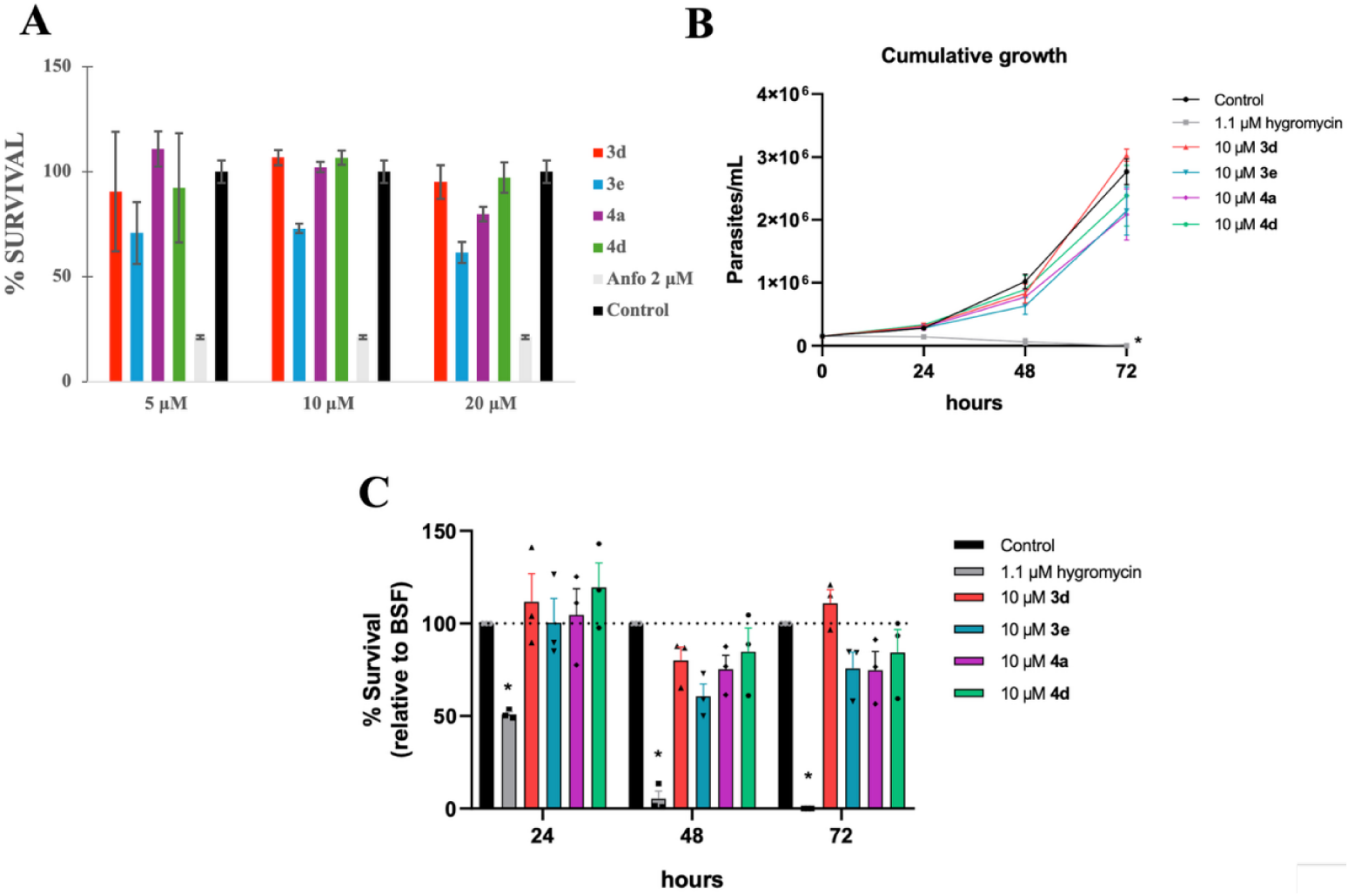
Antitrypanosomatid evaluation: (A) effects of compounds **3d**, **3e**, **4a**, and **4d** on *L. donovani*proliferation and survival at fixed concentrations of 5, 10, and 20 μM; (B) effects of compounds **3d**, **3e**, **4a**, and **4d** on *T. congolense*proliferation and survival. *T. congolense*bloodstream forms were incubated with DMSO (control) or 10 μM of the compounds for 24, 48, and 72 h, and parasite growth was quantified by cell counting using a hemocytometer; and (C) parasite survival was expressed as a percentage relative to the control parasites at each time point. The results show the mean ± standard error of the mean of three biologically independent samples. Statistical analyses were performed using two way ANOVA with Dunnett’s multiple comparisons test. *p < 0.05.

### 2.3 Molecular Modeling Studies to Propose the Biological Target

#### 2.3.1 Molecular Docking Proposes NMT as a Potential Target of the Quinazoline Analogs

The most active compounds, **3d**, **3e**, **4a**, and **4d**, were assayed against the main antiplasmodial druggable targets.^38^ In fact, these pathways have been reported as possible mechanisms of action of the quinoline analogs^13,39^ and could be promising for the quinazoline explored here. In this way, molecular docking was employed to propose the biological targets of this chemical scaffold (Table **2**). For this, the protocol was validated by analyzing the RMSD value of less than 2 Å for the cocrystallized ligand after redocking, and the fit score was used as a standard to propose the potential against the related target. Then, it was found that the best results were obtained against wild-type and quadruple mutant dihydrofolate reductase (*w*P*f*DHFR and *qm*P*f*DHFR); purine nucleoside phosphorylase (P*f*PNPase); prolyl-tRNA synthetase (P*f*ProRS); lactate dehydrogenase (P*f*LDH); Falcipain-2 (P*f*FP-2); and falcilysin (P*f*FLN) for the **3d**, **3e**, **4a**, and **4d** compared to the standard compound (Table 2). However, the plasmodial *N*-myristoyltransferase (P*v*NMT) showed the best fit score values for compounds **3d**, **3e**, **4a**, and **4d** (108.78, 103.15, 103.27, and 109.02, respectively). In fact, NMT is reported as a possible drug target with a similar scaffold.^40^ In addition, molecular docking against leishmanial NMT (L*m*NMT) (81.01, 80.78, 78.00, and 82.33, respectively) and trypanosomal NMT (T*c*NMT) (74.93, 68.20, 73.64, and 72.95, respectively) showed a decrease in the fit score of all compounds and lower results compared to the standard compounds (84.65 and 80.33, respectively). These data corroborate the biological assays and suggest the compounds’ antiplasmodial selectivity. Interestingly, the compounds showed minor interaction with human NMT (H*s*NMT) (94.91, 96.40, 85.19, and 97.30, respectively) compared to the standard compound (103.36) and to the values against plasmodial NMT, suggesting great predicted selectivity. Finally, the next stage of this work was the visual analysis of the interactions at the binding site to identify selectivity patterns.

**Table 2:** Molecular Docking Results of Compounds **3d**, **3e**, **4a**, and **4d** to Propose the Biological Target of Their Antimalarial Potential

		Fit score				
Target	RMSD (Å)^a^	Standard^b^	3d	3e	4a	4d
P*f*DHODH	0.2648	99.58	89.41	89.90	80.59	88.08
*w*P*f*DHFR	0.6635	64.67	94.49	96.48	89.49	97.72
*qm*P*f*DHFR	0.5092	62.95	93.90	89.02	84.71	95.57
P*f*PNPase	0.6658	65.97	87.52	75.05	69.36	86.27
P*f*ProRS	1.4385	70.43	88.66	86.41	88.75	88.59
P*f*LDH	0.7389	57.37	80.90	83.17	84.67	85.09
P*f*FP-2	1.84	57.12	68.58	69.92	64.01	71.55
P*f*FP-3	1.2936^c^	75.51	58.66	55.65	56.76	63.69
P*v*PMV	1.8701	113.41	89.08	75.71	66.97	77.04
P*f*PMII	1.0959	110.47	74.02	75.51	71.10	73.03
P*f*FLN	0.8890	61.09	76.59	71.90	67.87	71.59
P*f*CRT	1.519^d^	73.71	68.23	66.58	63.91	70.21
P*v*NMT	1.4088	86.66	108.78	103.15	103.27	109.02
L*m*NMT	0.3144	84.65	81.01	80.78	78.00	82.33
T*c*NMT	-	80.33^e^	74.93	68.20	73.64	72.95
H*s*NMT	0.8864	103.36	94.91	96.40	85.19	97.30

a RMSD was calculated using GOLD software by the ChemPLP score function.

b The standard compound was the cocrystallized ligand of the PDB files.

c The RMSD was calculated using the GoldScore as the score function;

d The RMSD was calculated using PyMOL software.

e The standard compound was DDD85646 available in ref. 41..

#### 2.3.2 Interactions at the Binding Site of Plasmodial NMT Propose the Key Residues Involved in the Affinity of Compounds **3d**, **3e**, **4a**, and **4d**

Analysis of compounds **3d**, **3e**, **4a**, and **4d** at the plasmodial NMT binding site (Figure 4) revealed a similar binding pose. The quinazolin nucleus provides interactions with Tyr^211^ and Tyr^334^ (π-π interactions for both) and with Asn^365^ (van der Waals), while the benzyl group provides interactions with Phe^105^ and Ser^319^ (van der Waals). In addition, the carbonyl interacts with His^213^, consistent with a previous report^42^ highlighting this residue as critical to the binding modes of potent inhibitors. Regarding the substituents, the 7-Cl (**3d** and **3e**) or 7-OCH_3_ (**4a** and **4d**) interact similarly with Leu^367^ (π-alkyl interaction). Next, the groups in ring B suggest a strong predicted binding affinity for these compounds. For example, the NAP in **3d** and **4d** interacts with Val^96^, Val^200^, and Ala^198^ by π-alkyl interaction, whereas the aromatic ring in **4a** interacts with Val^96^ by π-alkyl and with Val^200^ and Ala^198^ by van der Waals. In addition, the CF_3_ in **3e** interacts with Leu^410^ (halogen bond), Tyr^95^, and Thr^97^ (H-bond for both). Finally, these findings are corroborated by other studies^42,43^ that highlight the importance of interactions with critical residues Ser^319^, His^213^, Phe^105^, and Tyr^211^ for obtaining high-affinity inhibitors, suggesting that plasmodial NMT is a potential target for these compounds.

**Figure 4:**
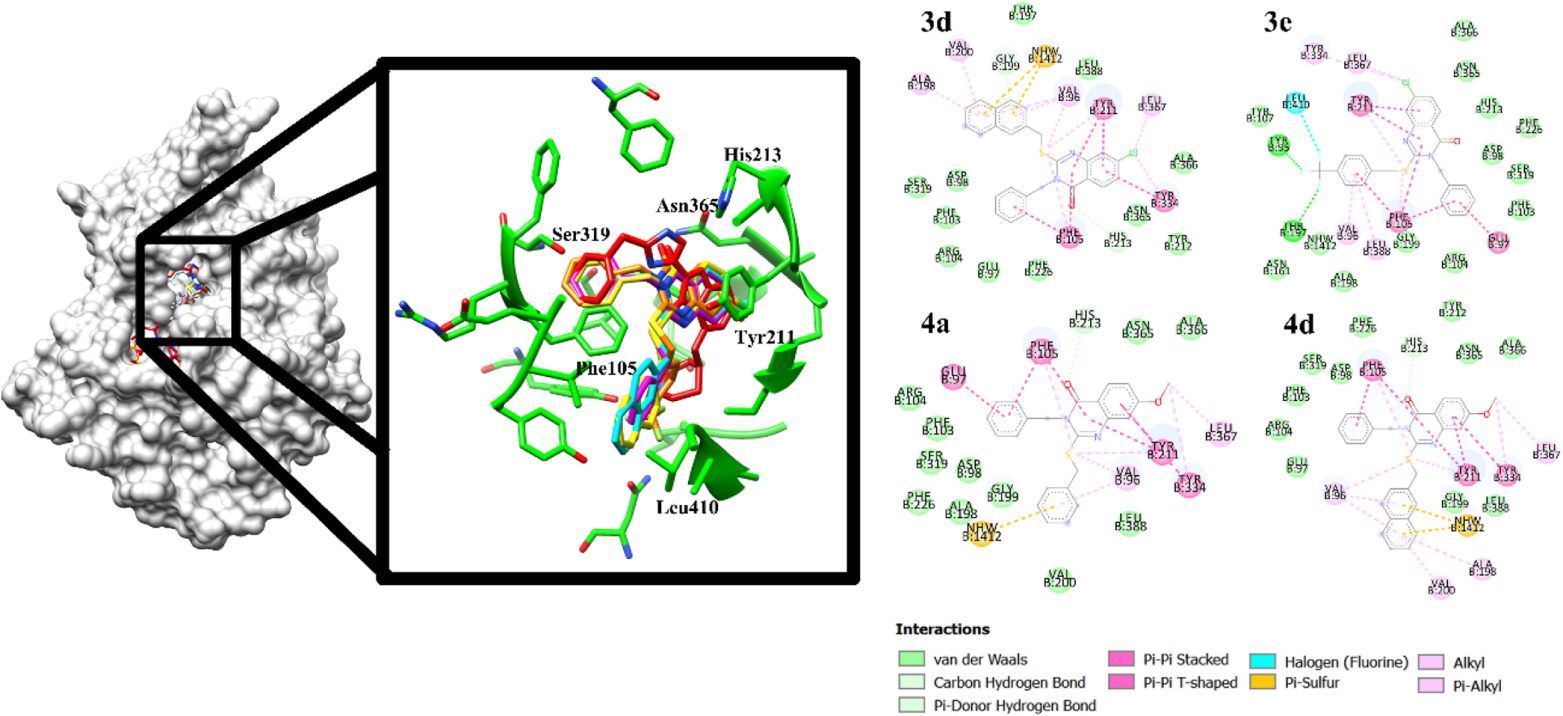
Binding modes of **3d** (cyan), **3e** (orange), **4a** (magenta), **4d** (yellow), and the standard compound (red) at the binding site of plasmodial NMT (PDB id: 4B13), highlighting the interactions of each compound and the critical residues Ser^319^, Asn^365^, His^213^, Phe^105^, Tyr^211^, and Leu^410^.

#### 2.3.3 Sequence Alignment of P*f*NMT, L*d*NMT, T*c*NMT, and H*s*NMT Suggests the Predicted Target Selectivity of the Most Active Compound **4a**

After molecular docking, sequence alignment was performed for P*f*NMT, L*d*NMT, T*c*NMT, and H*s*NMT. In addition, homology modeling was used to construct models of P*f*NMT and T*c*NMT. Thus, the binding modes of the compounds were analyzed to identify selectivity patterns of the most active compound **4a**. First, sequence alignment was performed to determine the percent identity, followed by the construction of target models by homology modeling (Figure 5A). Thus, P*f*NMT shows low identity with L*d*NMT, T*c*NMT, and H*s*NMT (40%, 35.08%, and 51.70%, respectively), suggesting the compounds have the greatest predicted selectivity potential against this drug target. In addition, similar results were observed for L*d*NMT with T*c*NMT and H*s*NMT (52.16% and 42.34%, respectively). Finally, T*c*NMT shows 40.37% identity with H*s*NMT. Differences in NMT identity can better explain the compound’s potential, and the next step was to analyze the binding modes of **4a** (Figure 5B). Further, it was found that differences in residues at the binding site modify the size and provide distinct binding modes for **4a**, suggesting predicted selectivity against *P. falciparum* (Figure 5C). For example, the replacement of Leu^411^, Leu^369^, Ser^337^, and Phe^336^ in P*f*NMT by Met^412^, Val^370^, Val^338^, and Tyr^337^ in L*d*NMT, and Met^445^, Tyr^370^, Ile^371^, and Tyr^370^ in T*c*NMT generates a more hydrophobic binding site for P*f*NMT that is capable of better accommodating **4a** and provides interactions with critical residues such as Tyr^213^ (π-π interaction with the quinazolin ring), His^215^ (carbon–hydrogen bond with quinazolin oxygen), Ser^321^, and Phe^107^ (van der Waals interactions with benzyl for both). These residues are the corresponding critical residues in P*v*NMT and have been highlighted by other reports^43,44^ as critical for enzymatic inhibition. Curiously, replacing Ser^389^, Phe^214^, Leu^412^, and Phe^336^ in P*f*NMT with Asn^473^, Trp^297^, Gly^496^, and Tyr^420^ in H*s*NMT can account for the selectivity of **4a** and the low cytotoxicity observed in biological assays. In fact, the size and the improvement in hydrophobicity of the P*f*NMT binding site favor the accommodation of **4a** and provide a robust scaffold for exploring patterns of target selectivity.

**Figure 5:**
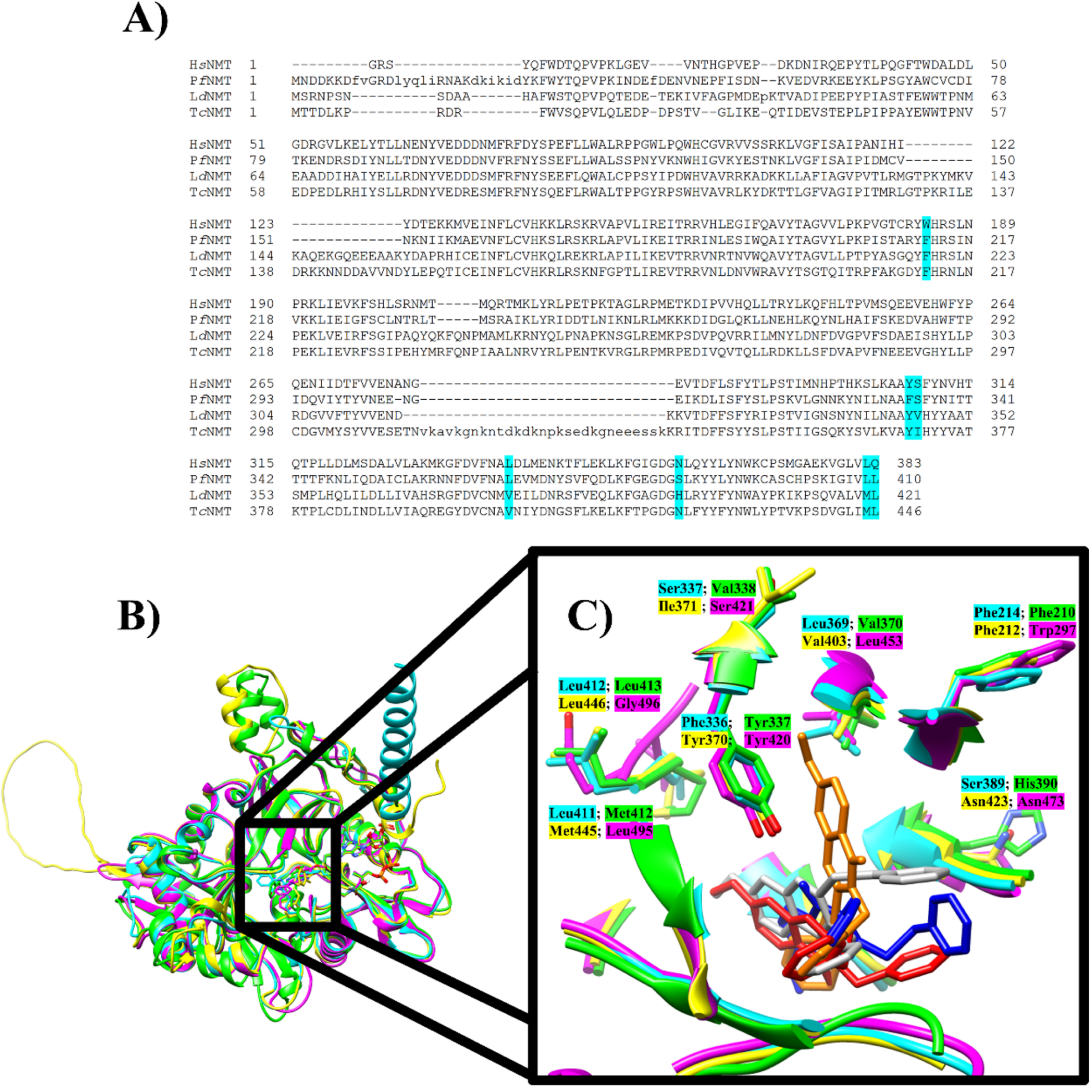
Homology modeling to provide insights about target selectivity: (A) sequence alignment of P*f*NMT, L*d*NMT, T*c*NMT, and H*s*MT highligting in cyan the different critical residues between enzymes; (B) overlay of generated models of P*f*NMT (cyan), L*d*NMT (green), T*c*NMT (yellow), and H*s*NMT (magenta) in complex with compound **4a**; (C) binding modes of **4a** in complex with P*f*NMT (orange), L*d*NMT (gray); T*c*NMT (red); and H*s*NMT (blue), highligting the critical residues involved in target selectivity.

#### 2.3.4 Molecular Dynamics Simulation Proposes the Best Stability of **4a** against Plasmodial NMT

After molecular docking, molecular dynamics (MD) simulations were performed to highlight the previous findings and to assess the stability and predicted selectivity of **4a** for P*f*NMT. Thus, the compound **4a**, complexed with P*f*NMT, L*d*NMT, and T*c*NMT, was subjected to MD simulation for 100 ns, and the results of Root Mean Square Deviation (RMSD) of C-α (Figure 6A-C) were analyzed to identify the best stability of **4a**. It is clear that the great stability of **4a** complexed with P*f*NMT (Figure 5A) provides results similar to those of free P*f*NMT and better than those of the standard compound. In addition, the RMSD values for **4a** were around 2 Å, stabilized around 50 ns, and remained consistent until the end of the simulation. These results are consistent with other studies^45^ that highlight the importance of RMSD values below 2 Å, suggesting complex stability and a possible critical interaction between the ligand and the target. In addition, **4a** complexed with L*d*NMT provides similar stability (Figure 6B), with RMSD values around 2 Å, comparable to those of free L*d*NMT and the standard compound. On the other hand, **4a,** complexed with T*c*NMT (Figure 6C), yields RMSD values above 3 Å and a less stable trajectory than free T*c*NMT, consistent with previous cellular assays.

**Figure 6:**
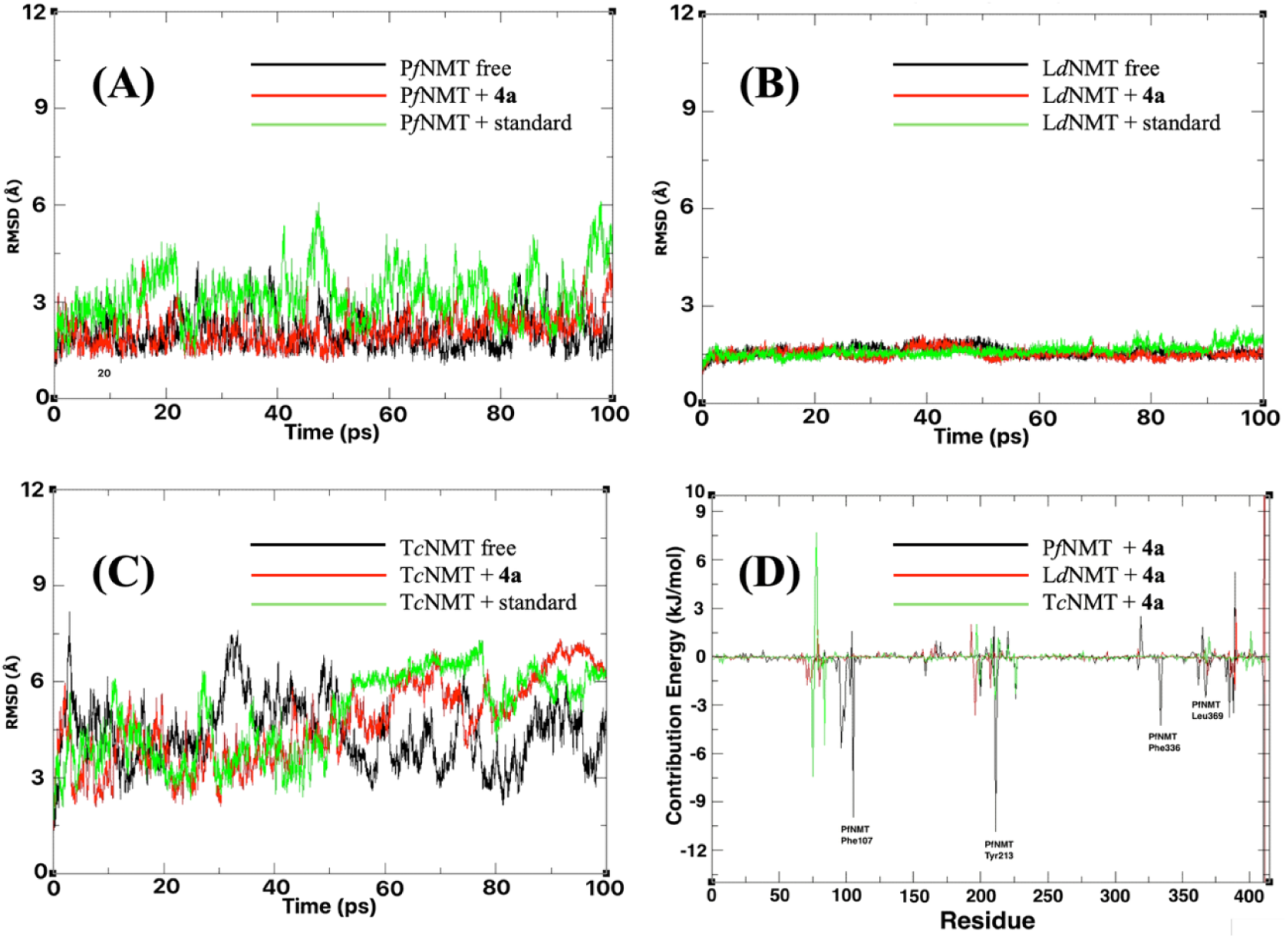
Molecular dynamics simulation results of RMSD of C-α for the: (A) P*f*NMT free (black line), and in complex with **4a** (red line) and the standard compound (green line); (B) L*d*NMT free (black line), and in complex with **4a** (red line) and the standard compound (green line); (C) T*c*NMT free (black line), and in complex with **4a** (red line) and the standard compound (green line); and (D) contribution energy per residue calculated by MM-PBSA for the **4a** complexed with P*f*NMT (black line), L*d*NMT (red line), and T*c*NMT (green line).

Furthermore, the *peer*-residue contribution energy (Figure 6D) was analyzed using the MM-PBSA method, with calculations performed from the MD simulation trajectory files. Then, similar to previous findings, the **4a** complexed with P*f*NMT provides the best contribution, and, like the previous finding of molecular docking, **4a** provides the best interaction with Phe^107^ (−9.96 kJ/mol), Tyr^213^ (−10.85 kJ/mol), Phe^336^ (−4.22 kJ/mol), and Leu^369^ (−2.5 kJ/mol), which could be related to the predicted target selectivity. In fact, **4a** complexed with L*d*NMt and T*c*NMT does not exhibit relevant interactions with critical residues compared with its complex with P*f*NMT, highlighting the potential antimalarial activity of this compound.

Finally, other MD simulation results (Figures S117–S119; see Supporting Information) provide similar insights into the antimalarial potential of **4a**. In fact, the RMSF plots (Figure S117A–C, see Supporting Information) provide insights into conformational stability, with **4a** exhibiting low fluctuations, comparable to those of standard compounds and free proteins. Further, compound **4a** shows *R*_g_ values (Figures S117D and S118A-B; see Supporting Information) similar for P*f*NMT and L*d*NMT (24 and 23 Å, respectively), indicating protein rigidity and compaction, but with values below expectations for T*c*NMT (26 Å). Furthermore, the SASA plots of the protein (Figures S118C–D and S119A, see Supporting Information) once again show that compound **4a** provides similar results for P*f*NMT (220 nm^2^) and L*d*NMT (210 nm^2^), suggesting that the solvent does not interfere with the complex stability and accessibility of the ligand at the binding site. On the other hand, T*c*NMT (250 nm^2^) shows a worse result. Finally, the SASA plots of the ligand (Figure S119B–D, see Supporting Information) provide the best values for the interaction with P*f*NMT (6.5 nm^2^) compared to L*d*NM and T*c*NMT (7.5 nm^2^ for both), indicating the permanence of the ligand in a hydrophobic environment, away from solvent molecules, and showing the best insertion of the ligand at the binding site of P*f*NMT. These findings are corroborated by experimental assays, highlighting the possible selectivity of compound **4a** against plasmodial NMT again.

#### 2.3.5 The MM-PBSA Calculations Suggest the Best Affinity of **4a** against Plasmodial NMT

After MD simulations, the trajectory files were used to calculate the binding free energy and interaction parameters for the compound **4a** complexed with P*f*NMT, L*d*NMT, and T*c*NMT using the MM-PBSA method (Table 3). In this way, the compound shows a strong predicted binding affinity for the P*f*NMT compared with L*d*NMT and T*c*NMT (Δ*G*_*binding*_ values of −130.873, −45.164, and −77.448 kJ/mol, respectively), once again consistent with previous findings of experimental assays that highlight the possible potential and selectivity. Furthermore, for all explored targets, the van der Waals forces were the most relevant in the coupling process (−185.046, −103.544, and −151.670 kJ/mol, respectively) compared with electrostatic forces (−11.441, −20.108, and −28.545 kJ/mol, respectively). In addition, the lower values of SASA and polar solvation for P*f*NMT (−21.154 and 86.768 kJ/mol, respectively) compared to L*d*NMT (−4.113 and 92.601 kJ/mol, respectively) and T*c*NMT (−19.877 and 122.643 kJ/mol, respectively) suggest a smaller solvation surface and the permanence of **4a** in the hydrophobic environment, away from water molecules, proposing its better interaction with P*f*NMT. Finally, these findings are corroborated by the experimental assays and suggest that **4a** exhibits the greatest affinity and predicted selectivity for P*f*NMT Table 3.

**Table 3:** Free Binding Energy Values and Interaction Parameters of **4a** in Complex with P*f*NMT, L*d*NMT, and T*c*NMT Calculated by MM-PBSA

	Target		
Interaction Parameter (energy)	P*f*NMT + 4a (kJ/mol)	L*d*NMT + 4a (kJ/mol)	T*c*NMT + 4a (kJ/mol)
Δ*G*_*binding*_	–130.873 ± 12.492	–45.164 ± 16.133	–77.448 ± 13.818
SASA	–21.154 ± 1.075	–4.113 ± 2.459	–19.877 ± 1.383
Polar solvation	86.768 ± 12.212	92.601 ± 22.426	122.643 ± 21.564
Electrostatic	–11.441 ± 6.813	–20.108 ± 9.143	–28.545 ± 9.019
van der Waals	–185.046 ± 12.037	–103.544 ± 17.991	–151.670 ± 12.134

## 3 Conclusion

Malaria is a tropical disease that causes significant harm to impoverished populations in many countries worldwide, generating concerns among public health agencies. Despite the vast therapeutic arsenal, the discovery of novel compounds targeting unexplored mechanisms of *Plasmodium* proliferation remains necessary to develop an innovative drug capable of overcoming resistance to current therapies. In this context, 36 new quinazoline analogs were synthesized here, of which **3d**, **3e**, **4a**, and **4e** showed promise against *P. falciparum*-3D7HT-GFP (IC_50_ = 4.36, 2.26, 1.70, and 4.10 μM) without apparent toxicity (CC_50_ = 44.56, 22.91, 26.42, and 48.15 μM) and acceptable selectivity indexes (10.22, 10.14, 15.55, and 11.75). In addition, as the first report in the literature, the compounds were evaluated against *L. donovani* and *T. congolense*. However, they were not active against them. Therefore, molecular modeling studies were performed to propose the possible target and selectivity patterns, showing for the first time in the literature the plasmodial NMT as a possible target. Interaction with residues Leu^411^, Leu^369^, Ser^337^, and Phe^336^ in P*f*NMT, substituted by Met^412^, Val^370^, Val^338^, and Tyr^337^ in L*d*NMT, and Met^445^, Tyr^370^, Ile^371^, and Tyr^370^ in T*c*NMT, may be related to the predicted target selectivity and greater affinity of **4a** for P*f*NMT. MD simulations suggest greater stability of the **4a** complexed with P*f*NMT, and MM-PBSA calculations confirm these findings and indicate selectivity for P*f*NMT (Δ*G*_*binding*_ of – 130.873 kJ/mol). Despite the great results, the next step in this work is experimental validation to confirm these findings. In addition, our experiments show that the chemical scaffold of **4a** features a quinazoline nucleus linked to 7-OCH_3_ at ring A and nonsubstituted at ring B, critical features that could be related to the antiplasmodial selectivity. Finally, we present the new compound **4a** as a promising scaffold for *hit*-to-*lead* optimization studies with the potential for antiprotozoal selectivity, generating insights that will help researchers worldwide identify an innovative compound to combat this disease, which continues to threaten the health of the global population.

## 4 Experimental Section

### 4.1 Chemicals

The starting reagents 2-aminobenzonitrile, 4-(trifluoromethyl)­benzyl bromide, benzyl bromide, 4-(bromomethyl)­benzonitrile, 2-(bromomethyl)­naphthalene, 4-(bromomethyl)­benzoic acid, benzyl isothiocyanate, ethyl 2-aminobenzoate, and others were purchased from Sigma-Aldrich (Saint Louis, MO, USA). In addition, all solvents for the analysis and synthesis were supplied by Fluka (Buchs, SG, Switzerland), Sigma-Aldrich (Saint Louis, MO, USA), and Merck (Darmstadt, Germany). The melting points were measured in capillary tubes using a Hydrosan PFM-II. Also, thin layer chromatography (TLC) was performed on Merck Analytical silica gel 60 plates, with a thickness of 0.25 mm, and visualized under UV light (254 or 365 nm). Next, the characterization was performed using proton (^1^H NMR) and carbon (^13^C NMR) nuclear magnetic resonance (NMR) on the Bruker Biospin Fourier spectrometer at 300 MHz for ^1^H and 100 MHz for ^13^C, in DMSO as the solvent. The plots were generated and interpreted using *TopSpin* software, and the coupling constants (*J*) were reported in Hertz (Hz), and the chemical shifts (δ) in parts per million (ppm). The multiplicities of the signals were designated as follows: singlet (*s*), doublet (*d*), double triplet (*dt*), triplet (*t*), and multiplet (*m*). Finally, high-resolution mass spectrometry (HRMS) was performed on an Exactive Plus Hybrid Quadrupole-Orbitrap Mass Spectrometer (Thermo Fisher Scientific) using electrospray ionization (ESI).

#### 4.1.1 General Procedure for the Synthesis of Quinazolin-4­(3*H*)-one and Quinazolin-4­(3*H*)-imine Derivatives **1**, **2**, **3**, and **4**

For the synthesis of **1** and **2**, in a round-bottom flask, 1 mmol of ethyl 2-aminobenzoate or 2-aminobenzonitrile was mixed with 1.2 mmol of benzyl isothiocyanate in 10 mL of ethanol under reflux for 12 h. For the synthesis of **3** and **4**, the procedure was similar, but with the addition of 1.2 mmol of triethylamine. The reaction was monitored by TLC, and at the end, the precipitate formed was collected, washed with ethanol and distilled water, and the final product was dried under vacuum.

##### 4.1.1.1 *3-Benzyl-2-mercaptoquinazolin-4­(3H)-one* (**1**)

White solid; Molecular Formula: C_15_H_12_N_2_OS; M.W: 268.07 g/mol; Yield: 75%; Melting Point: 240–243 °C; ^**1**^ **H NMR (DMSO-d**_**6**_ **) δ:** 5.66 (*s*, 2H, CH_2_), 7.22–7.43 (*m*, 7H, H–Ar), 7.76 (*t*, 1H, *J =* 7.68 Hz, H–Ar), 7.95 (*d*, 1H, *J =* 7.68 Hz, H–Ar), 13.09 (*s*, 1H, SH). ^**13**^ **C NMR (DMSO-d**_**6**_ **) δ:** 49.18, 115.89, 116.19, 125.10, 127.39, 127.56, 127.82, 128.69, 136.13, 137.04, 139.59, 159.87, 176.00.

##### 4.1.1.2 *3-Benzyl-4-imino-3,4-dihydroquinazoline-2-thiol* (**2**)

White solid; Molecular Formula: C_15_H_13_N_3_S; M.W: 267.08 g/mol; Yield: 70%; Melting Point: 208–211 °C; ^**1**^ **H NMR (DMSO-d**_**6**_ **) δ:** 5.84 (*s*, 2H, CH_2_), 7.21–7.31 (*m*, 7H, H–Ar), 7.56 (*t*, 1H, *J =* 7.75 Hz, H–Ar), 8.09 (*d*, 1H, *J =* 7.75 Hz, H–Ar), 9.45 (*s*, 1H, NH), 12.28 (*s*, 1H, SH). ^**13**^ **C NMR (DMSO-d**_**6**_ **) δ:** 50.12, 115.04, 116.19, 124.59, 126.61, 126.88, 127.33, 128.46, 133.73, 135.95, 137.83, 153.92, 175.48.

##### 4.1.1.3 *3-Benzyl-7-chloro-2-thioxo-2,3-dihydroquinazolin-4­(1H)-one* (**3**)

White solid; Yield: 77%; Melting Point: 241–243 °C. ^**1**^ **H NMR (DMSO-d**_**6**_ **) δ:** 5.62 (*s*, 2H, CH_2_), 7.20 – 7.41 (*m*, 7H, H–Ar), 7.93 (*d*, 1H, *J =* 8.47 Hz, H–Ar), 13.11 (*s*, 1H, NH). ^**13**^ **C NMR (DMSO-d**_**6**_ **) δ:** 49.24, 114.84, 115.51, 125.16, 127.44, 127.57, 128.69, 130.03, 136.79, 140.46, 159.27, 176.43.

##### 4.1.1.4 *3-Benzyl-7-methoxy-2-thioxo-2,3-dihydroquinazolin-4­(1H)-one* (**4**)

White solid; Yield: 50%; Melting Point: 257–261 °C. ^**1**^ **H NMR (DMSO-d**_**6**_ **) δ:** 3.84 (*s*, 3H, CH_3_), 5.63 (*s*, 2H, CH_2_), 6.88–6.94 (*m*, 2H, H–Ar), 7.23–7.29 (*m*, 5H, H–Ar), 7.86 (*d*, 1H, *J =* 8.82 Hz, H–Ar), 12.92 (*s*, 1H, NH). ^**13**^ **C NMR (DMSO-d**_**6**_ **) δ:** 48.98, 56.29, 98.81, 109.29, 113.61, 127.36, 127.54, 128.68, 129.87, 137.17, 141.43, 159.37, 165.19, 176.25.

#### 4.1.2 General Procedure for the Synthesis of Quinazolinone and Quinazoloimine Derivatives **1a**–**f** to **8a**–**c**

For the synthesis of **1a**–**f** to **2a**–**f**, 1 mmol of compound **1** or **2**, 1.2 mmol of the required substituted benzyl bromide, and 2 mmol of sodium acetate were added to a round-bottom flask with 10 mL of ethanol and 5 mL of DMF, and the mixture was refluxed for 3 h. For compounds **3a**–**f**, **4a**–**f**, **6a**–**c**, **7a**–**c**, and **8a**–**c**, the reaction was carried out in 10 mL of methanol with 4 mL of DMF using a similar protocol. The reaction was monitored by TLC, and distilled water was added to the reaction mixture at the end. The precipitate formed was washed with ethanol and distilled water, and the resulting solid was dried under vacuum.

##### 4.1.2.1 *3-Benzyl-2-(benzylthio)­quinazolin-4­(3H)-one* (**1a**)

White solid; Yield: 51%; Melting Point: 108–109 °C. ^**1**^ **H NMR (DMSO-d**_**6**_ **) δ:** 4.52 (*s*, 2H, CH_2_), 5.30 (*s*, 2H, CH_2_), 7.20–7.33 (*m*, 8H, H–Ar), 7.45–7.51 (*m*, 3H, H–Ar), 7.66 (*d*, 1H, *J =* 8.08 Hz, H–Ar), 7.83 (*t*, 1H, *J =* 7.16 Hz, H–Ar), 8.11 (*d*, 1H, *J =* 7.63 Hz, H–Ar). ^**13**^ **C NMR (DMSO-d**_**6**_ **) δ:** 36.11, 47.22, 116.19, 119.22, 126.51, 126.62, 127.13, 127.57, 127.87, 128.69, 128.90, 129.04, 129.83, 135.46, 136.10, 137.10, 147.28, 156.93, 161.39. **HRMS (ESI** ^**+**^ **) [M + H]** ^**+**^: calcd for C_22_H_18_N_2_OS: 359.1213, found: 359.1211.

##### 4.1.2.2 *4-(((3-Benzyl-4-oxo-3,4-dihydroquinazolin-2-yl)­thio)­methyl)­benzonitrile* (**1b**)

White solid; Yield: 50%; Melting Point: 129–131 °C. ^**1**^ **H NMR (DMSO-d**_**6**_ **) δ:** 4.58 (*s*, 2H, CH_2_), 5.30 (*s*, 2H, CH_2_), 7.19–7.33 (*m*, 5H, H–Ar), 7.49 (*t*, 1H, *J =* 7.40 Hz, H–Ar), 7.65–7.69 (*m*, 3H, H–Ar), 7.75 – 7.86 (*m*, 3H, H–Ar), 8.10 (*d*, 1H, *J =* 7.40 Hz, H–Ar). ^**13**^ **C NMR (DMSO-d**_**6**_ **) δ:** 35.37, 47.24, 110.42, 119.20, 126.48, 126.75, 127.09, 127.89, 129.06, 130.77, 132.68, 135.49, 136.01, 143.83, 147.14, 156.42, 161.36, 162.08. **HRMS (ESI** ^**+**^ **) [M + H]** ^**+**^: calcd for C_23_H_17_N_3_OS: 384.1165, found: 384.1160.

##### 4.1.2.3 *4-(((3-Benzyl-4-oxo-3,4-dihydroquinazolin-2-yl)­thio)­methyl)­benzoic acid* (**1c**)

White solid; Yield: 70%; Melting Point: 192–195 °C. ^**1**^ **H NMR (DMSO-d**_**6**_ **) δ:** 4.57 (*s*, 2H, CH_2_), 5.30 (*s*, 2H, CH_2_), 7.19–7.30 (*m*, 5H, H–Ar), 7.45–7.67 (*m*, 4H, H–Ar), 7.81–7.86 (*m*, 3H, H–Ar), 8.10 (*d*, 1H, *J =* 7.58 Hz, H–Ar), 12.95 (*s*, 1H, OH). ^**13**^ **C NMR (DMSO-d**_**6**_ **) δ:** 35.58, 47.23, 119.22, 126.50, 126.68, 127.11, 127.87, 129.05, 129.80, 129.97, 130.18, 135.47, 136.06, 142.68, 147.20, 156.60, 161.37, 167.49. **HRMS (ESI** ^**+**^ **) [M + H]** ^**+**^: calcd for C_23_H_18_N_2_O_3_S: 403.1111, found: 403.1115.

##### 4.1.2.4 *3-Benzyl-2-((naphthalen-2-ylmethyl)­thio)­quinazolin-4­(3H)-one* (**1d**)

White solid; Yield: 83%; Melting Point: 185 °C. ^**1**^ **H NMR (DMSO-d**_**6**_ **) δ:** 4.68 (*s*, 2H, CH_2_), 5.30 (*s*, 2H, CH_2_), 7.19–7.29 (*m*, 5H, H–Ar), 7.46–7.50 (*m*, 3H, H–Ar), 7.58 (*d*, 1H, *J =* 8.31 Hz, H–Ar), 7.72 (*d*, 1H, *J =* 8.06 Hz, H–Ar), 7.83–7.86 (*m*, 3H, H–Ar), 8.08 (*s*, 1H, H–Ar), 8.10 (*d,* 1H, *J =* 7.44 Hz, H–Ar). ^**13**^ **C NMR (DMSO-d**_**6**_ **) δ:** 36.37, 47.21, 116.18, 119.23, 125.09, 126.51, 126.78, 127.13, 127.56, 127.84, 127.99, 128.47, 128.57, 128.69, 129.02, 132.62, 133.17, 134.64, 135.46, 137.05, 147.29. **HRMS (ESI** ^**+**^ **) [M + H]** ^**+**^: calcd for C_26_H_20_N_2_OS: 409.1369, found: 409.1370.

##### 4.1.2.5 3*-Benzyl-2-((4-(trifluoromethyl)­benzyl)­thio)­quinazolin-4­(3H)-one* (**1e**)

White solid; Yield: 78%; Melting Point: 101–102 °C. ^**1**^ **H NMR (DMSO-d**_**6**_ **) δ:** 4.59 (*s*, 2H, CH_2_), 5.30 (*s*, 2H, CH_2_), 7.19–7.30 (*m*, 5H, H–Ar), 7.48 (*t*, 1H, *J =* 7.36 Hz, H–Ar), 7.63–7.71 (*m*, 5H, H–Ar), 7.83 (*t*, 1H, *J =* 7.03 Hz, H–Ar), 8.10 (*d*, 1H, *J =* 7.52 Hz, H–Ar). ^**13**^ **C NMR (DMSO-d**_**6**_ **) δ:** 35.67, 47.66, 119.66, 126.04, 126.09, 126.91, 127.13, 127.53, 128.29, 128.48, 128.90, 129.46, 131.01, 135.89, 136.46, 143.14, 147.60, 156.96, 161.79. **HRMS (ESI** ^**+**^ **) [M + H]** ^**+**^: calcd for C_23_H_17_F_3_N_2_OS: 427.1086, found: 427.1082.

##### 4.1.2.6 *3-Benzyl-2-((4-bromobenzyl)­thio)­quinazolin-4­(3H)-one* (**1f**)

White solid; Yield: 94%; Melting Point: 113 °C. ^**1**^ **H NMR (DMSO-d**_**6**_ **) δ:** 4.48 (*s*, 2H, CH_2_), 5.29 (*s*, 2H, CH_2_), 7.18–7.32 (*m*, 5H, H–Ar), 7.41–7.49 (*m*, 5H, H–Ar), 7.65 (*d*, 1H, *J =* 8.05 Hz, H–Ar), 7.83 (*t*, 1H, *J =* 7.54 Hz, H–Ar), 8.10 (*d*, 1H, *J =* 7.82 Hz, H–Ar). ^**13**^ **C NMR (DMSO-d**_**6**_ **) δ:** 35.24, 47.22, 119.22, 120.95, 126.49, 126.67, 127.12, 127.87, 129.04, 131.70, 132.02, 135.46, 136.06, 137.03, 147.21, 156.68, 161.36. **HRMS (ESI** ^**+**^ **) [M + H]** ^**+**^: calcd for C_22_H_18_BrN_3_S: 439.0303, found: 439.0297.

##### 4.1.2.7 *3-Benzyl-2-(benzylthio)­quinazolin-4­(3H)-imine* (**2a**)

White solid; Yield: 50%; Melting Point: 209–21 °C. ^**1**^ **H NMR (DMSO-d**_**6**_ **) δ:** 4.57 (*s*, 2H, CH_2_), 5.58 (*s*, 2H, CH_2_), 7.24–7.37 (*m*, 8H, H–Ar), 7.47 (*d*, 2H, *J =* 6.91 Hz, H–Ar), 7.73 (*t*, 1H, *J =* 7.76 Hz, H–Ar), 7.89 (*d*, 1H, *J =* 8.05 Hz, H–Ar), 8.08 (*t*, 1H, *J =* 7.76 Hz, H–Ar), 8.59 (*d*, 1H, *J =* 8.19 Hz, H–Ar), 10.43 (*s*, 1H, NH). ^**13**^ **C NMR (DMSO-d**_**6**_ **) δ:** 36.79, 51.46, 113.09, 125.93, 126.46, 127.51, 128.08, 128.33, 128.43, 128.93, 129.34, 129.96, 137.79, 145.63, 155.87, 157.65. **HRMS (ESI** ^**+**^ **) [M + H]** ^**+**^: calcd for C_22_H_19_N_3_S: 358.1372, found: 358.1368.

##### 4.1.2.8 *4-(((3-Benzyl-4-imino-3,4-dihydroquinazolin-2-yl)­thio)­methyl)­benzonitrile* (**2b**)

White solid; Yield: 65%; Melting Point: 190–195 °C. ^**1**^ **H NMR (DMSO-d**_**6**_ **) δ:** 4.62 (*s*, 2H, CH_2_), 5.56 (*s*, 2H, CH_2_), 7.23–7.37 (*m*, 5H, H–Ar), 7.67–7.84 (*m*, 6H, H–Ar), 8.02 (*t*, 1H, *J =* 7.62 Hz, H–Ar), 8.54 (*d*, 1H, *J =* 8.14 Hz, H–Ar), 10.10 (*s*, 1H, NH). ^**13**^ **C NMR (DMSO-d**_**6**_ **) δ:** 35.91, 51.06, 110.55, 113.89, 119.16, 125.99, 126.51, 127.34, 128.11, 128.32, 129.28, 130.90, 132.68, 133.05, 137.21, 143.32, 145.30, 155.57, 157.43. **HRMS (ESI** ^**+**^ **) [M + H]** ^**+**^: calcd for C_23_H_18_N_4_S: 383.1325, found: 383.1318.

##### 4.1.2.9 *4-(((3-Benzyl-4-imino-3,4-dihydroquinazolin-2-yl)­thio)­methyl)­benzoic acid* (**2c**)

White solid; Yield: 82%; Melting Point: 160–163 °C. ^**1**^ **H NMR (DMSO-d**_**6**_ **) δ:** 4.60 (*s*, 2H, CH_2_), 5.54 (*s*, 2H, CH_2_), 7.24 (*d*, 2H, *J =* 6.68 Hz, H–Ar), 7.33 (*t*, 3H, *J =* 7.30 Hz, H–Ar), 7.59 (*d*, 2H, *J =* 8.12 Hz, H–Ar), 7.66 (*t*, 1H, *J =* 7.61 Hz, H–Ar), 7.80–7.87 (*m*, 3H, H–Ar), 7.99 (*t*, 1H, *J =* 7.61 Hz, H–Ar), 8.49 (*d*, 1H, *J =* 8.12 Hz, H–Ar). ^**13**^ **C NMR (DMSO-d**_**6**_ **) δ:** 36.08, 50.83, 114.15, 125.86, 126.53, 127.33, 127.95, 128.26, 129.26, 129.78, 130.09, 130.26, 133.30, 136.97, 142.25, 145.27, 155.75, 157.34, 167.46. **HRMS (ESI** ^**+**^ **) [M + H]** ^**+**^: calcd for C_23_H_19_N_3_O_2_S: 402.1271, found: 402.1264.

##### 4.1.2.10 *3-Benzyl-2-((naphthalen-2-ylmethyl)­thio)­quinazolin-4­(3H)-imine* (**2d**)

White solid; Yield: 85%; Melting Point: 188–191 °C. ^**1**^ **H NMR (DMSO-d**_**6**_ **) δ:** 4.68 (*s*, 2H, CH_2_), 5.50 (*s*, 2H, CH_2_), 7.22 (*d*, 2H, *J =* 7.55 Hz, H–Ar), 7.29 (*t*, 2H, *J =* 7.30 Hz, H–Ar), 7.46–7.49 (*m*, 2H, H–Ar), 7.53 – 7.58 (*m*, 2H, H–Ar), 7.77 (*d*, 1H, *J =* 8.05 Hz, H–Ar), 7.83 – 7.88 (*m*, 4H, H–Ar), 8.01 (*s*, 1H, H–Ar), 8.38 (*d*, 1H, *J =* 7.99 Hz, H–Ar), 9.74 (*s*, 1H, NH). ^**13**^ **C NMR (DMSO-d**_**6**_ **) δ:** 36.62, 49.83, 115.62, 125.85, 126.57, 126.63, 126.81, 127.08, 127.28, 127.91, 127.98, 128.01, 128.45, 129.10, 132.63, 133.14, 134.48, 135.78, 144.95, 156.18, 156.84. **HRMS (ESI** ^**+**^ **) [M + H]** ^**+**^: calcd for C_26_H_21_N_3_S: 408.1529, found: 408.1523.

##### 4.1.2.11 *3-Benzyl-2-((4-(trifluoromethyl)­benzyl)­thio)­quinazolin-4­(3H)-imine* (**2e**)

White solid; Yield: 90%; Melting Point: 216–219 °C. ^**1**^ **H NMR (DMSO-d**_**6**_ **) δ:** 4.63 (*s*, 2H, CH_2_), 5.56 (*s*, 2H, CH_2_), 7.24–7.37 (*m*, 5H, H–Ar), 7.65–7.74 (*m*, 5H, H–Ar), 7.87 (*d*, 2H, *J =* 8.01 Hz, H–Ar), 8.04 (*t*, 1H, *J =* 7.69 Hz, H–Ar), 8.55 (*d*, 1H, *J =* 8.20 Hz, H–Ar), 10.10 (*s*, 1H, NH). ^**13**^ **C NMR (DMSO-d**_**6**_ **) δ:** 35.83, 51.23, 113.65, 125.61, 125.66, 125.90, 126.49, 127.41, 128.21, 128.37, 128.60, 129.30, 130.76, 132.87, 137.41, 142.19, 145.41, 155.61, 157.56. **HRMS (ESI** ^**+**^ **) [M + H]** ^**+**^: calcd for C_23_H_18_F_3_N_3_S: 426.1246, found: 426.1237.

##### 4.1.2.12 *3-Benzyl-2-((4-bromobenzyl)­thio)­quinazolin-4­(3H)-imine* (**2f**)

White solid; Yield: 99%; Melting Point: 149–151 °C. ^**1**^ **H NMR (DMSO-d**_**6**_ **) δ:** 4.41 (*s*, 2H, CH_2_), 5.39 (*s*, 2H, CH_2_), 7.18–7.48 (*m*, 11H, H–Ar), 7.61 (*t*, 1H, *J =* 7.41 Hz, H–Ar), 8.17 (*d*, 1H, *J =* 7.92 Hz, H–Ar), 8.83 (*s*, 1H, NH). ^**13**^ **C NMR (DMSO-d**_**6**_ **) δ:** 34.98, 47.76, 118.70, 120.79, 125.82, 126.00, 126.51, 126.81, 127.40, 128.83, 131.65, 131.95, 133.25, 136.82, 137.45, 143.98, 156.58. **HRMS (ESI** ^**+**^ **) [M + H]** ^**+**^: calcd for C_22_H_19_BrN_3_S: 438.0463, found: 438.0455.

##### 4.1.2.13 *3-Benzyl-2-(benzylthio)-7-chloroquinazolin-4­(3H)-one* (**3a**)

White solid; Yield: 76%; Melting Point: 143–144 °C. ^**1**^ **H NMR (DMSO-d**_**6**_ **) δ:** 4.51 (*s*, 2H, CH_2_), 5.27 (*s*, 2H, CH_2_), 7.19–7.32 (*m*, 8H, H–Ar), 7.44–7.52 (*m*, 3H, H–Ar), 7.73 (*d*, 1H, *J =* 1.69 Hz, H–Ar), 8.09 (*d*, 1H, *J =* 8.57 Hz, H–Ar). ^**13**^ **C NMR (DMSO-d**_**6**_ **) δ:** 36.13, 47.43, 118.05, 125.63, 126.84, 127.13, 127.91, 128.93, 129.06, 129.24, 129.85, 135.81, 137.02, 140.02, 148.20, 158.86, 160.83. **HRMS (ESI** ^**+**^ **) [M + H]** ^**+**^: calcd for C_22_H_18_ClN_2_OS: 393.0823, found: 393.0821.

##### 4.1.2.14 *4-(((3-Benzyl-7-chloro-4-oxo-3,4-dihydroquinazolin-2-yl)­thio)­methyl)­benzonitrile* (**3b**)

White solid; Yield: 86%; Melting Point: 145 °C. ^**1**^ **H NMR (DMSO-d**_**6**_ **) δ:** 4.57 (*s*, 2H, CH_2_), 5.27 (*s*, 2H, CH_2_), 7.19–7.33 (*m*, 5H, H–Ar), 7.50 (*d*, 1H, *J =* 8.60 Hz, H–Ar), 7.72 (*ddd*, 5H, *J =* 8.41 and 16.24 Hz, H–Ar), 8.08 (*d*, 1H, *J =* 8.41 Hz, H–Ar). ^**13**^ **C NMR (DMSO-d**_**6**_ **) δ:** 35.38, 47.36, 110.45, 118.05, 119.20, 125.63, 126.93, 127.11, 127.95, 129.07, 129.21, 130.83, 132.70, 140.04, 143.74, 148.05, 158.35, 160.78. **HRMS (ESI** ^**+**^ **) [M + H]** ^**+**^: calcd for C_23_H_17_ClN_3_OS: 418.0775, found: 418.0779.

##### 4.1.2.15 *4-(((3-Benzyl-7-chloro-4-oxo-3,4-dihydroquinazolin-2-yl)­thio)­methyl)­benzoic acid* (**3c**)

White solid; Yield: 86%; Melting Point: 187–191 °C. ^**1**^ **H NMR (DMSO-d**_**6**_ **) δ:** 4.57 (*s*, 2H, CH_2_), 5.28 (*s*, 2H, CH_2_), 7.19–7.33 (*m*, 6H, H–Ar), 7.54 (*dd*, 3H, *J =* 8.03 and 8.86 Hz, H–Ar), 7.86 (*d*, 2H, *J =* 8.03 Hz, H–Ar), 8.08 (*d*, 1H, *J =* 8.50 Hz, H–Ar). ^**13**^ **C NMR (DMSO-d**_**6**_ **) δ:** 35.06, 47.35, 118.04, 125.64, 126.88, 127.13, 127.93, 129.06, 129.20, 129.83, 130.01, 130.17, 135.78, 140.03, 142.60, 148.11, 158.52, 160.80, 167.50. **HRMS (ESI** ^**+**^ **) [M + H]** ^**+**^: calcd for C_23_H_18_ClN_2_O_3_S: 437.0721, found: 437.0723.

##### 4.1.2.16 *3-Benzyl-7-chloro-2-((naphthalen-2-ylmethyl)­thio)­quinazolin-4­(3H)-one* (**3d**)

White solid; Yield: 95%; Melting Point: 141–143 °C. ^**1**^ **H NMR (DMSO-d**_**6**_ **) δ:** 4.68 (*s*, 2H, CH_2_), 5.28 (*s*, 2H, CH_2_), 7.19–7.29 (*m*, 5H, H–Ar), 7.47–7.52 (*m*, 3H, H–Ar), 7.58 (*d*, 1H, *J =* 8.32 Hz, H–Ar), 7.81 – 7.87 (*m*, 4H, H–Ar), 8.01 (*s*, 1H, H–Ar), 8.09 (*d*, 1H, *J =* 8.61 Hz, H–Ar). ^**13**^ **C NMR (DMSO-d**_**6**_ **) δ:** 36.40, 47.34, 118.06, 125.66, 126.55, 126.80, 126.84, 127.14, 127.90, 127.99, 128.02, 128.49, 128.58, 129.05, 129.22, 132.63, 133.19, 134.55, 135.82, 140.04, 148.21, 158.76, 160.82. **HRMS (ESI** ^**+**^ **) [M + H]** ^**+**^: calcd for C_26_H_20_ClN_2_OS: 443.0979, found: 443.0977.

##### 4.1.2.17 *3-Benzyl-7-chloro-2-((4-(trifluoromethyl)­benzyl)­thio)­quinazolin-4­(3H)-one* (**3e**)

White solid; Yield: 95%; Melting Point: 112–114 °C. ^**1**^ **H NMR (DMSO-d**_**6**_ **) δ:** 4.59 (*s*, 2H, CH_2_), 5.28 (*s*, 2H, CH_2_), 7.19–7.33 (*m*, 5H, H–Ar), 7.50 (*dd*, 1H, *J =* 8.43 and 1.72 Hz, H–Ar), 7.68 (*q*, 4H, *J =* 8.30, and 8.18 Hz, H–Ar), 7.75 (*d*, 1H, *J =* 1.60 Hz, H–Ar), 8.08 (*d*, 1H, *J =* 8.55 Hz, H–Ar). ^**13**^ **C NMR (DMSO-d**_**6**_ **) δ:** 35.26, 47.36, 118.06, 125.64, 125.69, 126.46, 126.91, 127.12, 127.94, 128.50, 129.05, 129.21, 130.64, 135.76, 140.04, 142.64, 148.10, 158.46, 160.80. **HRMS (ESI** ^**+**^ **) [M + H]** ^**+**^: calcd for C_23_H_17_ClF_3_N_2_OS: 461.0697, found: 461.0695.

##### 4.1.2.18 *3-Benzyl-2-((4-bromobenzyl)­thio)-7-chloroquinazolin-4­(3H)-one* (**3f**)

White solid; Yield: 85%; Melting Point: 130–132 °C. ^**1**^ **H NMR (DMSO-d**_**6**_ **) δ:** 4.48 (*s*, 2H, CH_2_), 5.27 (*s*, 2H, CH_2_), 7.19–7.31 (*m*, 5H, H–Ar), 7.42–7.53 (*m*, 5H, H–Ar), 7.75 (*d*, 1H, *J =* 1.55 Hz, H–Ar), 8.09 (*s*, 1H, *J =* 8.59 Hz, H–Ar). ^**13**^ **C NMR (DMSO-d**_**6**_ **) δ:** 35.26, 47.34, 118.05, 120.98, 125.64, 126.88, 127.12, 127.93, 129.06, 129.22, 131.72, 132.07, 135.78, 136.98, 140.03, 148.13, 158.61, 160.80. **HRMS (ESI** ^**+**^ **) [M + H]** ^**+**^: calcd for C_22_H_17_BrClN_2_OS: 472.9913, found: 472.9908.

##### 4.1.2.19 *3-Benzyl-2-(benzylthio)-7-methoxyquinazolin-4­(3H)-one* (**4a**)

White solid; Yield: 95%; Melting Point: 156–157 °C. ^**1**^ **H NMR (DMSO-d**_**6**_ **) δ:** 3.93 (*s*, 3H, OCH_3_), 4.53 (*s*, 2H, CH_2_), 5.28 (*s*, 2H, CH_2_), 7.05–7.08 (*m*, 2H, H–Ar), 7.18–7.33 (*m*, 8H, H–Ar), 7.45 (*d*, 1H, *J =* 7.05 Hz, H–Ar), 8.01 (*d*, 1H, *J =* 8.54 Hz, H–Ar). ^**13**^ **C NMR (DMSO-d**_**6**_ **) δ:** 36.10, 46.95, 56.31, 107.65, 112.63, 116.21, 127.09, 127.81, 127.90, 128.79, 128.95, 129.02, 129.81, 136.24, 136.97, 149.45, 157.56, 160.88, 164.98. **HRMS (ESI** ^**+**^ **) [M + H]** ^**+**^: calcd for C_23_H_21_N_2_O_2_S: 389.1318, found: 389.1311.

##### 4.1.2.20 *4-(((3-Benzyl-7-methoxy-4-oxo-3,4-dihydroquinazolin-2-yl)­thio)­methyl)­benzonitrile* (**4b**)

White solid; Yield: 90%; Melting Point: 196–198 °C. ^**1**^ **H NMR (DMSO-d**_**6**_ **) δ:** 3.92 (*s*, 3H, OCH_3_), 4.59 (*s*, 2H, CH_2_), 5.27 (*s*, 2H, CH_2_), 7.07 (*d*, 2H, *J =* 8.58 Hz, H–Ar), 7.18 (*d*, 2H, *J =* 7.15 Hz, H–Ar), 7.25–7.33 (*m*, 3H, H–Ar), 7.73 (*dd*, 4H, *J =* 8.29 and 21.64 Hz, H–Ar), 8.00 (*d*, 1H, *J =* 8.43 Hz, H–Ar). ^**13**^ **C NMR (DMSO-d**_**6**_ **) δ:** 35.31, 46.97, 56.34, 107.73, 110.47, 112.63, 116.23, 119.20, 127.07, 127.85, 128.82, 129.05, 130.78, 132.72, 136.18, 143.74, 149.30, 157.02, 160.85, 164.99. **HRMS (ESI** ^**+**^ **) [M + H]** ^**+**^: calcd for C_24_H_20_N_3_O_2_S: 414.1271, found: 414.1270.

##### 4.1.2.21 *4-(((3-Benzyl-7-methoxy-4-oxo-3,4-dihydroquinazolin-2-yl)­thio)­methyl)­benzoic acid* (**4c**)

White solid; Yield: 50%; Melting Point: 227–230 °C. ^**1**^ **H NMR (DMSO-d**_**6**_ **) δ:** 3.92 (*s*, 3H, OCH_3_), 4.58 (*s*, 2H, CH_2_), 5.28 (*s*, 2H, CH_2_), 7.05–7.09 (*m*, 2H, H–Ar), 7.19 (*d*, 2H, *J =* 6.99 Hz, H–Ar), 7.25–7.30 (*m*, 3H, H–Ar), 7.58 (*d*, 2H, *J =* 8.11 Hz, H–Ar), 7.87 (*d*, 2H, *J =* 8.11 Hz, H–Ar), 8.01 (*d*, 2H, *J =* 8.53 Hz, H–Ar), 12.94 (*s*, 1H, OH). ^**13**^ **C NMR (DMSO-d**_**6**_ **) δ:** 35.52, 46.96, 56.32, 98.80, 107.67, 112.61, 116.25, 127.07, 127.54, 127.83, 128.68, 128.79, 129.03, 129.86, 129.97, 130.19, 136.20, 142.59, 149.37, 157.20, 160.87, 164.99, 167.50, 176.25. **HRMS (ESI** ^**+**^ **) [M + H]** ^**+**^: calcd for C_24_H_21_N_2_O_4_S: 433.1217, found: 433.1220.

##### 4.1.2.22 *3-Benzyl-7-methoxy-2-((naphthalen-2-ylmethyl)­thio)­quinazolin-4­(3H)-one* (**4d**)

White solid; Yield: 96%; Melting Point: 144–146 °C. ^**1**^ **H NMR (DMSO-d**_**6**_ **) δ:** 3.94 (*s*, 3H, OCH_3_), 4.70 (*s*, 2H, CH_2_), 5.28 (*s*, 2H, CH_2_), 7.06 (*dd*, 1H, *J =* 2.35 and 6.49 Hz, H–Ar), 7.16–7.20 (*m*, 3H, H–Ar), 7.24–7.31 (*m*, 3H, H–Ar), 7.47–7.50 (*m*, 3H, H–Ar), 7.58 (*d*, 1H, *J =* 8.42 Hz, H–Ar), 7.85–7.87 (*m*, 3H, H–Ar), 8.00 (*d*, 2H, *J =* 9.11 Hz, H–Ar). ^**13**^ **C NMR (DMSO-d**_**6**_ **) δ:** 36.34, 46.96, 56.36, 107.63, 112.64, 116.26, 126.54, 126.81, 127.10, 127.82, 127.86, 128.02, 128.52, 128.62, 128.78, 129.02, 132.63, 133.18, 134.55, 136.25, 149.47, 157.44, 160.89, 165.00. **HRMS (ESI** ^**+**^ **) [M + H]** ^**+**^: calcd for C_27_H_23_N_2_O_2_S: 439.1475, found: 439.1475.

##### 4.1.2.23 *3-Benzyl-7-methoxy-2-((4-(trifluoromethyl)­benzyl)­thio)­quinazolin-4­(3H)-one* (**4e**)

White solid; Yield: 85%; Melting Point: 119–124 °C. ^**1**^ **H NMR (DMSO-d**_**6**_ **) δ:** 3.92 (*s*, 3H, OCH_3_), 4.60 (*s*, 2H, CH_2_), 5.28 (*s*, 2H, CH_2_), 7.07 (*d*, 2H, *J* = 11.92 Hz, H–Ar), 7.19 (*d*, 2H, *J =* 6.97 Hz, H–Ar), 7.25–7.33 (*m*, 3H, H–Ar), 6.69 (*q*, 4H, *J =* 8.49 and 3.58 Hz, H–Ar), 8.01 (*d*, 1H, *J =* 8.69 Hz, H–Ar). ^**13**^ **C NMR (DMSO-d**_**6**_ **) δ:** 35.19, 46.97, 56.33, 107.71, 112.63, 116.23, 125.67, 125.72, 127.07, 127.84, 128.51, 128.81, 129.03, 130.60, 136.19, 142.64, 149.34, 157.12, 160.87, 164.99. **HRMS (ESI** ^**+**^ **) [M + H]** ^**+**^: calcd for C_24_H_20_F_3_N_2_O_2_S: 457.1192, found: 457.1189.

##### 4.1.2.24 *3-Benzyl-2-((4-bromobenzyl)­thio)-7-methoxyquinazolin-4­(3H)-one* (**4f**)

White solid; Yield: 98%; Melting Point: 130 °C. ^**1**^ **H NMR (DMSO-d**_**6**_ **) δ:** 3.92 (*s*, 3H, OCH_3_), 4.49 (*s*, 2H, CH_2_), 5.27 (*s*, 2H, CH_2_), 7.07 (*d*, 2H, *J =* 8.72 Hz, H–Ar), 7.18 (*d*, 2H, *J =* 6.98 Hz, H–Ar), 7.25–7.32 (*m*, 3H, H–Ar), 7.46 (*dd*, 4H, *J =* 8.26 and 12.68 Hz, H–Ar), 8.01 (*d*, 1H, *J =* 8.45 Hz, H–Ar). ^**13**^ **C NMR (DMSO-d**_**6**_ **) δ:** 35.20, 46.96, 56.33, 107.69, 112.62, 116.21, 120.99, 127.80, 127.83, 128.80, 129.03, 131.75, 132.02, 136.21, 136.93, 149.37, 157.28, 160.86, 164.98. **HRMS (ESI** ^**+**^ **) [M + H]** ^**+**^: calcd for C_23_H_19_BrN_2_O_2_S: 469.0408, found: 469.0403

##### 4.1.2.25 *3-Benzyl-2-((2-(4-chlorophenyl)-2-oxoethyl)­thio)­quinazolin-4­(3H)-one* (**5a**)

White solid; Yield: 99%; Melting Point: 180–181 °C. ^**1**^ **H NMR (DMSO-d**_**6**_ **) δ:** 4.83 (*s*, 2H, CH_2_), 5.37 (*s*, 2H, CH_2_), 6.99 (*d*, 1H, *J =* 8.14 Hz, H–Ar), 7.29–7.45 (*m*, 6H, H–Ar), 7.66–7.72 (*m*, 3H, H–Ar), 8.07 (*d*, 1H, *J =* 7.72 Hz, H–Ar), 8.12 (*d*, 2H, *J =* 8.49 Hz, H–Ar). ^**13**^ **C NMR (DMSO-d**_**6**_ **) δ:** 39.38, 47.65, 119.01, 125.89, 126.61, 127.11, 127.31, 127.98, 129.08, 129.40, 130.70, 135.40, 135.59, 136.01, 138.83, 146.98, 156.76, 161.19, 193.24. **HRMS (ESI** ^**+**^ **) [M + H]** ^**+**^: calcd for C_23_H_17_ClN_2_O_2_S: 421.0778, found: 421.0778.

##### 4.1.2.26 *3-Benzyl-2-((2-oxo-2-(p-tolyl)­ethyl)­thio)­quinazolin-4­(3H)-one* (**5b**)

Yellow solid; Yield: 94%; Melting Point: 150–152 °C. ^**1**^ **H NMR (DMSO-d**_**6**_ **) δ:** 2.42 (*s*, 3H, CH_3_), 4.83 (*s*, 2H, CH_2_), 5.38 (*s*, 2H, CH_2_), 7.04 (*d*, 1H, *J =* 8.04 Hz, H–Ar), 7.27–7.44 (*m*, 8H, H–Ar), 7.69 (*t*, 1H, *J =* 8.01 Hz, H–Ar), 8.00 (*d*, 2H, *J =* 8.06 Hz, H–Ar), 8.07 (*d*, 1H, *J =* 7.74 Hz, H–Ar). ^**13**^ **C NMR (DMSO-d**_**6**_ **) δ:** 21.71, 39.52, 47.62, 119.02, 125.98, 126.55, 127.08, 127.33, 127.96, 128.90, 129.08, 129.80, 134.31, 135.35, 136.60, 144.41, 147.04, 156.85, 161.22, 193.40. **HRMS (ESI** ^**+**^ **) [M + H]** ^**+**^: calcd for C_24_H_21_N_2_O_2_S: 401.1318; found: 401.1322.

##### 4.1.2.27 *3-Benzyl-2-((2-(4-methoxyphenyl)-2-oxoethyl)­thio)­quinazolin-4­(3H)-one* (**5c**)

White solid; Yield: 93%; Melting Point: 160–163 °C. ^**1**^ **H NMR (DMSO-d**_**6**_ **) δ:** 3.88 (*s*, 3H, OCH_3_), 4.81 (*s*, 2H, CH_2_), 5.38 (*s*, 2H, CH_2_), 7.09 (*t*, 3H, *J =* 8.99 Hz, H–Ar), 7.29–7.36 (*m*, 5H, H–Ar), 7.43 (*d*, 1H, *J =* 7.46 Hz, H–Ar), 7.70 (*t*, 1H, *J =* 7.15 Hz, H–Ar), 8.08 (*t*, 3H, *J =* 8.58 Hz, H–Ar). ^**13**^ **C NMR (DMSO-d**_**6**_ **) δ:** 38.96, 39.10, 48.18, 125.87, 127.05, 127.49, 128.85, 129.33, 130.66, 130.86, 133.10, 135.75, 136.82, 138.67, 143.77, 156.68, 193.56. **HRMS (ESI** ^**+**^ **) [M + H]** ^**+**^: calcd for C_24_H_20_N_2_O_3_S: 417.1228, found: 417.1269

##### 4.1.2.28 *2-((3-Benzyl-4-imino-3,4-dihydroquinazolin-2-yl)­thio)-1-(4-chlorophenyl)­ethanone* (**6a**)

**White solid; Yield:**99%; Melting Point: 169–171 °C. ^**1**^ **H NMR (DMSO-d**_**6**_ **) δ:** 4.71 (*s*, 2H, CH_2_), 5.46 (*s*, 2H, CH_2_), 6.76 (*d*, 1H, *J =* 7.93 Hz, H–Ar), 7.24–7.36 (*m*, 6H, H–Ar), 7.46 (*t*, 1H, *J =* 7.59 Hz, H–Ar), 7.66 (*d*, 2H, *J =* 8.46 Hz, H–Ar), 8.12 (*t*, 3H, *J =* 7.31 Hz, H–Ar), 8.86 (*s*, 1H, H–Ar). ^**13**^ **C NMR (DMSO-d**_**6**_ **) δ:** 38.97, 48.20, 118.56, 125.84, 125.89, 127.04, 127.50, 128.86, 129.33, 130.66, 131.49, 133.12, 135.74, 136.73, 138.67, 143.78, 156.66, 193.56. **HRMS (ESI** ^**+**^ **) [M + H]** ^**+**^: calcd for C_23_H_18_ClN_3_OS: 420.0932, found: 420.0932.

##### 4.1.2.29 *2-((3-Benzyl-4-imino-3,4-dihydroquinazolin-2-yl)­thio)-1-(p-tolyl)­ethanone* (**6b**)

Yellow solid; Yield: 68%; Melting Point: 153–156 °C. ^**1**^ **H NMR (DMSO-d**_**6**_ **) δ:** 2.42 (*s*, 3H, CH_3_), 4.72 (*s*, 2H, CH_2_), 5.47 (*s*, 2H, CH_2_), 6.8 (*d*, 1H, *J =* 8.06 Hz, H–Ar), 7.24–7.33 (*m*, 6H, H–Ar), 7.38 (*d*, 2H, *J =* 7.97 Hz, H–Ar), 7.45 (*t*, 1H, *J =* 7.62 Hz, H–Ar), 7.99 (*d*, 2H, *J =* 8.01 Hz, H–Ar), 8.13 (*d*, 1H, *J =* 7.97 Hz, H–Ar), 8.85 (*s*, 1H, NH). ^**13**^ **C NMR (DMSO-d**_**6**_ **) δ:** 21.70, 39.10, 48.14, 118.53, 125.82, 125.99, 127.07, 127.47, 128.87, 129.75, 133.05, 134.45, 136.87, 143.83, 144.24, 155.90, 156.74, 193.73. **HRMS (ESI** ^**+**^ **) [M + H]** ^**+**^: calcd for C_24_H_21_N_3_OS: 400.1439, found: 400.1479.

##### 4.1.2.30 *2-((3-Benzyl-4-imino-3,4-dihydroquinazolin-2-yl)­thio)-1-(4-methoxyphenyl)­ethanone* (**6c**)

Yellow solid; Yield: 76%; Melting Point: 182–183 °C. ^**1**^ **H NMR (DMSO-d**_**6**_ **) δ:** 3.87 (*s*, 3H, OCH_3_), 4.70 (*s*, 2H, CH_2_), 5.48 (*s*, 2H, CH_2_), 6.86 (*d*, 1H, *J =* 7.99 Hz, H–Ar), 7.10 (*d*, 2H, *J =* 8.69 Hz, H–Ar), 7.24–7.36 (*m*, 6H, H–Ar), 7.47 (*t*, 1H, *J =* 7.56 Hz, H–Ar), 8.07 (*d*, 2H, *J =* 8.64 Hz, H–Ar), 8.13 (*d*, 1H, *J =* 7.87 Hz, H–Ar), 8.84 (*s*, 1H, NH). ^**13**^ **C NMR (DMSO-d**_**6**_ **) δ:** 38.92, 48.12, 56.06, 114.39, 118.59, 125.81, 126.04, 127.47, 128.84, 129.74, 131.15, 133.06, 136.84, 143.85, 156.78, 163.73, 192.52. **HRMS (ESI** ^**+**^ **) [M + H]** ^**+**^: calcd for C_24_H_22_N_3_O_2_S: 416.1427, found: 416.1423.

##### 4.1.2.31 *3-Benzyl-7-chloro-2-((2-(4-chlorophenyl)-2-oxoethyl)­thio)­quinazolin-4­(3H)-one* (**7a**)

White solid; Yield: 84%; Melting Point: 188–189 °C. ^**1**^ **H NMR (DMSO-d**_**6**_ **) δ:** 4.84 (*s*, 2H, CH_2_), 5.35 (*s*, 2H, CH_2_), 5.94 (*d*, 1H, *J =* 1.55 Hz, H–Ar), 7.29–7.38 (*m*, 5H, H–Ar), 7.45 (*dd*, 1H, *J =* 1.63 and 6.90 Hz, H–Ar), 7.68 (*d*, 2H, *J =* 8.46 Hz, H–Ar), 8.05 (*d*, 1H, *J =* 8.57 Hz, H–Ar), 8.11 (*d*, 2H, *J =* 8.50 Hz, H–Ar). ^**13**^ **C NMR (DMSO-d**_**6**_ **) δ:** 39.51, 47.78, 117.87, 124.96, 126.79, 127.33, 128.04, 129.11, 129.29, 129.39, 130.72, 135.55, 135.74, 138.90, 139.82, 147.83, 158.79, 160.60, 193.17. **HRMS (ESI** ^**+**^ **) [M + H]** ^**+**^: calcd for C_23_H_17_Cl_2_N_2_O_2_S: 455.0382, found: 455.0390.

##### 4.1.2.32 *3-Benzyl-7-chloro-2-((2-oxo-2-(p-tolyl)­ethyl)­thio)­quinazolin-4­(3H)-one* (**7b**)

White solid; Yield: 91%; Melting Point: 178 °C. ^**1**^ **H NMR (DMSO-d**_**6**_ **) δ:** 2.43 (*s*, 3H, CH_3_), 4.83 (*s*, 2H, CH_2_), 5.36 (*s*, 2H, CH_2_), 6.97 (*d*, 1H, *J =* 1.45 Hz, H–Ar), 7.30–7.47 (*m*, 8H, H–Ar), 8.03 (*dd*, 3H, *J =* 8.54 and 7.91 Hz, H–Ar). ^**13**^ **C NMR (DMSO-d**_**6**_ **) δ:** 21.70, 39.37, 47.75, 117.88, 125.05, 126.74, 127.34, 128.03, 128.93, 129.10, 129.27, 129.80, 134.33, 135.78, 139.79, 144.49, 147.89, 158.90, 160.63, 193.42. **HRMS (ESI** ^**+**^ **) [M + H]** ^**+**^: calcd for C_24_H_20_ClN_2_O_2_S: 435.0929, found: 435.0933.

##### 4.1.2.33 *3-Benzyl-7-chloro-2-((2-(4-methoxyphenyl)-2-oxoethyl)­thio)­quinazolin-4­(3H)-one* (**7c**)

White solid; Yield: 84%; Melting Point: 177 °C. ^**1**^ **H NMR (DMSO-d**_**6**_ **) δ:** 3.88 (*s*, 3H, OCH_3_), 4.82 (*s*, 2H, CH_2_), 5.36 (*s*, 2H, CH_2_), 7.04 (*d*, 1H, *J =* 1.68 Hz, H–Ar), 7.11 (*d*, 2H, *J =* 8.75 Hz, H–Ar), 7.30–7.38 (*m*, 7H, H–Ar), 7.45 (*dd*, 1H, *J =* 1.87 and 6.70 Hz, H–Ar), 8.07 (*t*, 3H, *J =* 8.69 Hz, H–Ar). ^**13**^ **C NMR (DMSO-d**_**6**_ **) δ:** 39.51, 47.73, 56.12, 114.46, 117.88, 125.11, 126.74, 127.33, 128.02, 129.10, 129.27, 129.58, 131.23, 135.80, 139.81, 147.92, 158.95, 160.65, 163.91, 192.15. **HRMS (ESI** ^**+**^ **) [M + H]** ^**+**^: calcd for C_24_H_20_ClN_2_O_3_S: 451.0878, found: 451.0879.

##### 4.1.2.34 *3-Benzyl-2-((2-(4-chlorophenyl)-2-oxoethyl)­thio)-7-methoxyquinazolin-4­(3H)-one* (**8a**)

White solid; Yield: 69%; Melting Point: 170 °C. ^**1**^ **H NMR (DMSO-d**_**6**_ **) δ:** 3.69 (*s*, 3H, OCH_3_), 4.76 (*s*, 2H, CH_2_), 5.32 (*s*, 2H, CH_2_), 6.16 (*d*, 1H, *J =* 2.13 Hz, H–Ar), 6.98 (*dd*, 1H, *J =* 2.13 and 6.60 Hz, H–Ar), 7.27–7.38 (*m*, 5H, H–Ar), 7.69 (*d*, 2H, *J =* 8.38 Hz, H–Ar), 7.94 (*d*, 1H, *J =* 8.80 Hz, H–Ar), 8.14 (*d*, 2H, *J =* 8.48 Hz, H–Ar). ^**13**^ **C NMR (DMSO-d**_**6**_ **) δ:** 38.77, 47.45, 55.91, 106.59, 112.41, 116.21, 127.29, 127.95, 128.79, 129.07, 129.32, 130.79, 136.01, 136.13, 138.74, 149.05, 157.50, 160.65, 164.66, 193.76. **HRMS (ESI** ^**+**^ **) [M + H]** ^**+**^: calcd for C_24_H_20_ClN_2_O_3_S: 451.0878, found: 451.0885.

##### 4.1.2.35 *3-Benzyl-7-methoxy-2-((2-oxo-2-(p-tolyl)­ethyl)­thio)­quinazolin-4­(3H)-one* (**8b**)

White solid; Yield: 99%; Melting Point: 152–153 °C. ^**1**^ **H NMR (DMSO-d**_**6**_ **) δ:** 2.41 (*s*, 3H, CH_3_), 3.68 (*s*, 3H, OCH_3_), 4.77 (*s*, 2H, CH_2_), 5.33 (*s*, 3H, CH_2_), 6.27 (*d*, 1H, *J =* 2.02 Hz, H–Ar), 6.98 (*dd*, 1H, *J =* 2.05 and 6.77 Hz, H–Ar), 7.27–7.42 (*m*, 7H, H–Ar), 7.95 (*d*, 1H, *J =* 8.79 Hz, H–Ar), 8.02 (*d*, 2H, *J =* 7.94 Hz, H–Ar). ^**13**^ **C NMR (DMSO-d**_**6**_ **) δ:** 21.64, 38.99, 47.40, 55.96, 106.81, 112.42, 116.14, 127.30, 127.93, 128.75, 129.00, 129.07, 129.72, 134.68, 136.18, 144.30, 149.13, 157.54, 160.70, 164.66, 193.91. **HRMS (ESI** ^**+**^ **) [M + H]** ^**+**^: calcd for C_25_H_23_N_2_O_3_S: 431.1424, found: 431.1429.

##### 4.1.2.36 *3-Benzyl-7-methoxy-2-((2-(4-methoxyphenyl)-2-oxoethyl)­thio)­quinazolin-4­(3H)-one* (**8c**)

White solid; Yield: 90%; Melting Point: 167–168 °C. ^**1**^ **H NMR (DMSO-d**_**6**_ **) δ:** 3.70 (*s*, 3H, OCH_3_), 3.87 (*s*, 3H, OCH_3_), 4.76 (*s*, 2H, CH_2_), 5.33 (*s*, 2H, CH_2_), 6.32 (*d*, 1H, *J =* 1.95 Hz, H–Ar), 6.99 (*dd*, 1H, *J =* 2.03 and 6.83 Hz, H–Ar), 7.12 (*d*, 2H, *J =* 8.70 Hz, H–Ar), 7.28–7.38 (*m*, 5H, H–Ar), 7.95 (*d*, 1H, *J =* 8.78 Hz, H–Ar), 8.10 (*d*, 2H, *J =* 8.63 Hz, H–Ar). ^**13**^ **C NMR (DMSO-d**_**6**_ **) δ:** 38.88, 47.37, 55.98, 56.08, 106.89, 112.44, 114.36, 116.14, 127.30, 127.93, 128.76, 129.07, 129.98, 131.29, 136.20, 149.15, 157.59, 160.72, 163.78, 164.68, 192.69. **HRMS (ESI** ^**+**^ **) [M + H]** ^**+**^: calcd for C_25_H_23_N_2_O_4_S: 447.1373, found: 4471376.

### 4.2 Biological Evaluation

#### 4.2.1 Antimalarial Activity against Asexual Blood Stages of *Plasmodium falciparum*

The tested compounds were dissolved in DMSO (Sigma-Aldrich) to obtain a 5 mM solution, from which two additional solutions were prepared at 100 μM and 10 μM by dilution with RPMI-1640 medium (Invitrogen), supplemented with AlbuMAX II (Invitrogen). In parallel, the parasite cultures of *P. falciparum* strain 3D7HT-GFP (chloroquine-sensitive, expressing Green Fluorescent Protein), obtained through BEI Resources, NIAID, NIH (MR4, ATCC, Manassas, Virginia), were prepared, which were provided by Andrew M. Talman, Robert E. Sinden, David Walliker, and Didier Ménard. The parasites were cultivated at 5% hematocrit and incubated at 37 °C under an atmosphere enriched with 5% CO_2_.^46,47^ Thus, unsynchronized cultures were incubated in a 96-well flat-bottom plate at 1.2% hematocrit and 1.0% parasitemia, in the presence and absence of 1 μM and 10 μM of each compound, for 72 h at 37 °C and 5% CO_2_. Chloroquine (Sigma-Aldrich) was used as a standard drug at the same concentrations as the other compounds. The parasite growth was determined by flow cytometry (Beckman Coulter, CytoFLEX) with a 96-well plate reader, using FL-1, with an excitation wavelength of 488 nm, for the detection of GFP. The flow cytometry data were analyzed using *FlowJo* software (Tree Star Inc.) to calculate compound inhibition percentages.

The most promising compounds in the screening, with at least 70% inhibition at the highest concentration (10 μM, with a 1:3 dilution), were selected for further development by determining half-maximal inhibitory concentrations (IC_50_).^48^ Then, unsynchronized cultures of 3D7HT-GFP were incubated at 1.2% hematocrit and 1.0% parasitemia, with serial 3-fold dilutions of the compounds ranging from 10 μM to 0.17 nM, in 96-well flat-bottom plates for 72 h at 37 °C and 5% CO_2_. Parasite growth was quantified by flow cytometry (Beckman Coulter, CytoFLEX) using the FL-1 channel to detect GFP. Data analysis was performed with *FlowJo* software (Tree Star Inc.), and the IC_50_ were calculated using *GraphPad Prism 5* (trial version), with the mean IC_50_ values obtained from at least two independent experiments, each conducted in duplicate.

#### 4.2.2 Antileishmanial Susceptibility Assay against *Leishmania donovani*

The compounds **3d**, **3e**, **4a**, and **4d** were solubilized in dimethyl sulfoxide (DMSO – Merck, Darmstadt, Germany). Working solutions were prepared with a maximum of 1% DMSO, using Amphotericin B (Gibco) as a positive control. In parallel, promastigote forms of *L. donovani*­(MHOM/BD/2006/BD14) were maintained at 24 °C in M199 medium (Sigma), supplemented with 10% (v/v) fetal bovine serum (FBS, Sigma, Darmstadt, Germany), 2.5 mg/mL hemin (Sigma), HEPES 1 M pH 7.4 (Sigma), and 1% (v/v) penicillin–streptomycin (Sigma). Promastigote cultures underwent fewer than 10 *in vitro* passages to maintain strain virulence. Promastigotes were used for experiments at the end of the log phase (5 to 6 days).^49^

Promastigotes of *L. donovani* were seeded in 96-well flat-bottom culture plates (VWR) at a density of 5 × 10^6^ parasites/mL in M199 medium, with concentrations of 5, 10, and 20 μM of each compound, including amphotericin B (at 2 μM). A blank (medium alone) and an untreated control (parasites with M199 medium plus 1% DMSO as the carrier solution) were included. All compounds were plated in triplicate and incubated for 48 h at 24 °C ± 1 °C in a final volume of 100 μL. At the end of the incubation, to evaluate parasite viability, 10 μL/well of MTT (3-(4,5-dimethylthiazol-2-yl)-2,5-diphenyltetrazolium bromide, 5 mg/mL in PBS) was added, followed by 2–4 h of incubation at 37 °C ± 1 °C. After centrifugation at 1000 × g (20 min, 0 °C), the medium was removed, and the formazan crystals were solubilized in DMSO. The optical density at 595 nm was measured using the Synergy HTX Multi-Mode Microplate Reader (Dynex Technologies, Chantilly, VA, USA). Finally, the percentage inhibition and survival were calculated in *Microsoft Excel* using the results of two independent experiments performed in triplicate.^49^

#### 4.2.3 Cell Assay against *Trypanosoma congolense*

*T. congolense* savannah IL3000 SM^50^ bloodstream-form parasites were cultured in TcBSF1 medium^50^ supplemented with 10% goat serum on 10 mm-diameter Petri dishes at 34 °C, until reaching a concentration of 3 × 10^6^ parasites/mL. Parasites (3 × 10^5^ parasites/mL) were incubated with DMSO (control) or 10 μM of the compounds **3d**, **3e**, **4a**, and **4d** for 24, 48, and 72 h, diluted in 200 μL of TcBSF1 medium, in 96-well plates. Hygromycin (1.1 μM) was used as a positive control. Parasite numbers were determined at each time point by hemocytometry, using a benchtop inverted microscope. Three independent experiments were performed in triplicate.

#### 4.2.4 Cytotoxicity Assessment in the THP-1 Cell Line

The THP-1 cell line was used to calculate the half-maximal cytotoxic concentration (CC_50_) of the quinazoline derivatives **1e**, **2e**, **2f**, **3a**, **3b**, **3d**, **3e**, **3f**, **4a**, **4d**, **4e**, **4f**, **8a**, **8b**, and **8c**. In this way, THP-1 cells were plated at a final density of 5 × 10^5^ cells/mL, in 96-well flat-bottom culture plates (VWR), using six different concentrations (serial dilutions from 100 μM to 1.062 μM) of the compounds and amphotericin B (10, 5, 2.5, 1.25, 0.625, 0.313 μM). A blank (RPMI medium alone) and an untreated control (parasites with RPMI medium plus 1% DMSO as the vehicle solution) were also included. All the compounds were plated in quadruplicate and incubated for 48 h at 37 °C ± 1 °C, 5% CO_2_. Following the incubation period, 10 μL/well of MTT was added as described in Section 4.2.2. After absorbance readings, the CC_50_ values of each compound were determined with GraphPad Prism V 9.0 (Dotmatics, San Diego, CA, USA) by fitting the data as a nonlinear regression with a variable slope using a dose–response inhibitory model. At least three independent assays were performed.^49^

### 4.3 Computational Experiments

#### 4.3.1 Molecular Docking

The experimental 3D structures of the proposed biological targets of *Plasmodium falciparum* dihydroorotate dehydrogenase – P*f*DHODH (PDB id: 3O8A); wild-type dihydrofolate reductase – *w*P*f*DHRF (PDB id: 3QGT); quadruple mutant dihydrofolate reductase – *qm*P*f*DHRF (PDB id: 3QG2); purine nucleoside phosphorylase – P*f*PNPase (PDB id: 5ZNC); prolyl-tRNA synthetase – ProRS (PDB id: 4WI1); lactate dehydrogenase – P*f*LDH (PDB id: 1U4O); falcipain-2 – P*f*FP-2 (PDB id: 6JW9); falcipain-3 – P*f*FP-3 (PDB id: 3BWK); falcilysin – P*f*FLN (PDB id: 7DI7); chloroquine resistance transporter – P*f*CTR (PDB id: 6UKJ); plasmepsin II – P*f*PMII (PDB id: 1LF2) *Plasmodium vivax*plasmepsin V – P*v*PMV (PDB id: 4ZL4); *N*-myristoyltransferase – P*v*NMT (PDB id: 4B13); *Leishmania major N*-myristoyltransferase – L*m*NMT­(PDB id: 2WSA); and *Homo sapiens* *N*-myristoyltransferase – H*s*NMT (PDB id: 3IU2) were obtained from the Research Collaboratory for Structural Bioinformatics Protein Data Bank (RCSB PDB) (https://www.rcsb.org/).^51^ For the *Trypanosoma congolense* *N*-myristoyltransferase (T*c*NMT), the model was built by homology modeling, as described in section 4.3.2.

Next, the hydrogens were added, and cocrystallized ligands, ions, and water molecules were removed and then redocked using the *GOLD* software^52^ with the Chemical Piecewise Linear Potential (ChemPLP) as the scoring function to obtain fit scores and binding modes. For P*f*FP-3 and P*f*CTR (PDB IDs 3BWK and 6UKJ, respectively), GoldScore was used as the scoring function. The best binding pose was chosen for each ligand, and their root-mean-square deviation (RMSD) values were calculated using *GOLD* software and, for the P*f*CTR (PDB id: 6UKJ), using the *PyMOL* software. The compounds **3d**, **3e**, **4a**, and **4d** were generated using the *MarvinSketch* software,^53^ and a conformational analysis was initially carried out. Thus, ten conformations were generated for each ligand, and the conformation with the lowest energy value was chosen. Finally, these structures were minimized to correct angles and bond lengths using the *ArgusLab* software,^54^ applying the semiempirical AM1 (Austin Model 1) method.

Molecular docking studies were performed using the *GOLD* software, employing the ChemPLP scoring function after our initial redocking procedures, and the GoldScore scoring function for P*f*FP-3 and P*f*CTR (PDB ids: 3BWK and 6UKJ, respectively). A 6 Å region around the cocrystallized ligand was selected using the maximum efficiency of the genetic algorithm (GA) with 300 runs, and ten binding poses were generated for each ligand. Then, each ligand pose was aligned and selected for further analysis based on the best comparable overlap between the analogs and the cocrystallized ligand. This pose was selected for further analysis of fit score and interactions at the binding site using *Discovery Studio* software.

#### 4.3.2 Homology Modeling and Sequence Alignment

The *UniProt* database^55^ was used to obtain the sequences of P*f*NMT, L*d*NMT, and T*c*NMT. Thus, the search returned the sequences under the codes Q8ILW6, D0AB09, and G0UYG5, respectively. Next, the FASTA sequences were submitted to the SWISS-MODEL web tool,^56^ and the templates Q8ILW6.1 and G0UYG5.1, available in AlphaFold DB, were identified as P*f*NMT and T*c*NMT, respectively. In addition, template 2WUU was used for L*d*NMT, which is available in the PDB. Finally, the 3D structures were built, and the models were used in molecular dynamics simulations and molecular docking with T*c*NMT.

#### 4.3.3 Molecular Dynamics (MD) Simulations

After molecular docking, the P*f*NMT, L*d*NMT, T*c*NMT, free and in complex with **4a** or its standard compound, were chosen for MD simulations using the *GROMACS-2020* software. In this process, charges and hydrogens were added using the *DockPrep* tool in *Chimera* software. Next, the CHARMM36 force field was applied using the TIP3P solvation method. In parallel, ligand topology was generated using the *SwissParam* web server (http://www.swissparam.ch/).^57^ A 1.0 nm triclinic box was then created, and water and ions were added at physiological concentrations. This was followed by the system reaching equilibrium after 10,000 steps using the conjugate gradient method, and by the system’s total minimization after 20,000 steps. Next, NVT (constant Number of particles, Volume, and Temperature) and NPT (constant Number of particles, Pressure, and Temperature) equilibrations were performed at 300 K for 10 ns. The final simulation was conducted for 100 ns. The Root Mean Square Deviation (RMSD), Root Mean Square Fluctuation (RMSF), Radius of Gyration (*R*_g_), and Solvent Accessible Surface Area (SASA) plots were generated using the *Xmgrace* software. This protocol is consistent with previous work from our research team.^58–60^

#### 4.3.4 Binding Free Energy and Interaction Parameters by MM-PBSA Calculations

The Molecular Mechanics Poisson–Boltzmann Surface Area (MM-PBSA) method was used to calculate the Gibbs free-binding energy (Δ*G*_*binding*_) based on van der Waals and electrostatic (unbound) interactions between the ligand and its receptor during an MD simulation.^61^ For this, the Δ*G*_*binding*_ was calculated by the difference between the free energy of the complex protein–ligand (*G*_*complex*_) and the unbound protein and ligand (*G*_*protein*_ and *G*_*ligand*_) (eq 1). These individual energy values are calculated by the average potential energy of molecular mechanics in a vacuum (*E*_*MM*_) minus the entropic contribution to the free energy in the vacuum, determined by the temperature and entropy (TS), added to the solvation energy (*G*_*solvation*_) (eq 2). Furthermore, *E*_*MM*_ is the sum of bonded interactions such as dihedral, angle, bond, and improper (*E*_*bond*_), and nonbonded interactions (*E*_*non* *‑* *bonded*_), which constitute the electrostatic (*E*_*elec*_) and van der Waals interactions (*E*_*vdw*_) using the potential functions of Coulomb and Lennard–Jones, respectively (eq 3). Finally, the solvation free energy (*G*_*solvation*_) is the sum of electrostatic and nonelectrostatic contributions to the solvation free energy (*G*_*polar*_ and *G*_*non‑polar*_, respectively) (eq 4). These calculations were performed using the *g_mmpbsa* tool^62^ through the trajectory files obtained after the MD simulation, using the *GROMACS* software. Then, Δ*G_binding_* values were determined as the average free interaction and solvation energies.^63^ This protocol is based on other works from our research group.^64–68^

1 $$\Delta G_{binding}=G_{complex}-\left(G_{protein}-G_{ligand}\right)$$

2 $$G_{x}=\left\langle E_{MM}\right\rangle -TS+\left\langle G_{solvation}\right\rangle$$

3 $$E_{MM}=E_{bonded}+E_{no\mathrm{n-}bonded}=E_{bonded}+\left(E_{vdw}+E_{elec}\right)$$

4 $$G_{solvation}=G_{polar}+G_{no\mathrm{n-}polar}$$

## Supplementary Material



## Notes

Spectra of Nuclear Magnetic Resonance of ^1^H and ^1^³C of the compounds (Figures **S1**–**S74**); High-resolution mass spectrometry (HRMS) data of the compounds (Figures **S75**–**S110**); Plots of half-maximal inhibitory (IC_50_) from the antimalarial assay using *P. falciparum* 3D7HT-GFP cell line (Figures S111–S113); Plots of cytotoxic concentration (CC_50_) (Figures **S114**–**S116**); Plots of molecular dynamics simulations (Figures **S117**–**S119**) (PDF)
