# Supplementary material for: Design, Synthesis, and Antimalarial Evaluation of Novel Quinazolin-4(3H)‑one Derivatives with Molecular Modeling Insights into Target Selectivity
Source: ACS Omega. 2026 Jun 10;11(24):36346–62. doi: 10.1021/acsomega.6c04943 (PMC13294948; doi:10.1021/acsomega.6c04943)
Supplement: Supplementary file 1 [file ao6c04943_si_001.pdf]

Design, Synthesis, and Antimalarial Evaluation of Novel Quinazolin-4(3*H*)-one Derivatives with Molecular Modeling Insights into Target Selectivity

Igor José dos Santos Nascimento[1,2*], Karla Joane da Silva Menezes[2,3], Margarida Cochicho Leonardo[2], Inês Morais[2], Carolina Silva Dias Vieira[4], Sara Silva Pereira[4], Sofia Cortes[2], Rui Moreira[5], Fátima Nogueira[2], and Ricardo Olimpio de Moura[1,3].

[1]*Postgraduate Program of Pharmaceutical Sciences, Pharmacy Department, State University of Paraíba, Campina Grande-PB, Brazil;*

[2]*Global Health and Tropical Medicine (GHTM), Associate Laboratory in Translation and Innovation Towards Global Health (LA-REAL), Instituto de Higiene e Medicina Tropical (IHMT), Universidade NOVA de Lisboa (UNL), Rua da Junqueira 100, 1349-008 Lisbon, Portugal;*

[3]*Postgraduate Program in Development and Technological Innovation in Medicines, State University of Paraíba (UEPB), Campina Grande, Brazil;*

[4]*Católica Biomedical Research Centre, Católica Medical School, Universidade Católica Portuguesa, Oeiras, Portugal;*

[5]*Research Institute for medicines (iMed.ULisboa), Faculty of Pharmacy, Universidade de Lisboa, 1649-003 Lisbon, Portugal.*

*Corresponding author: igor.n@visitante.uepb.edu.br or igorjsn@hotmail.com; Tel.: (+55)8299933-5457.

SUPPLEMENTARY MATERIAL

1. Spectra of Nuclear Magnetic Resonance of ^1H and ^{13}C

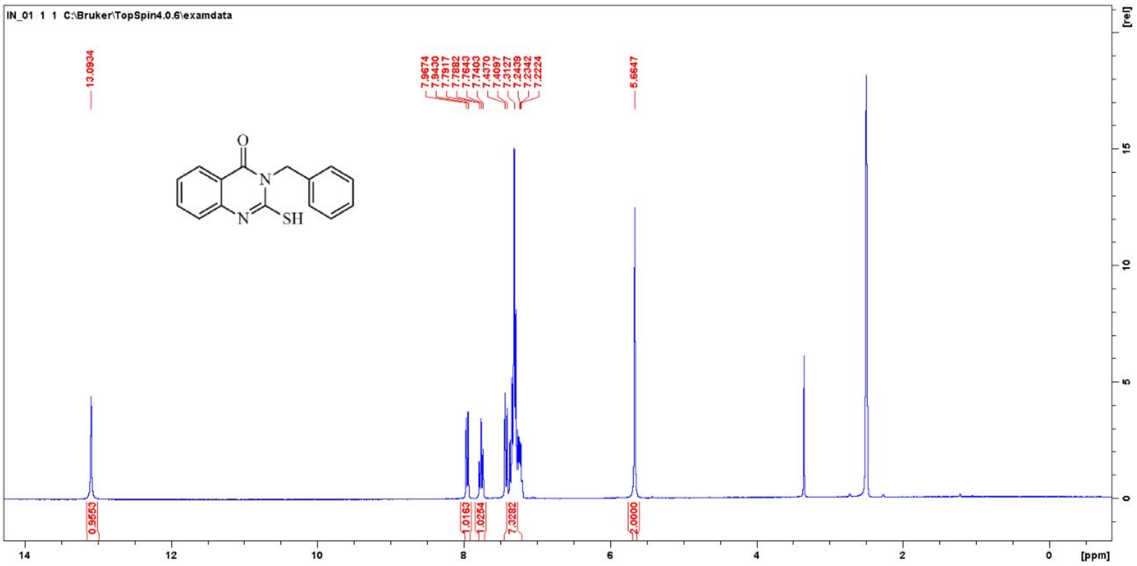

Fig. S1. ^1H-NMR for compound **1**

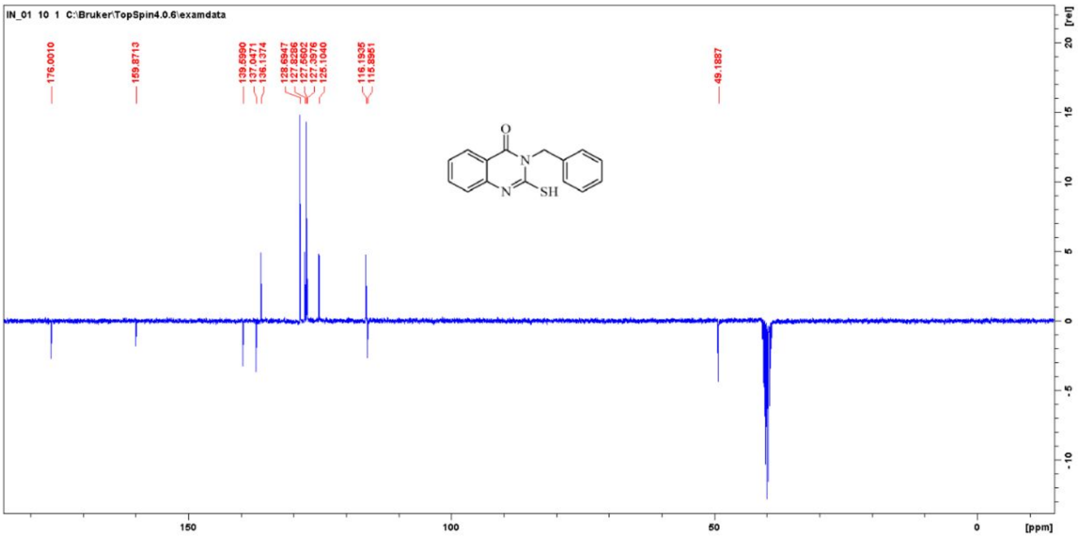

Fig. S2. ^{13}C-NMR for compound **1**

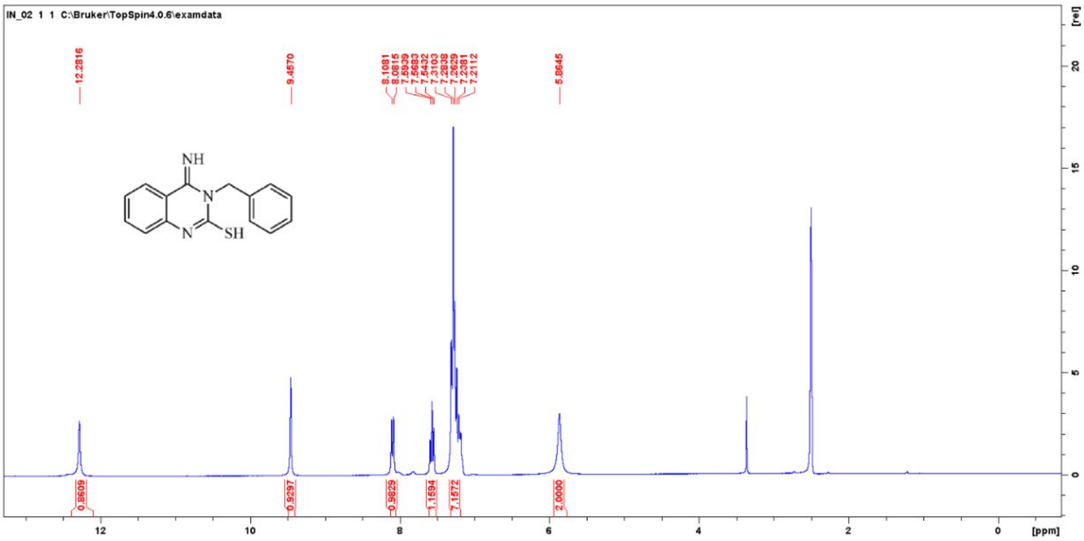

Fig. S3. ^{1}H-NMR for compound **2**

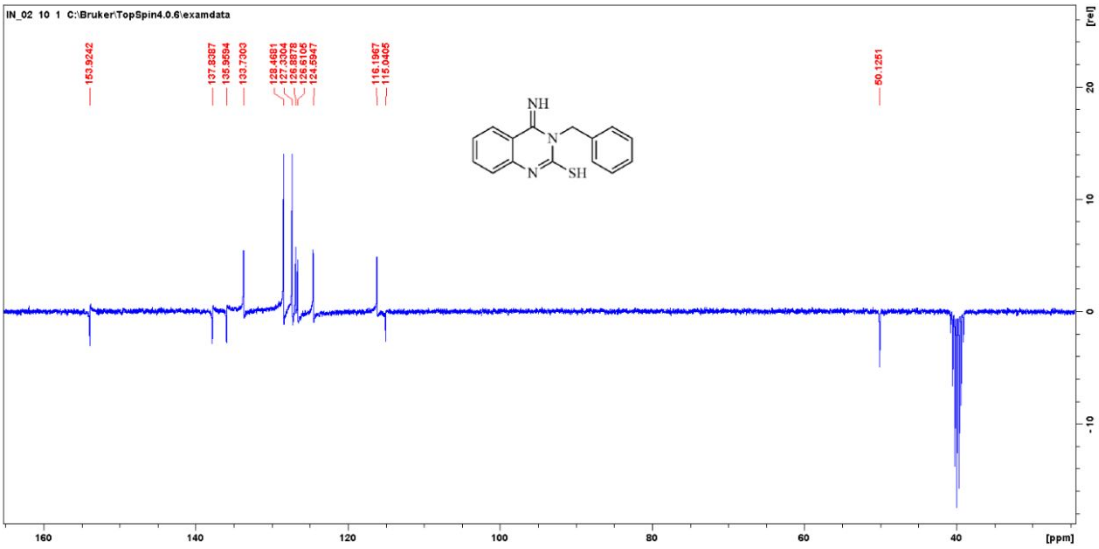

Fig. S4. ^{13}C-NMR for compound **2**

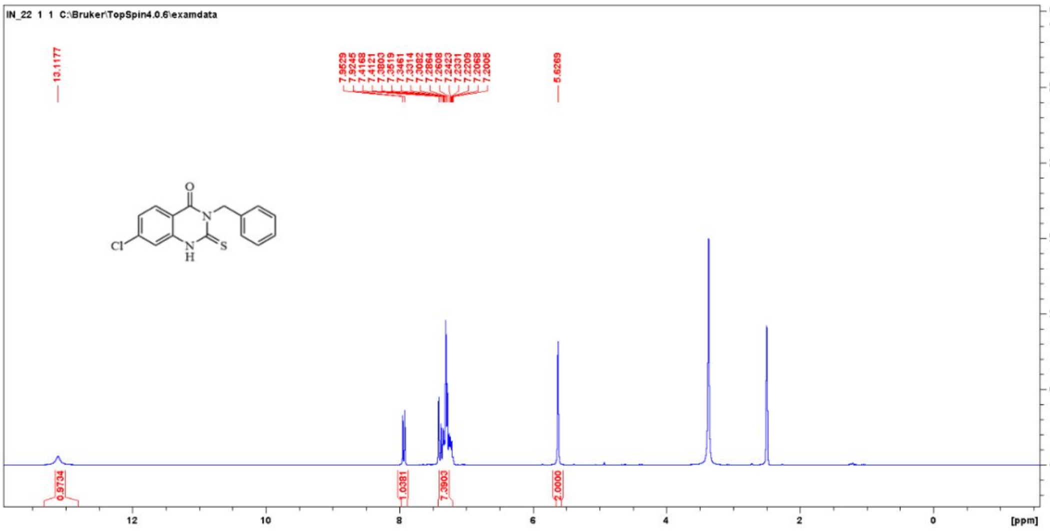

Fig. S5. ^{1}H-NMR for compound **3**

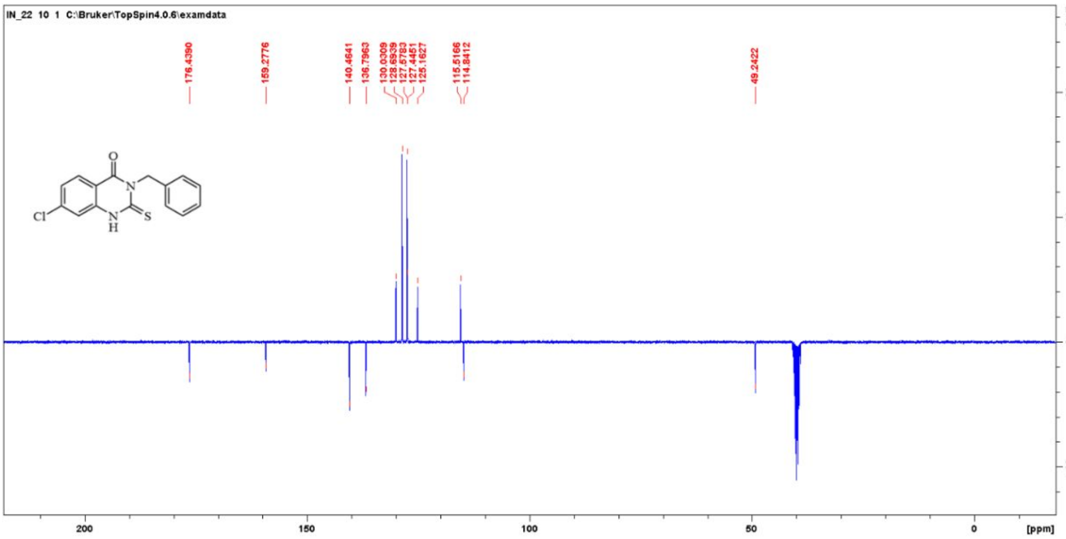

Fig. S6. ^{13}C-NMR for compound **3**

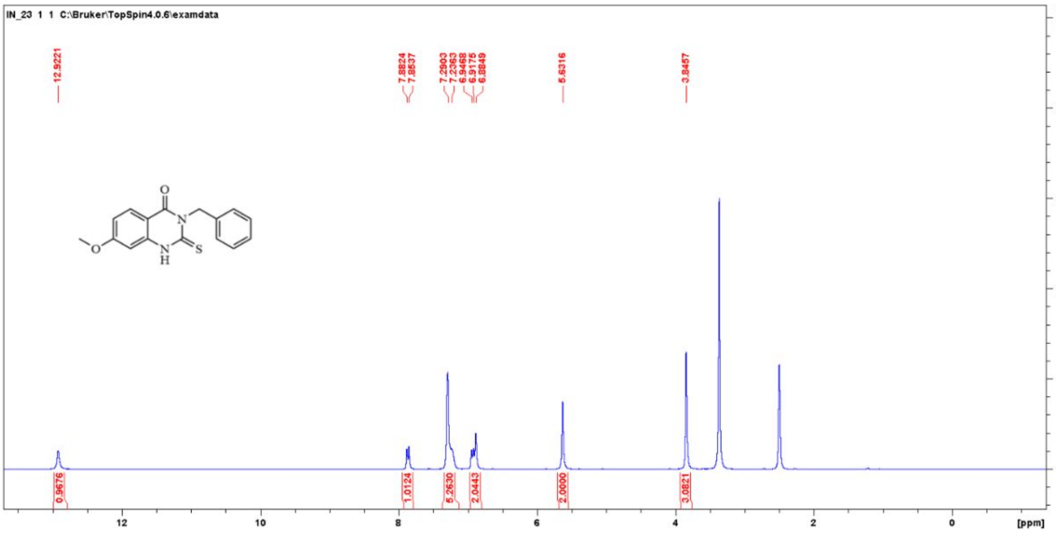

Fig. S7. ^{1}H-NMR for compound **4**

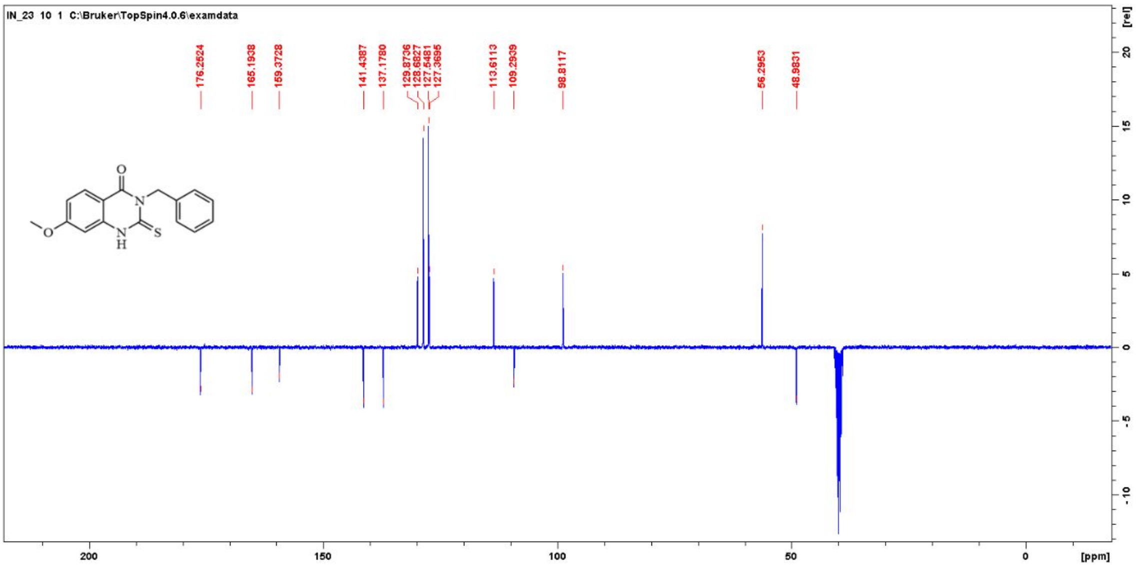

Fig. S8. ^{13}C-NMR for compound **4**

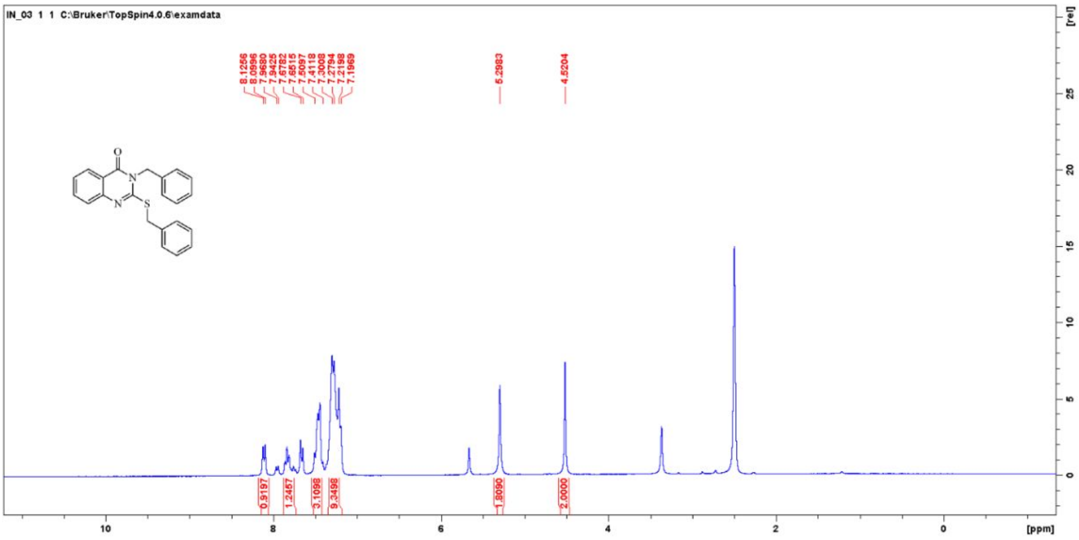

Fig. S9. ^{1}H-NMR for compound **1a**

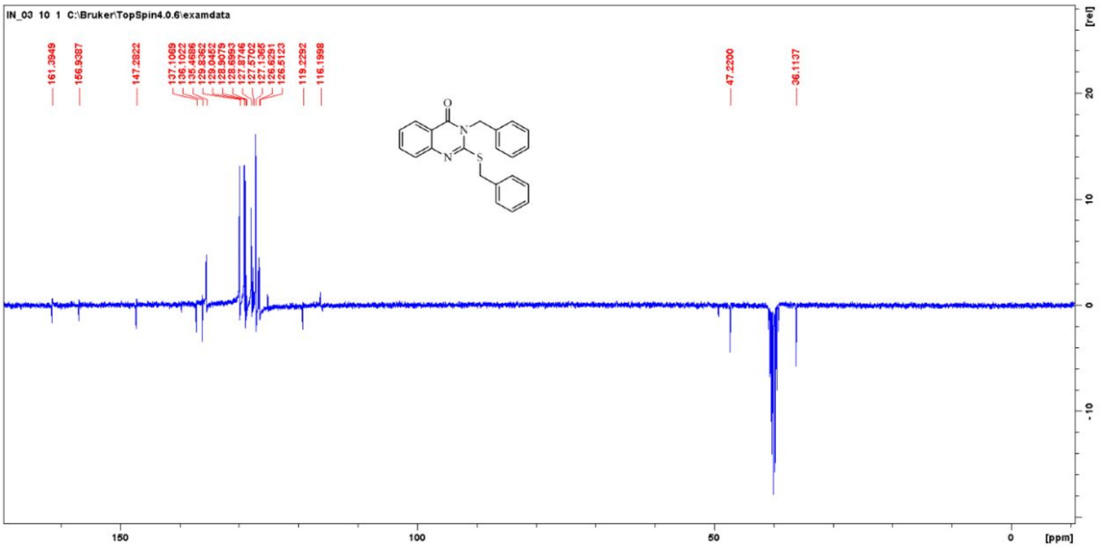

Fig. S10. ^{13}C-NMR for compound **1a**

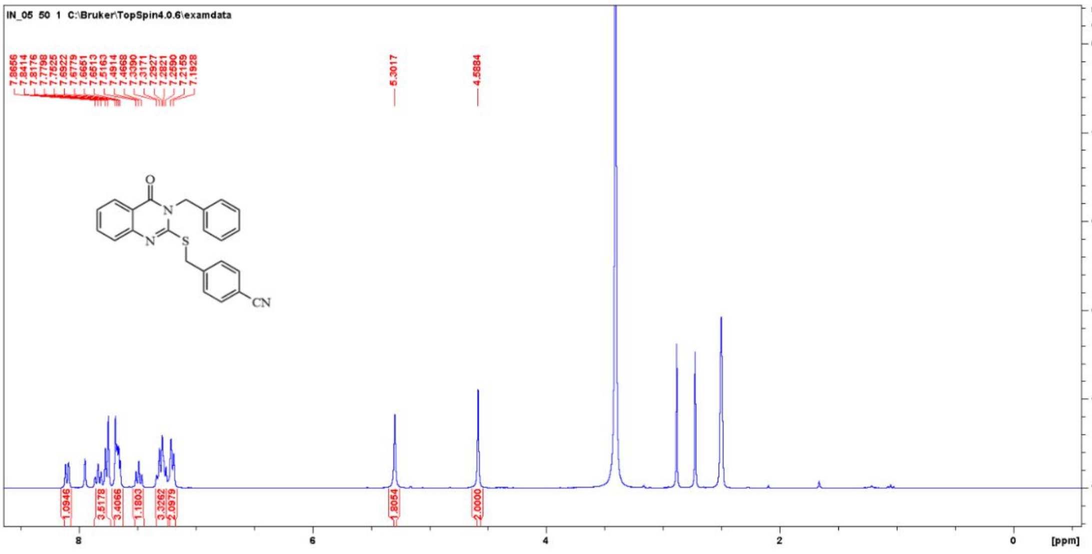

Fig. S11. ^{1}H-NMR for compound **1b**

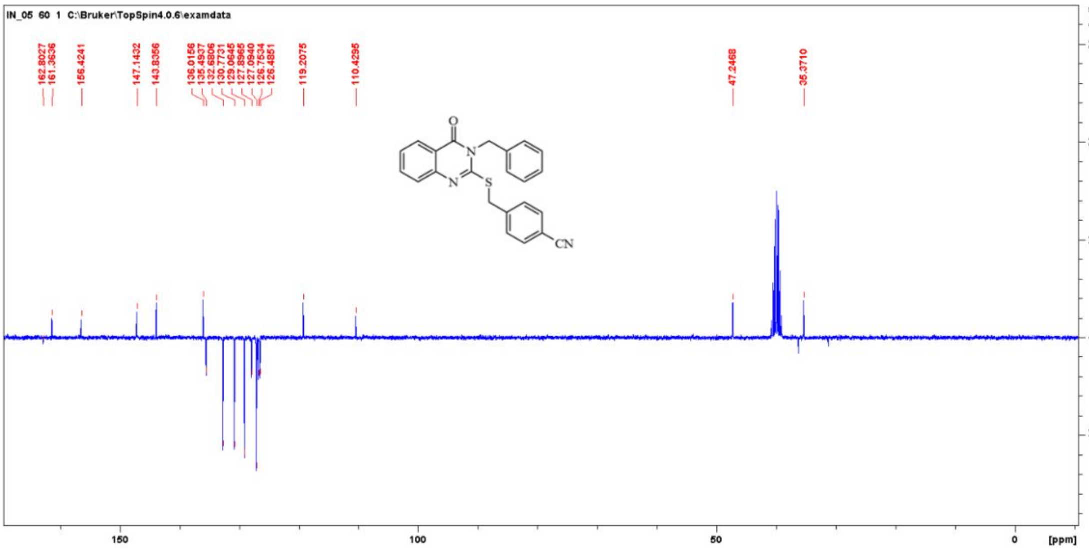

Fig. S12. ^{13}C-NMR for compound **1b**

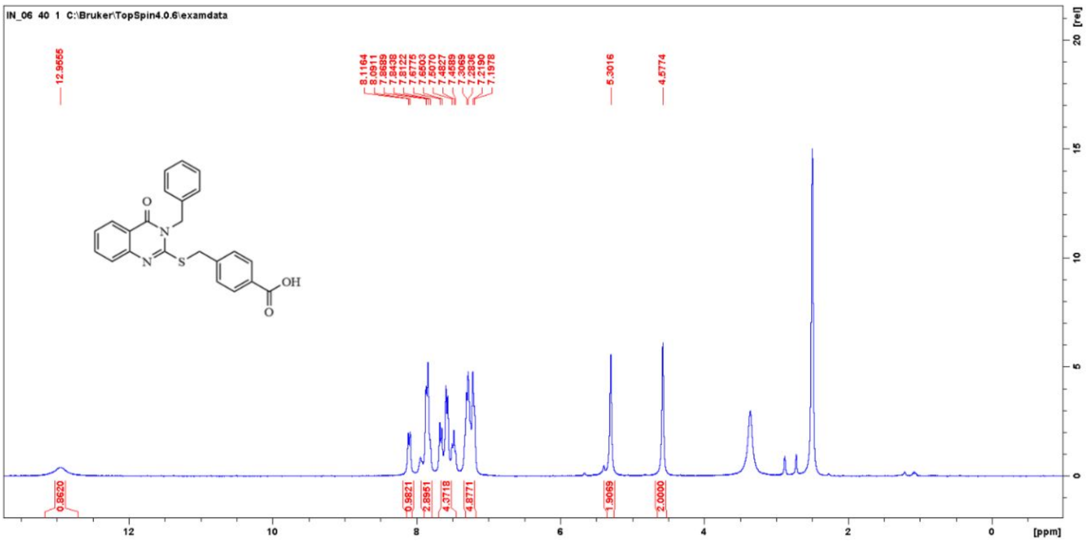

Fig. S13. ^{1}H-NMR for compound **1c**

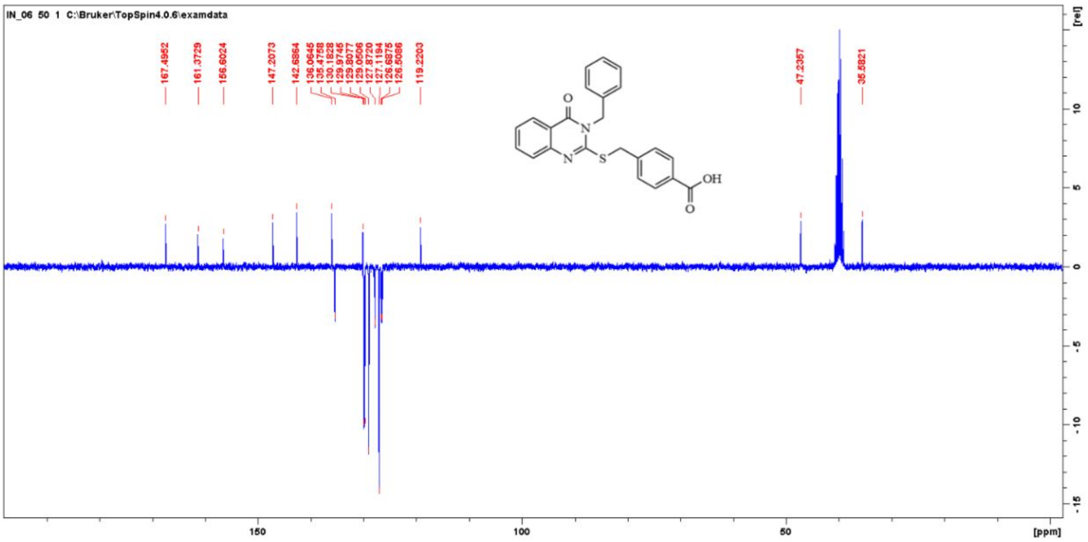

Fig. S14. ^{13}C-NMR for compound **1c**

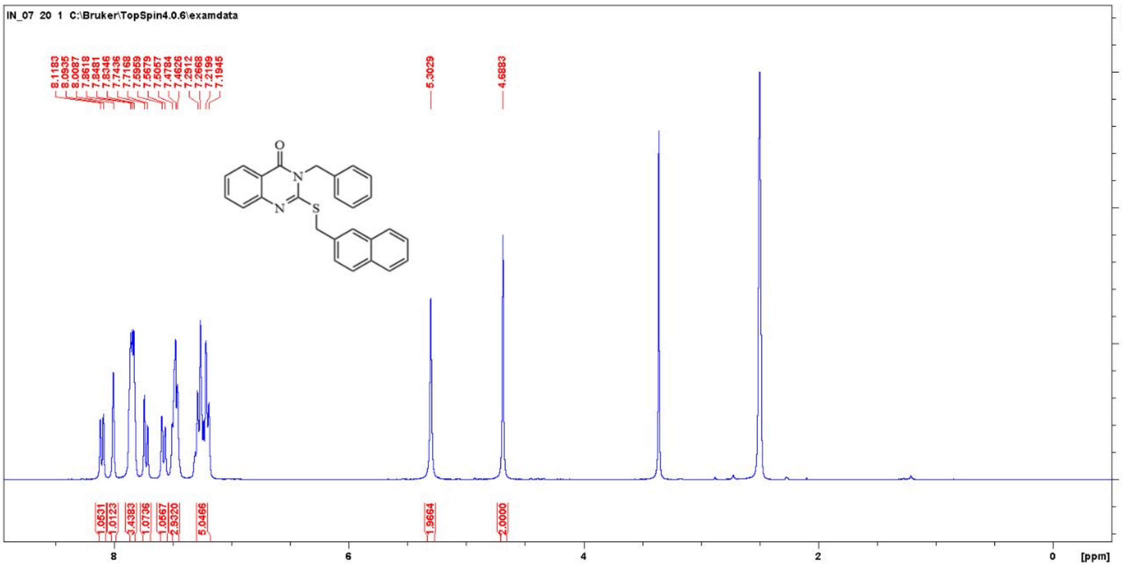

Fig. S15. ^{1}H-NMR for compound **1d**

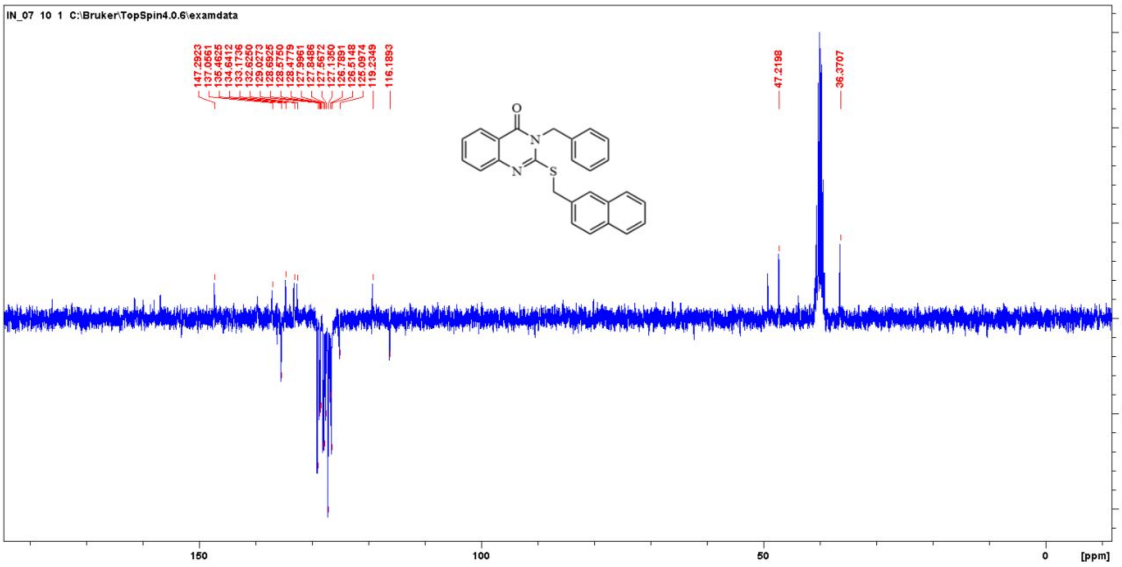

Fig. S16. ^{13}C-NMR for compound **1d**

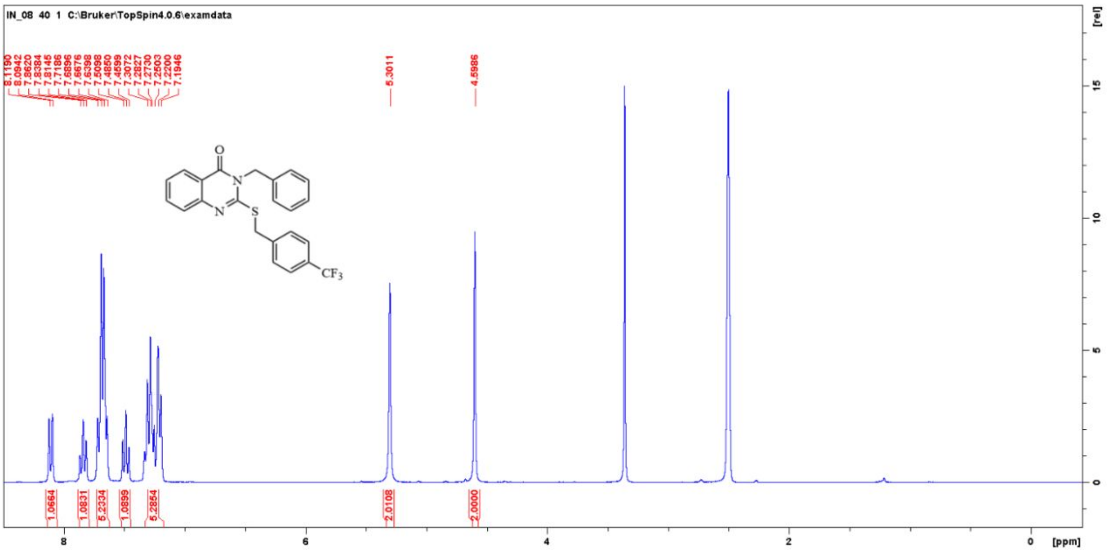

Fig. S17. ^{1}H-NMR for compound **1e**

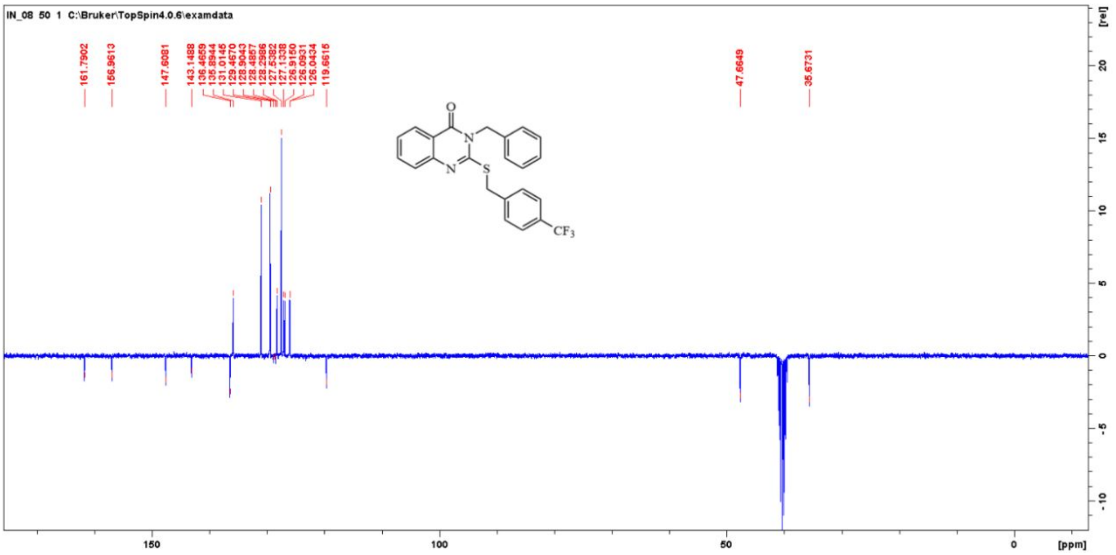

Fig. S18. ^{13}C-NMR for compound **1e**

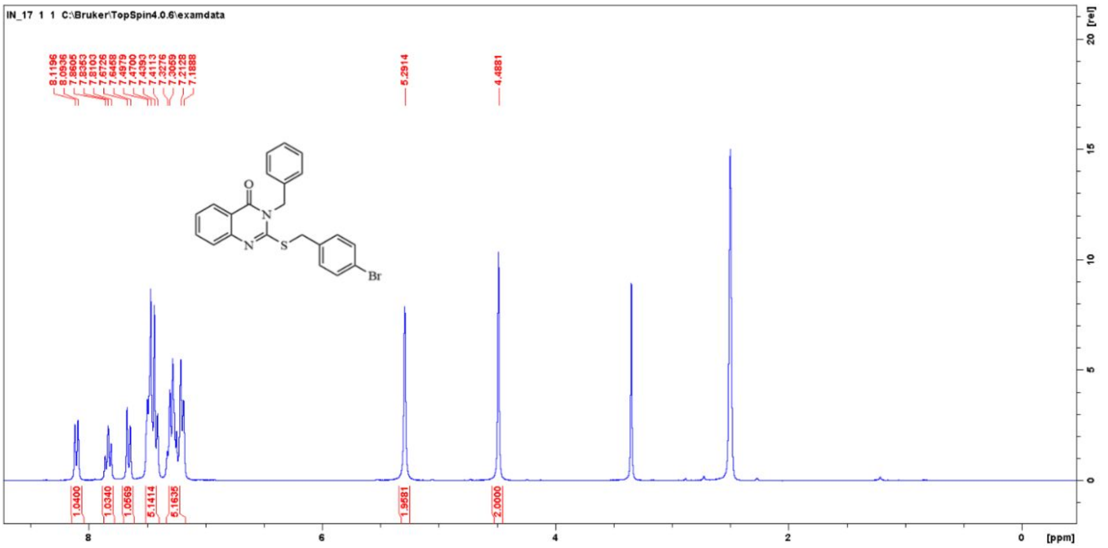

Fig. S19. ^{1}H-NMR for compound **1f**

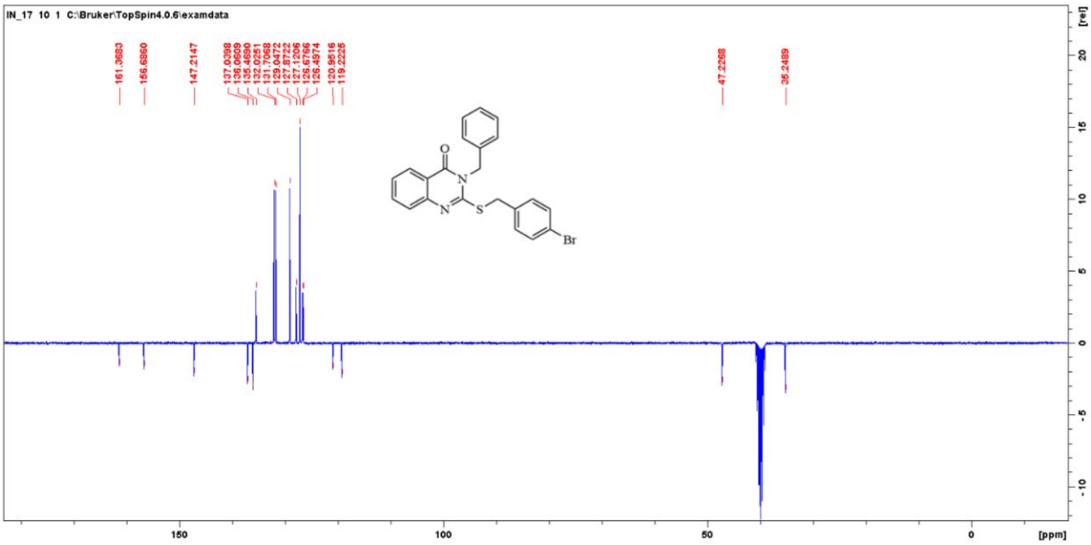

Fig. S20. ^{13}C-NMR for compound **1f**

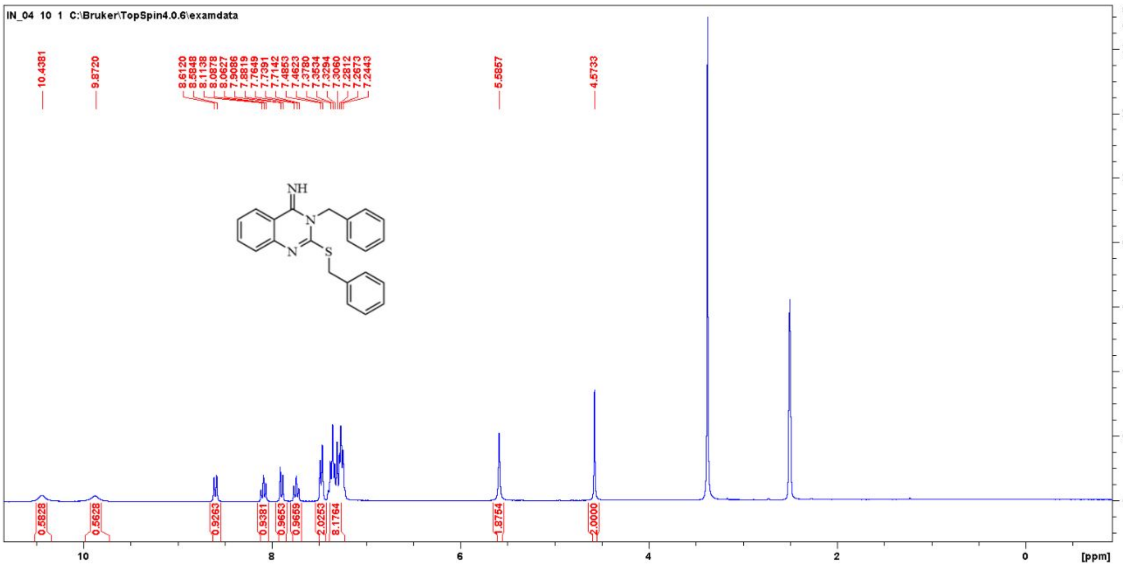

Fig. S21. ^{1}H-NMR for compound **2a**

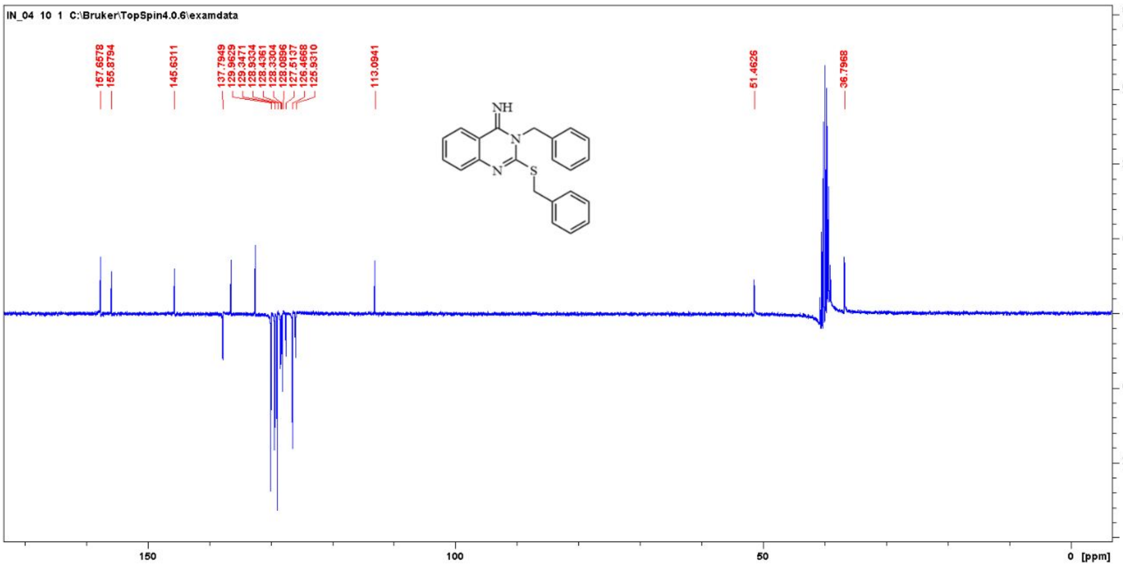

Fig. S22. ^{13}C-NMR for compound **2a**

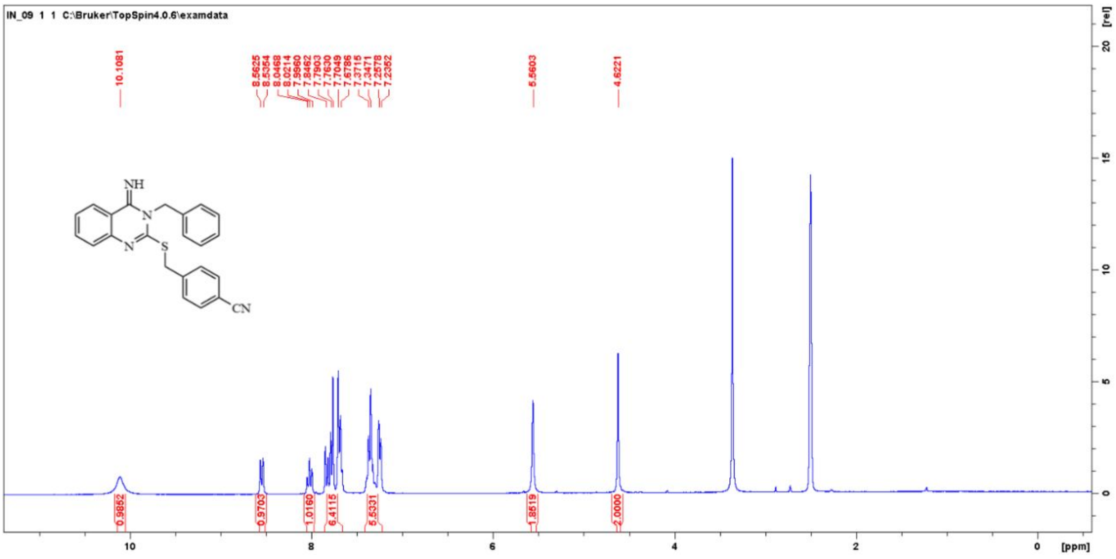

Fig. S23. ^{1}H-NMR for compound **2b**

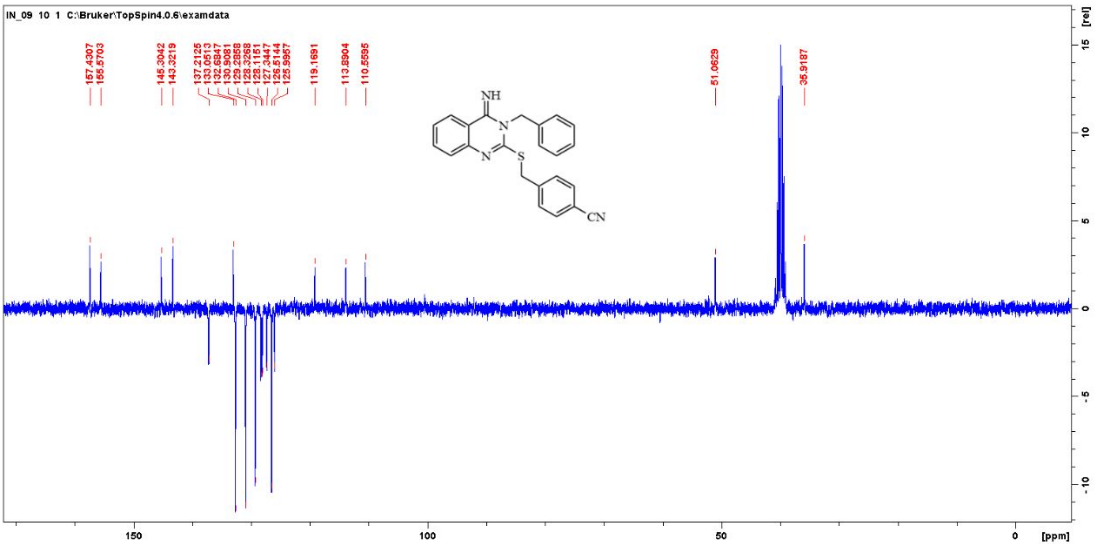

Fig. S24. ^{13}C-NMR for compound **2b**

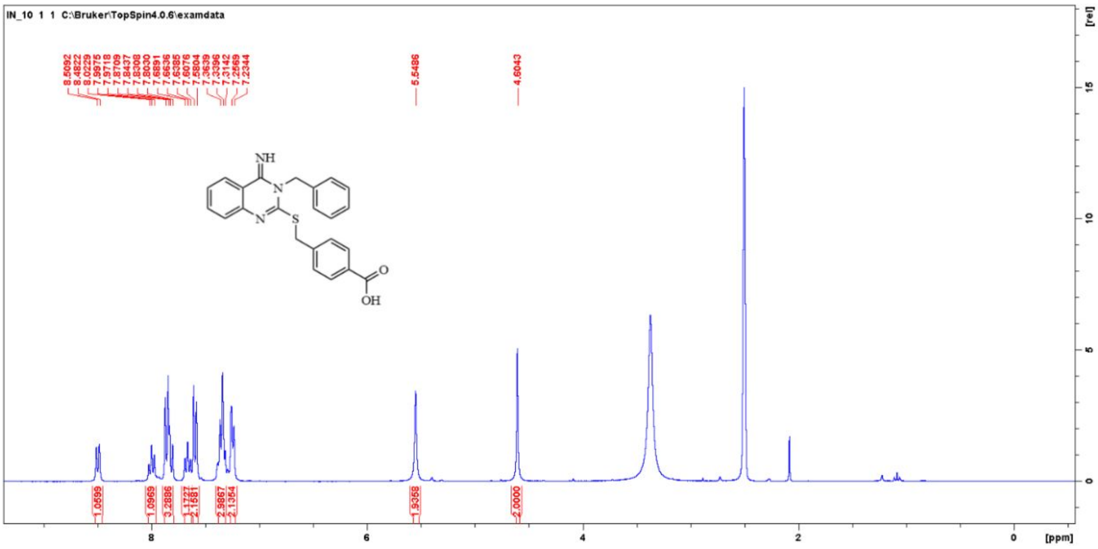

Fig. S25. ^{1}H-NMR for compound **2c**

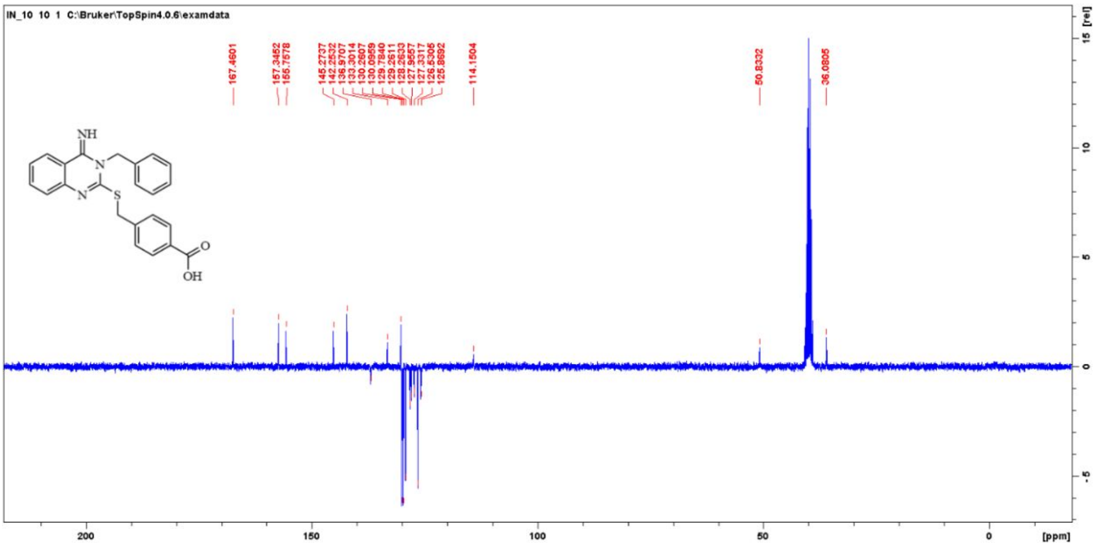

Fig. S26. ^{13}C-NMR for compound **2c**

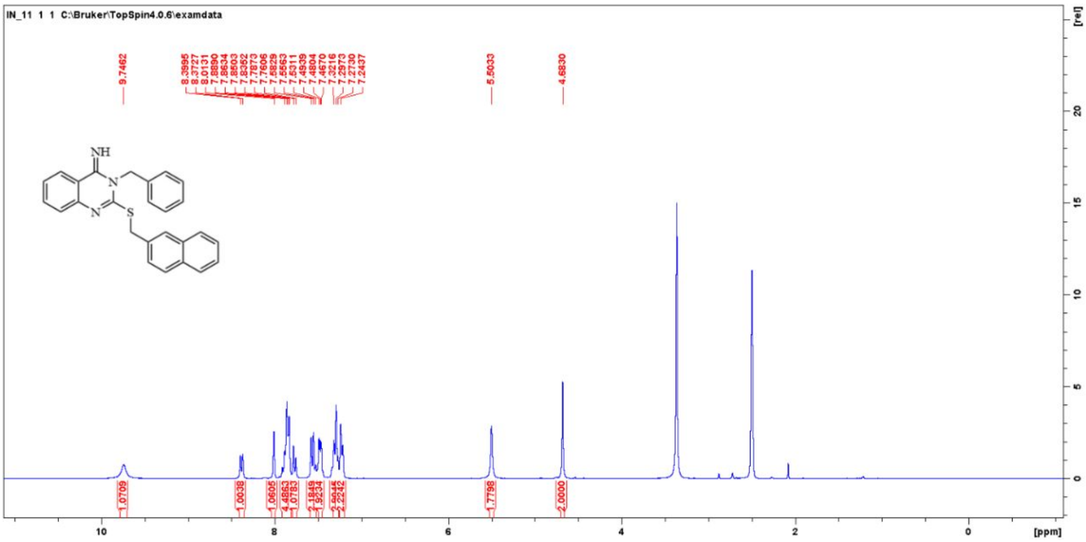

Fig. S27. ^{1}H-NMR for compound **2d**

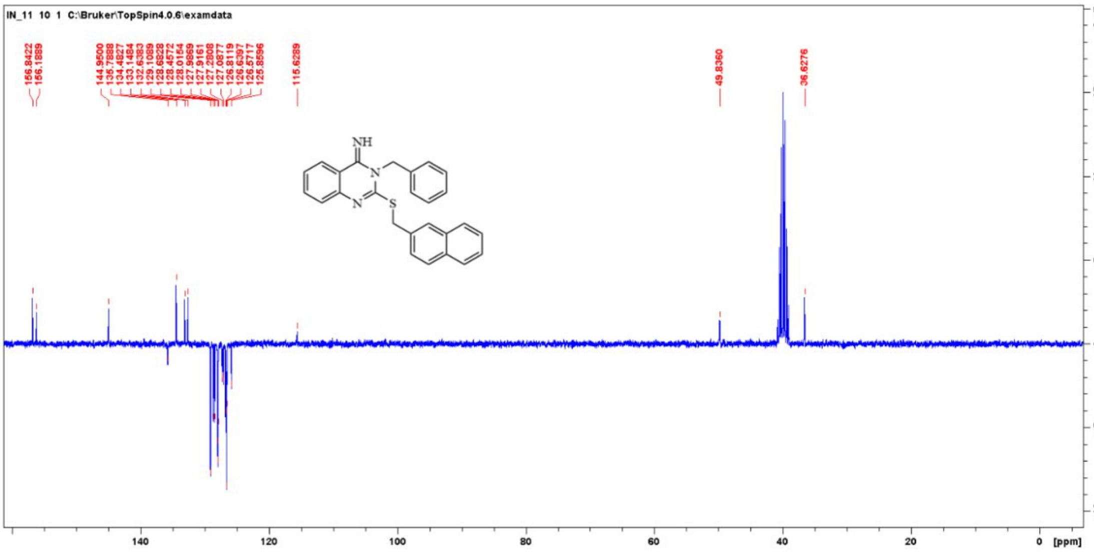

Fig. S28. ^{13}C-NMR for compound **2d**

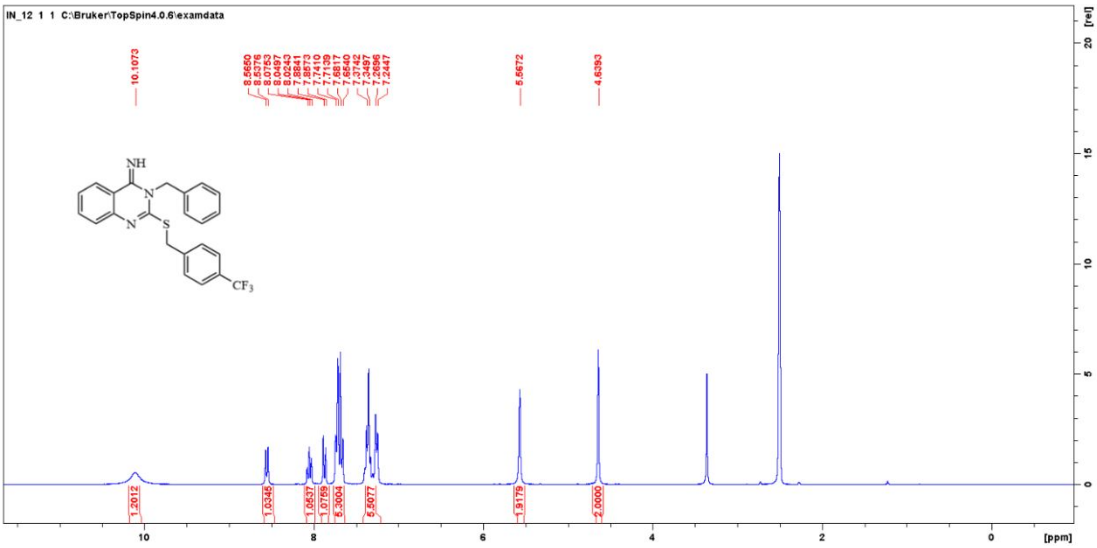

Fig. S29. ^{1}H-NMR for compound **2e**

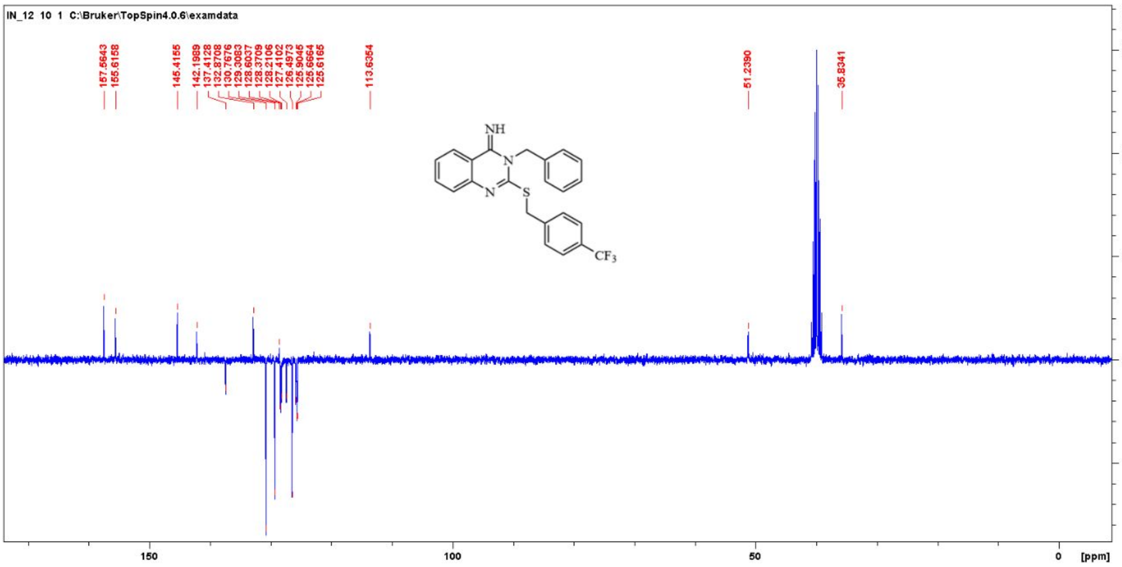

Fig. S24. ^{13}C-NMR for compound **2e**

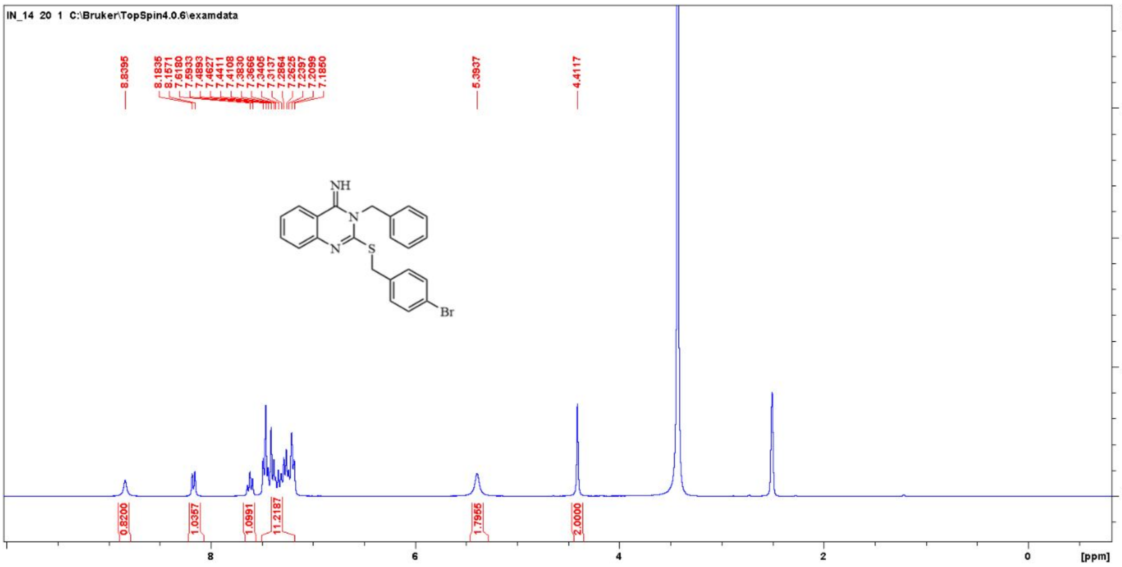

Fig. S25. ^{1}H-NMR for compound **2f**

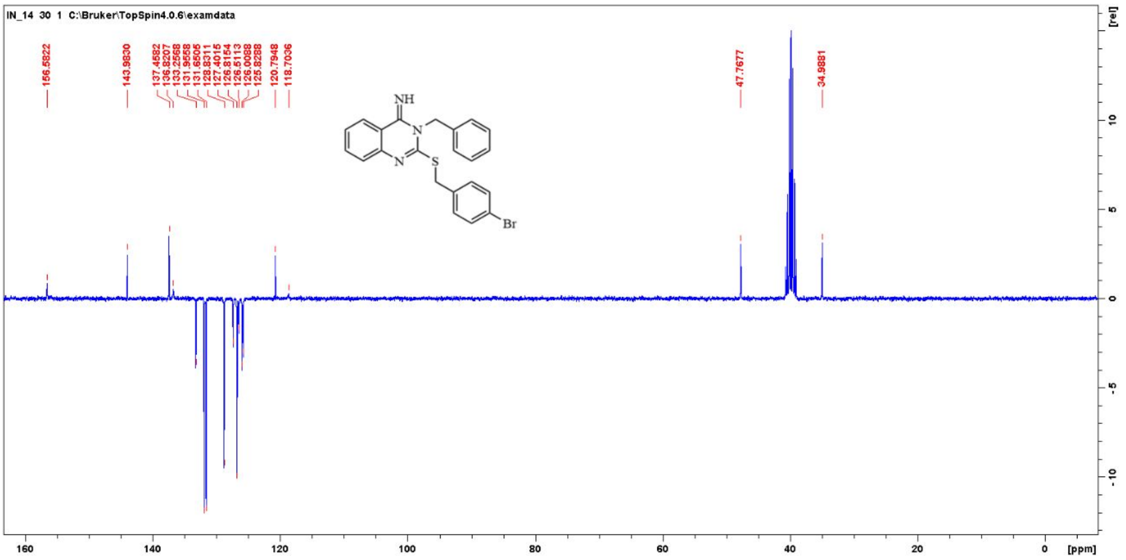

Fig. S26. ^{13}C-NMR for compound **2f**

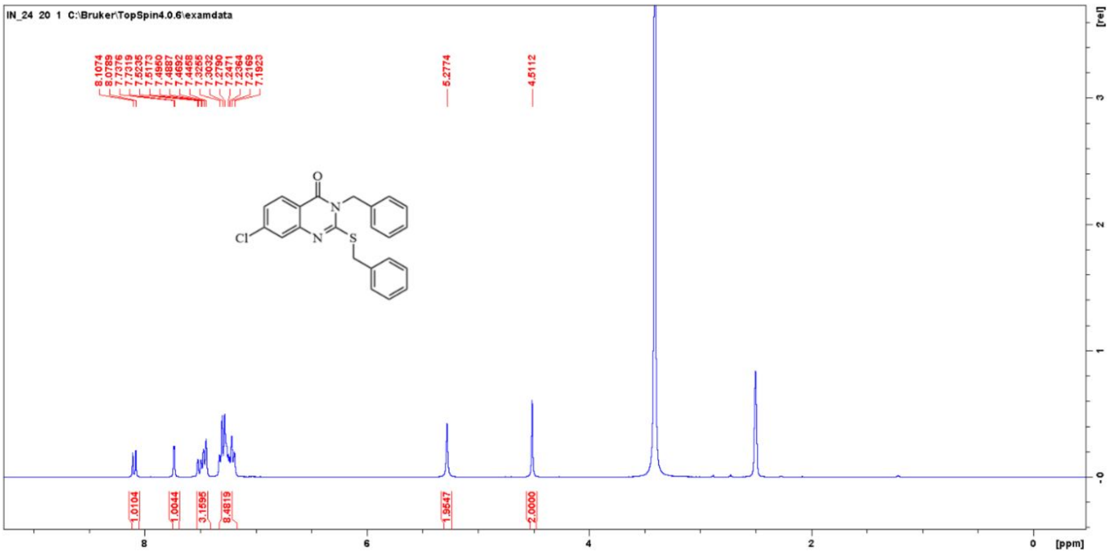

Fig. S27. ^{1}H-NMR for compound **3a**

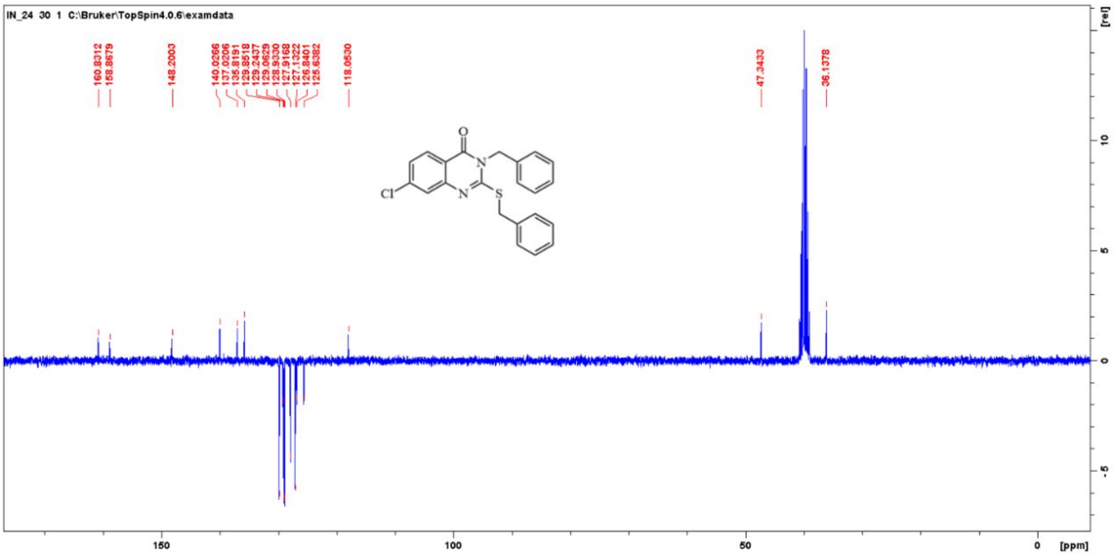

Fig. S28. ^{13}C-NMR for compound **3a**

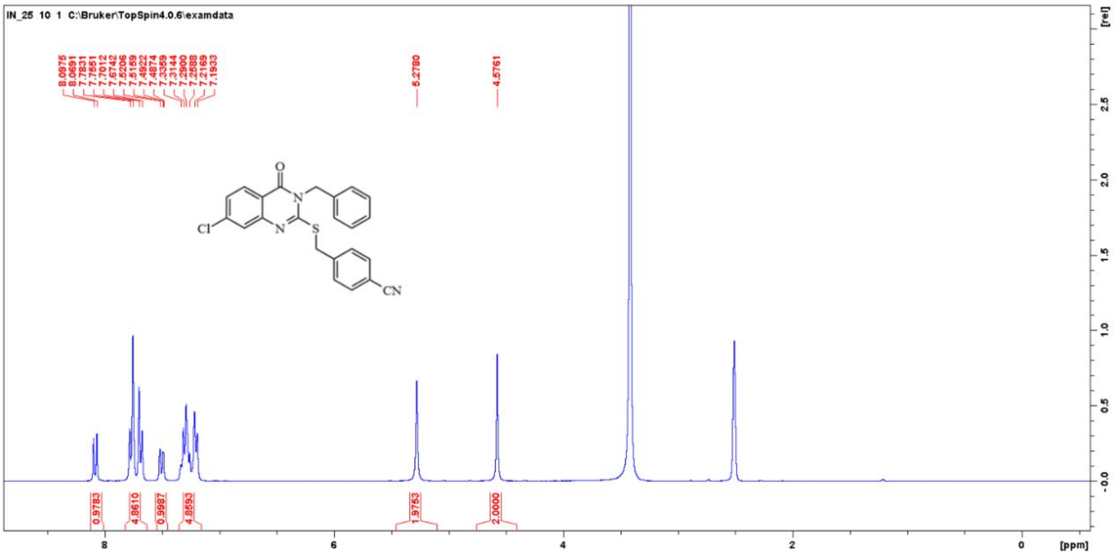

Fig. S29. ^{1}H-NMR for compound **3b**

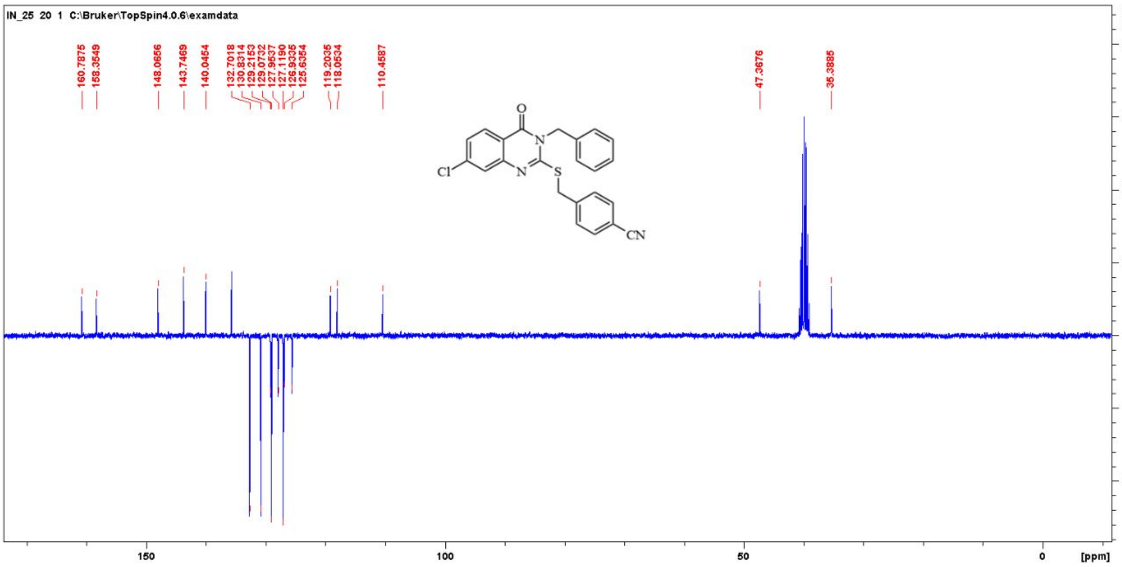

Fig. S30. ^{13}C-NMR for compound **3b**

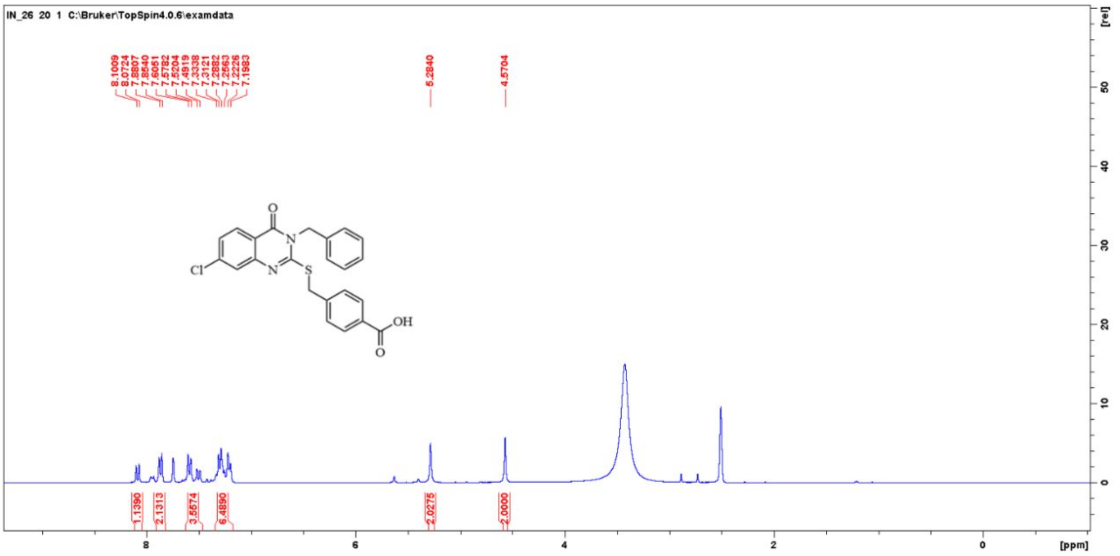

Fig. S31. ^{1}H-NMR for compound **3c**

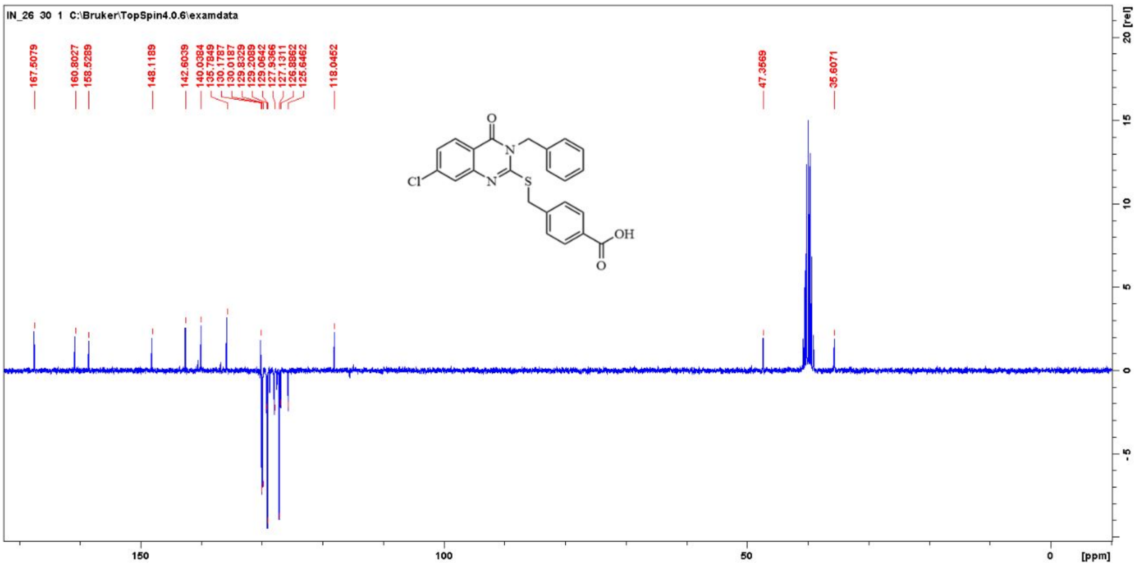

Fig. S32. ^{13}C-NMR for compound **3c**

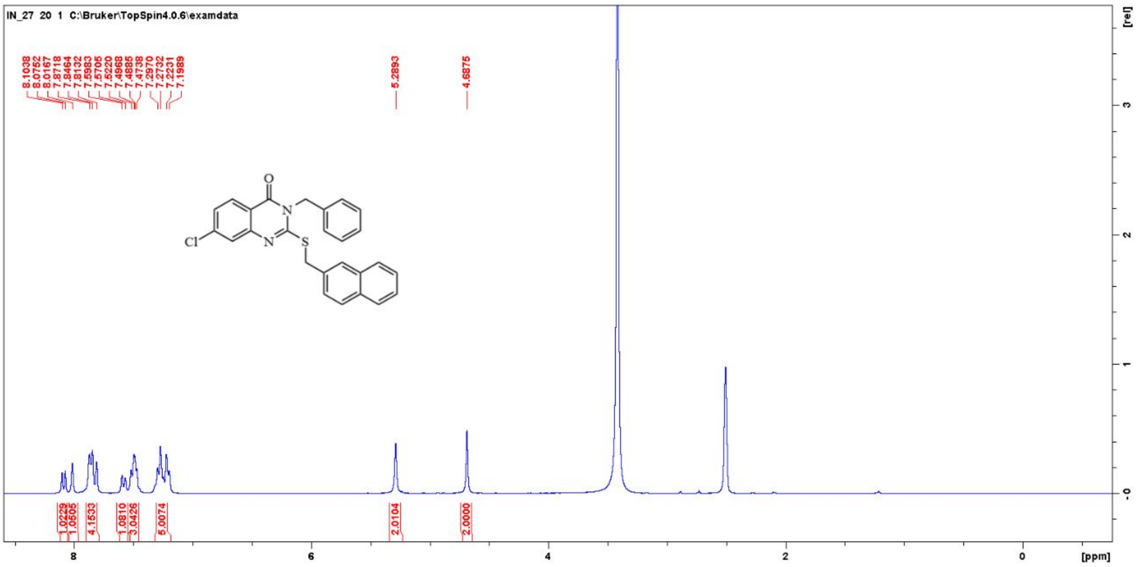

Fig. S33. ^{1}H-NMR for compound **3d**

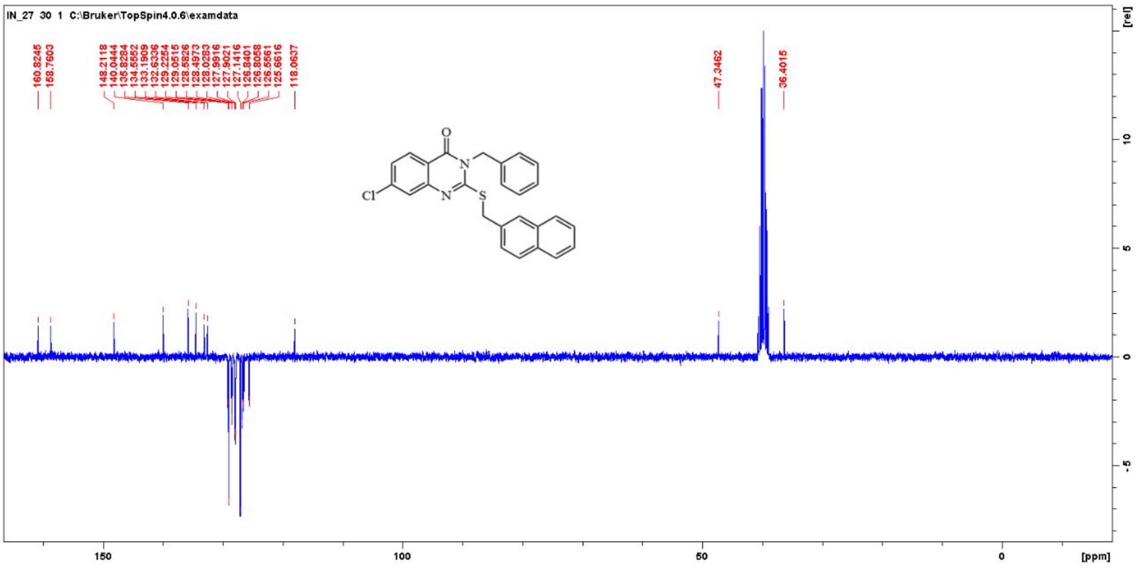

Fig. S34. ^{13}C-NMR for compound **3d**

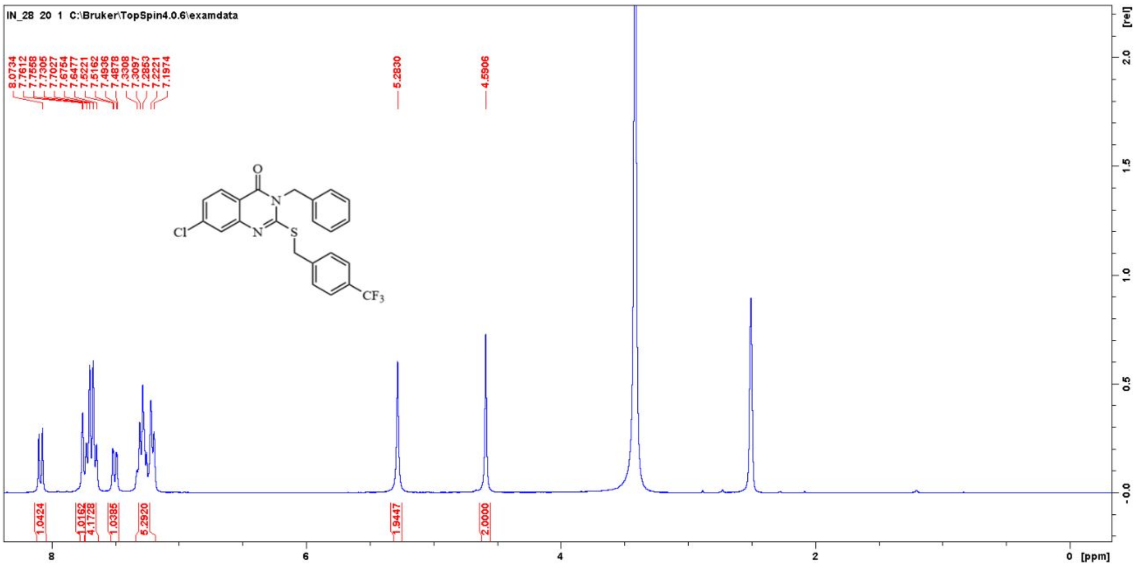

Fig. S35. ^{1}H-NMR for compound **3e**

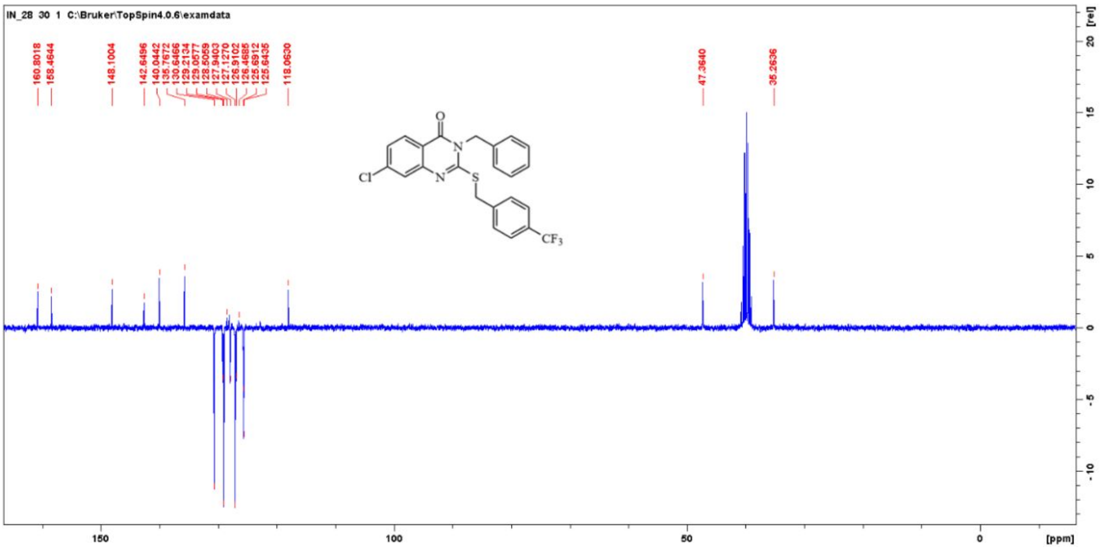

Fig. S36. ^{13}C-NMR for compound **3e**

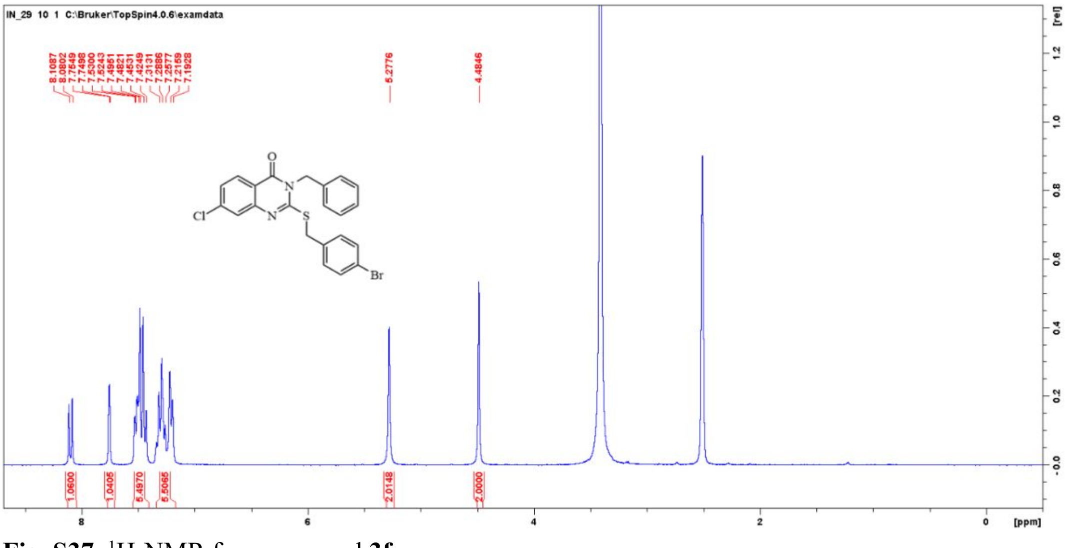

Fig. S37. ^{1}H-NMR for compound **3f**

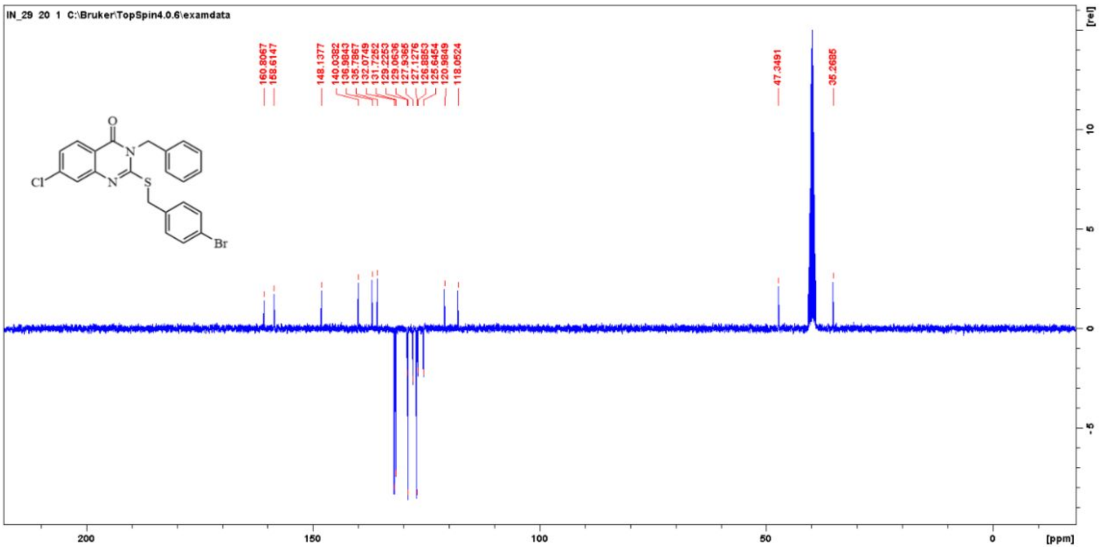

Fig. S38. ^{13}C-NMR for compound **3f**

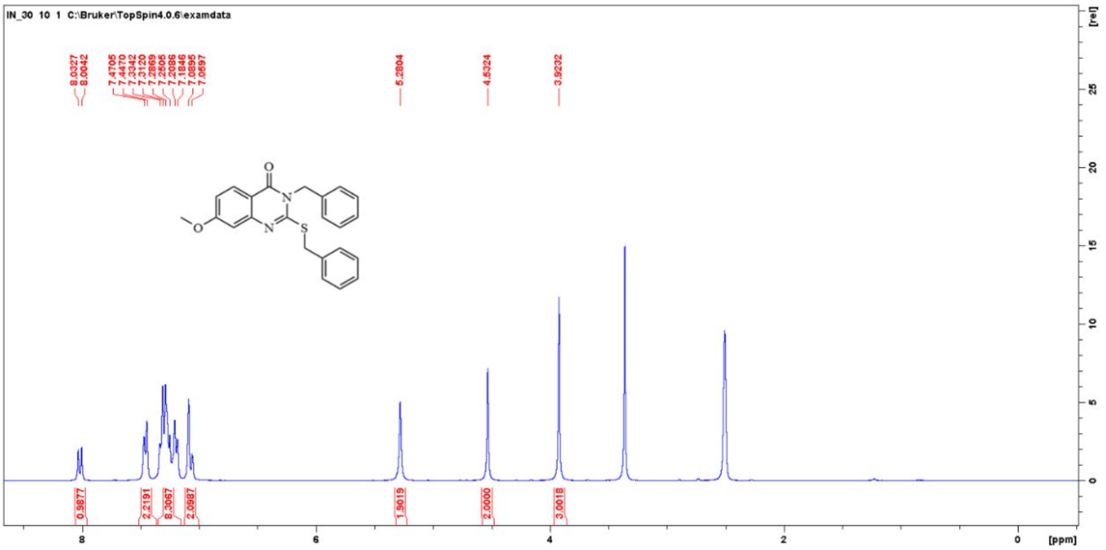

Fig. S39. ^{1}H-NMR for compound **4a**

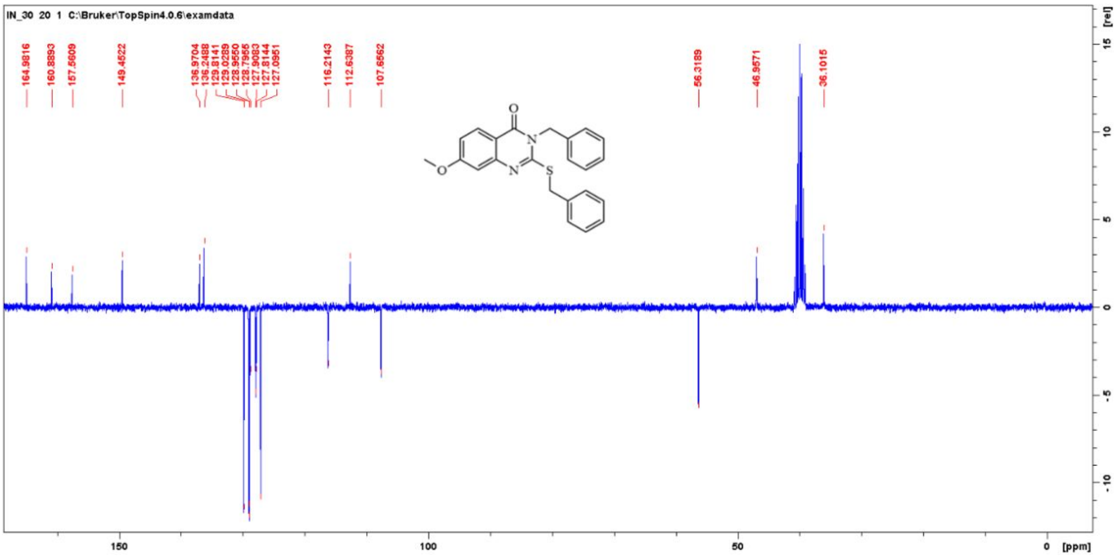

Fig. S40. ^{13}C-NMR for compound **4a**

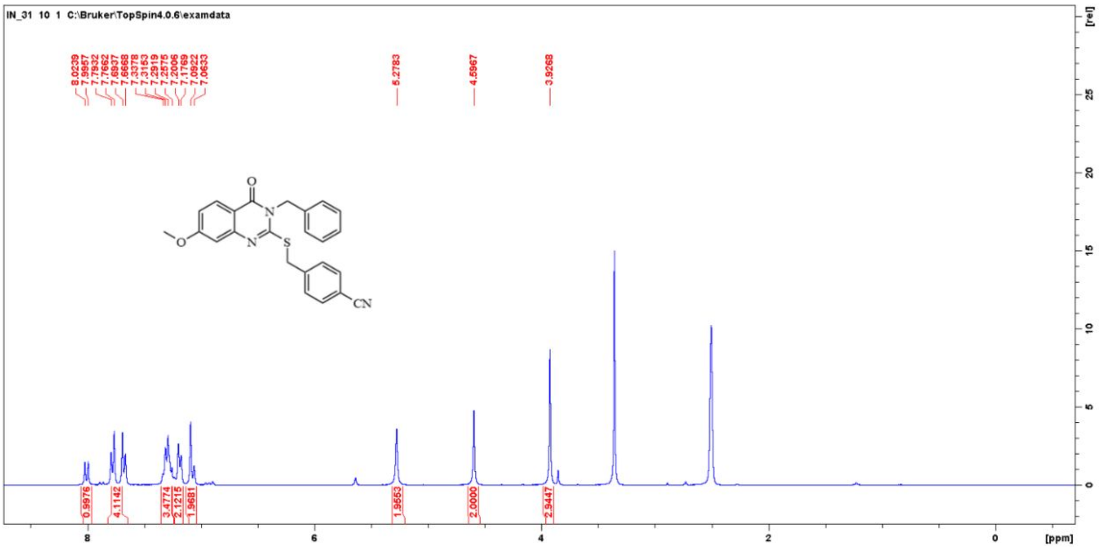

Fig. S41. ^{1}H-NMR for compound **4b**

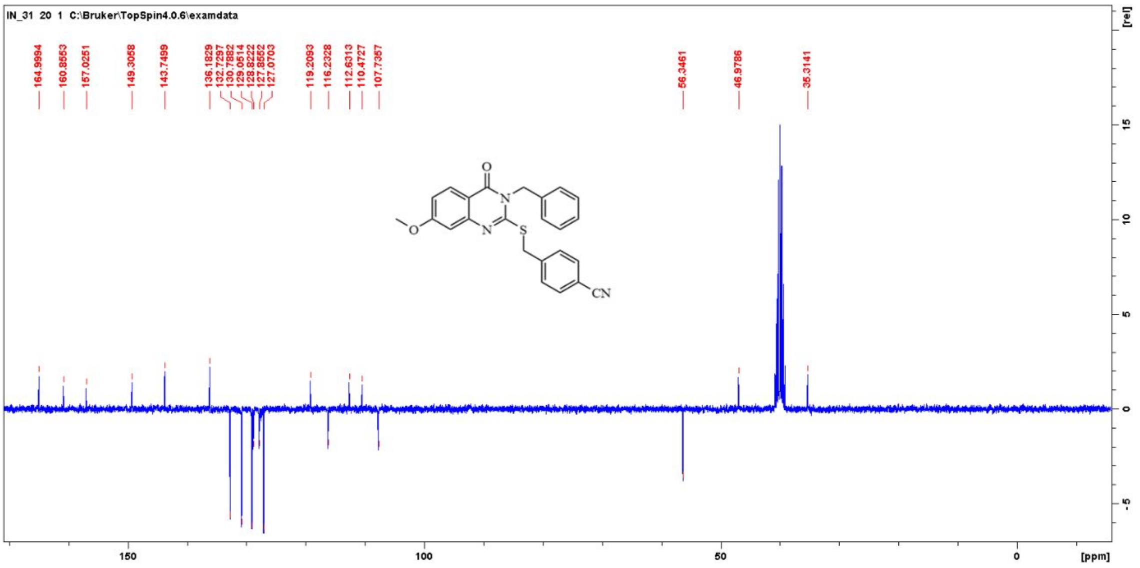

Fig. S42. ^{13}C-NMR for compound **4b**

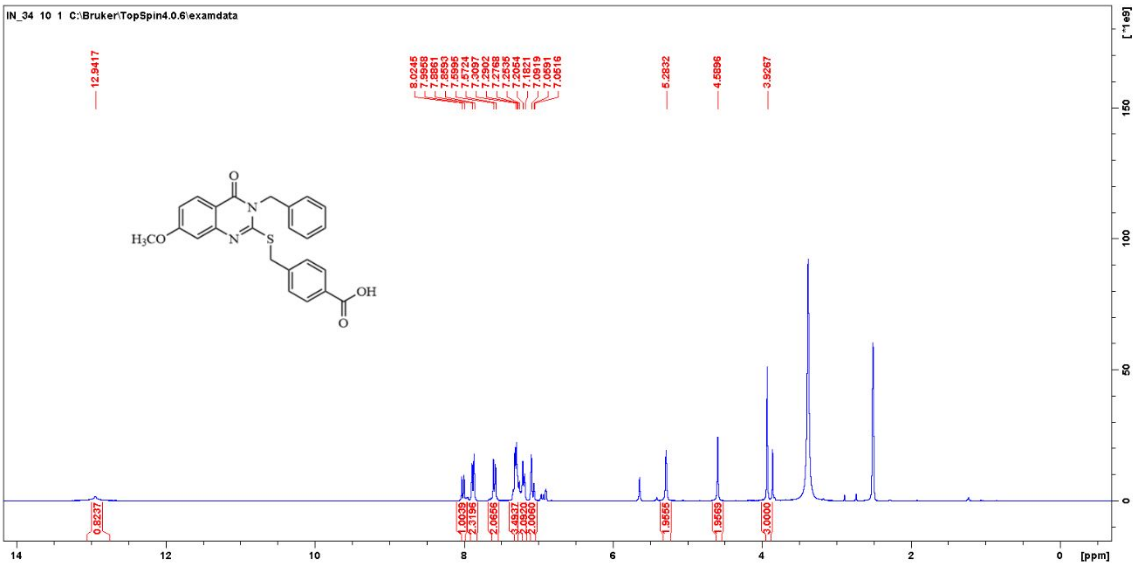

Fig. S43. ^{1}H-NMR for compound **4c**

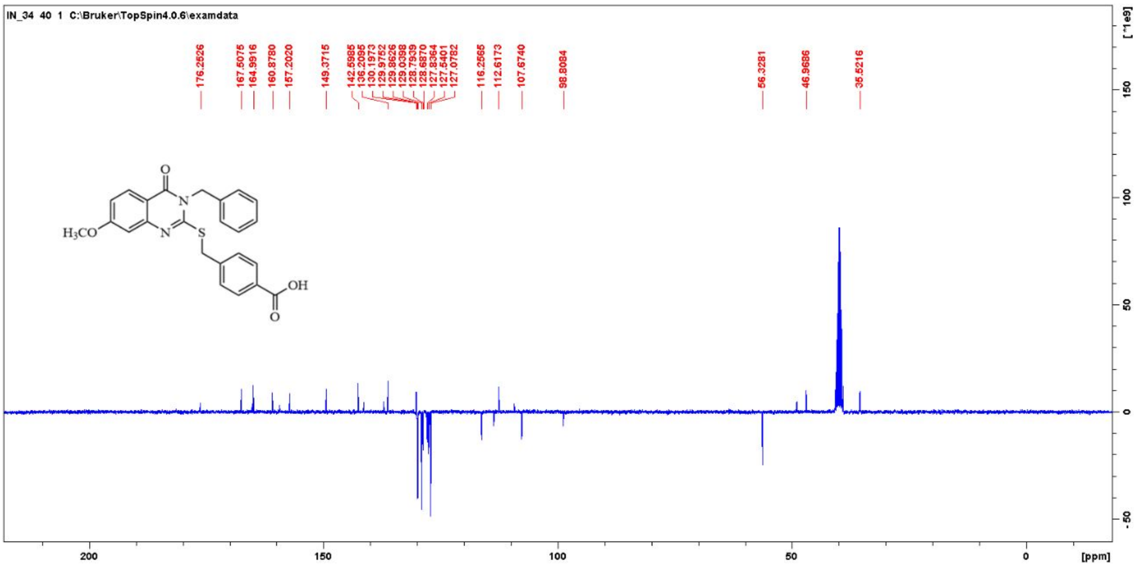

Fig. S44. ^{13}C-NMR for compound **4c**

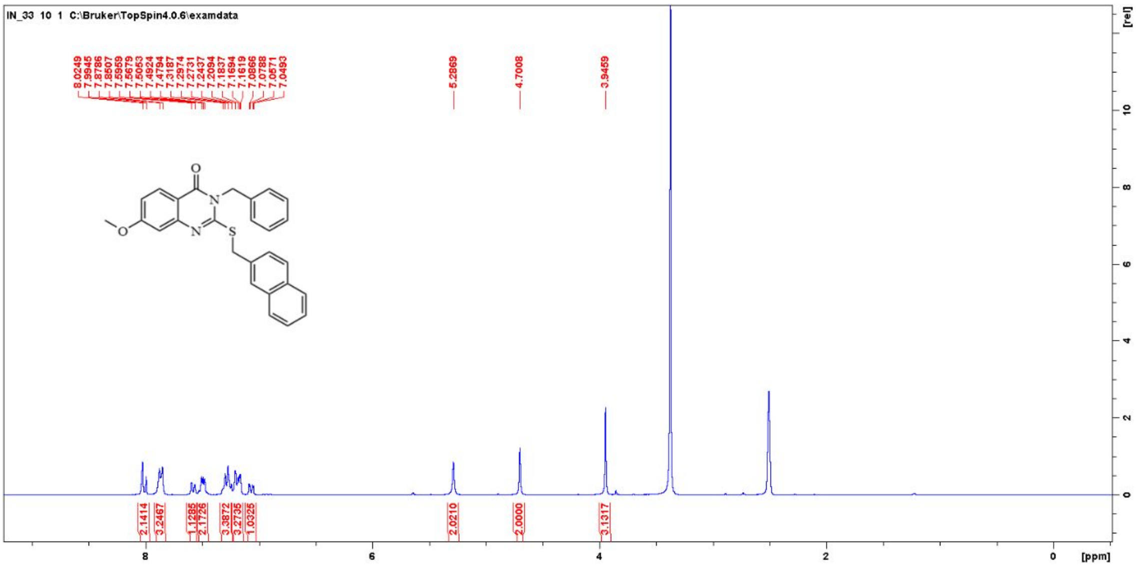

Fig. S45. ^{1}H-NMR for compound **4d**

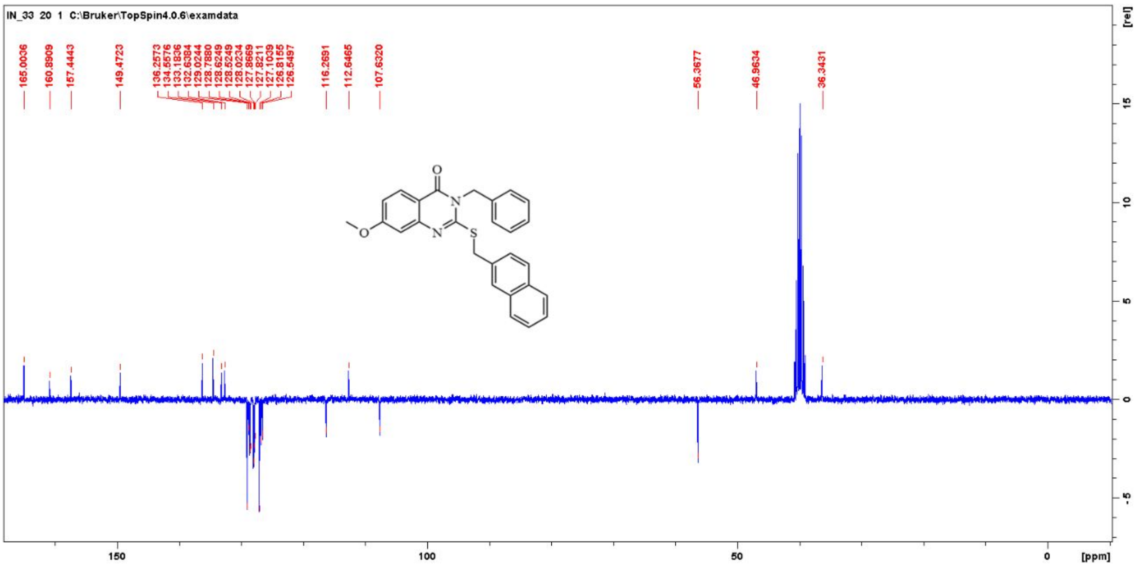

Fig. S46. ^{13}C-NMR for compound **4d**

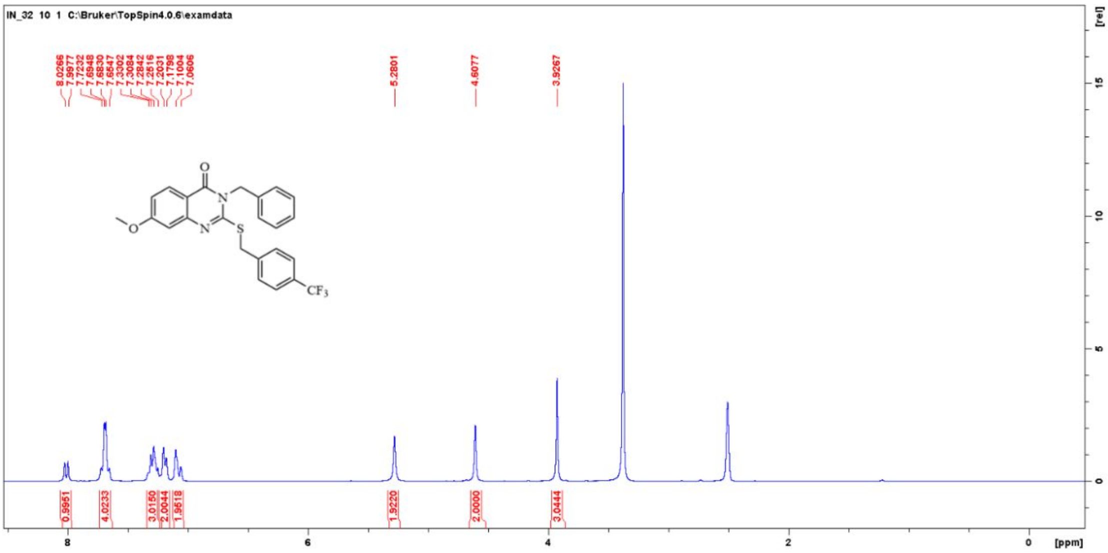

Fig. S47. ^{1}H-NMR for compound **4e**

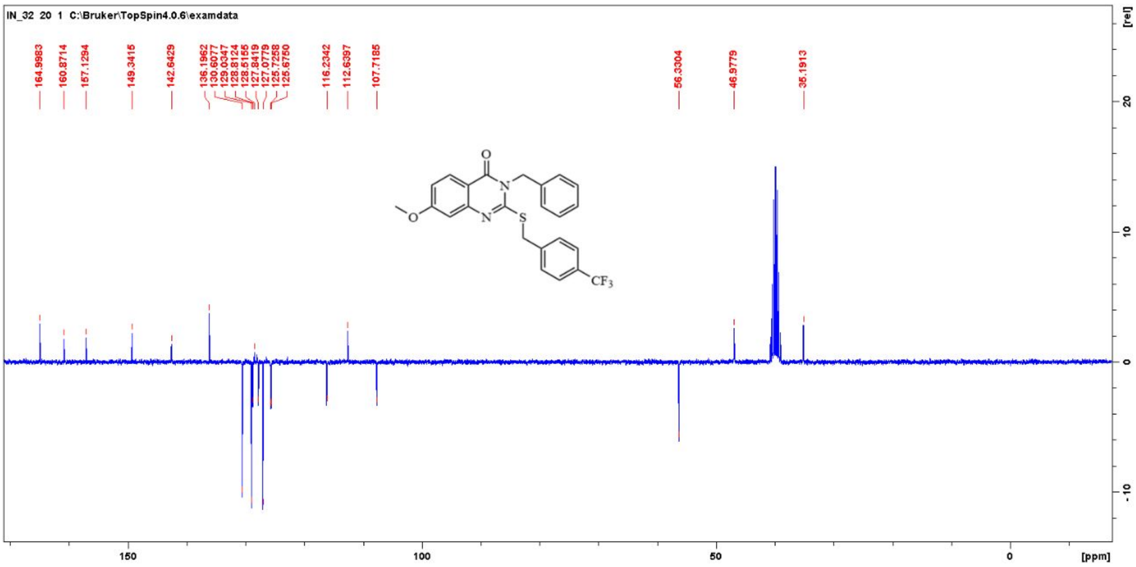

Fig. S48. ^{13}C-NMR for compound **4e**

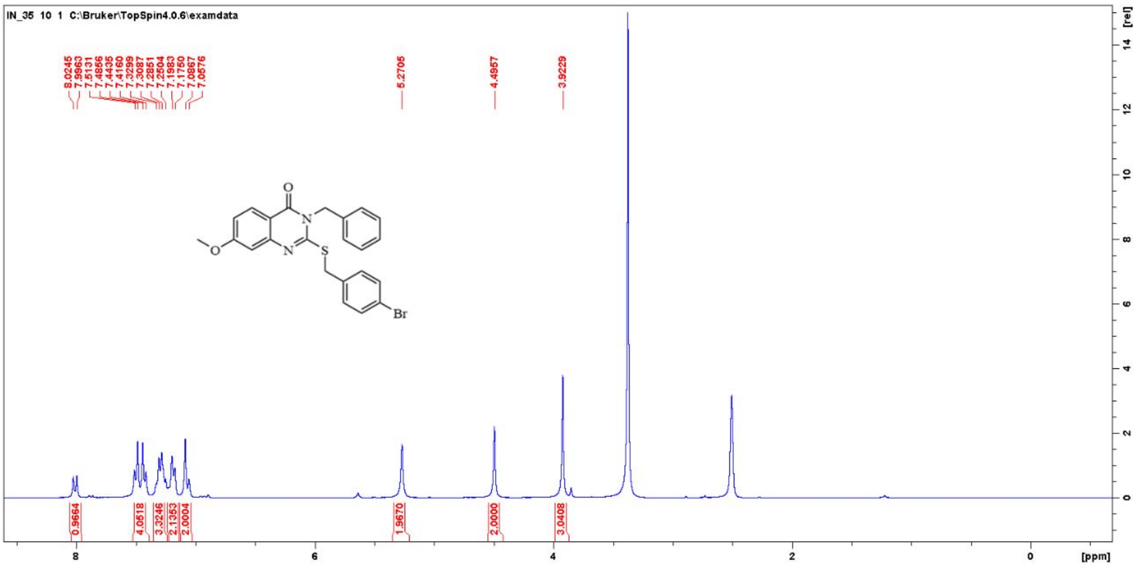

Fig. S49. ^{1}H-NMR for compound **4f**

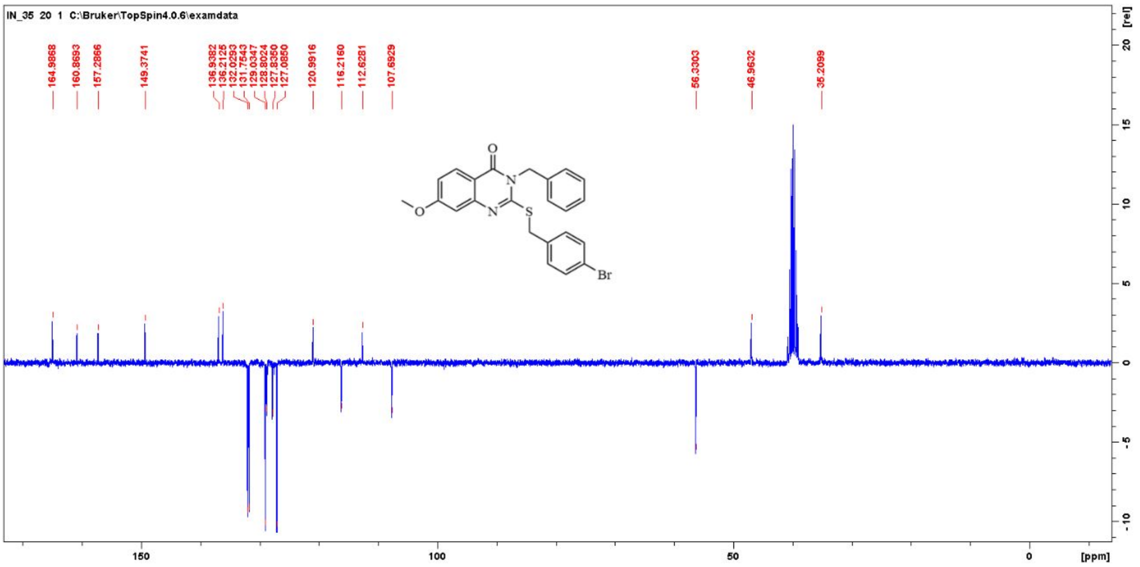

Fig. S50. ^{13}C-NMR for compound **4f**

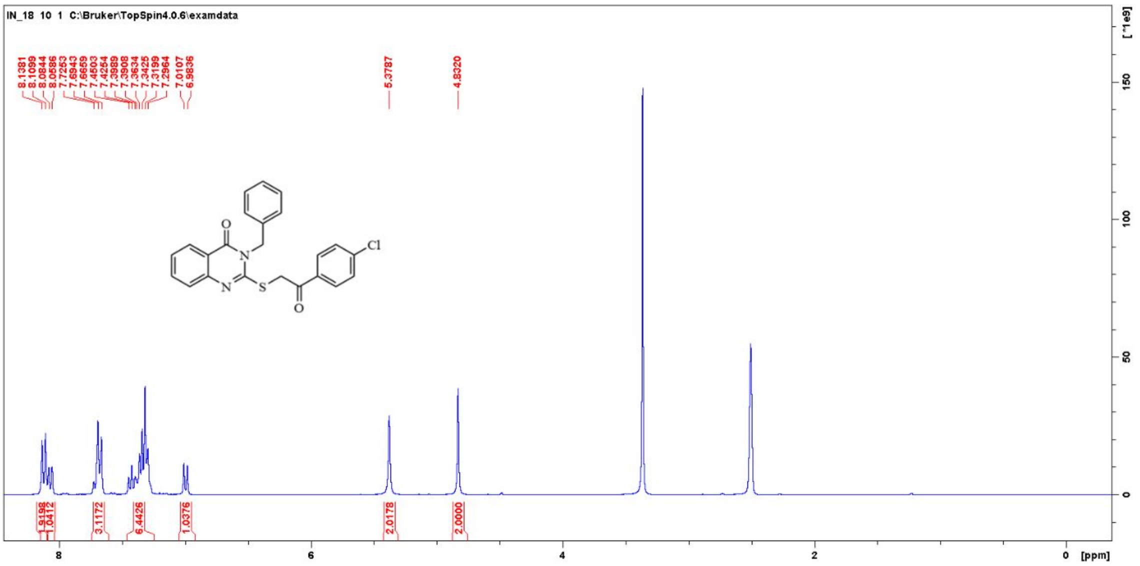

Fig. S51. ^{1}H-NMR for compound **5a**

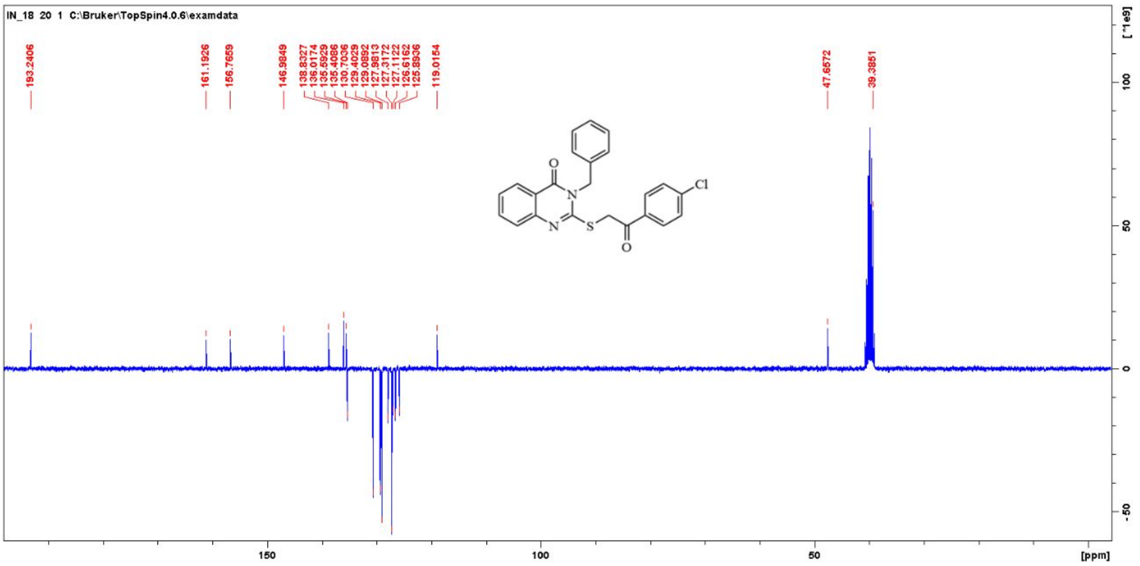

Fig. S52. ^{13}C-NMR for compound **5a**

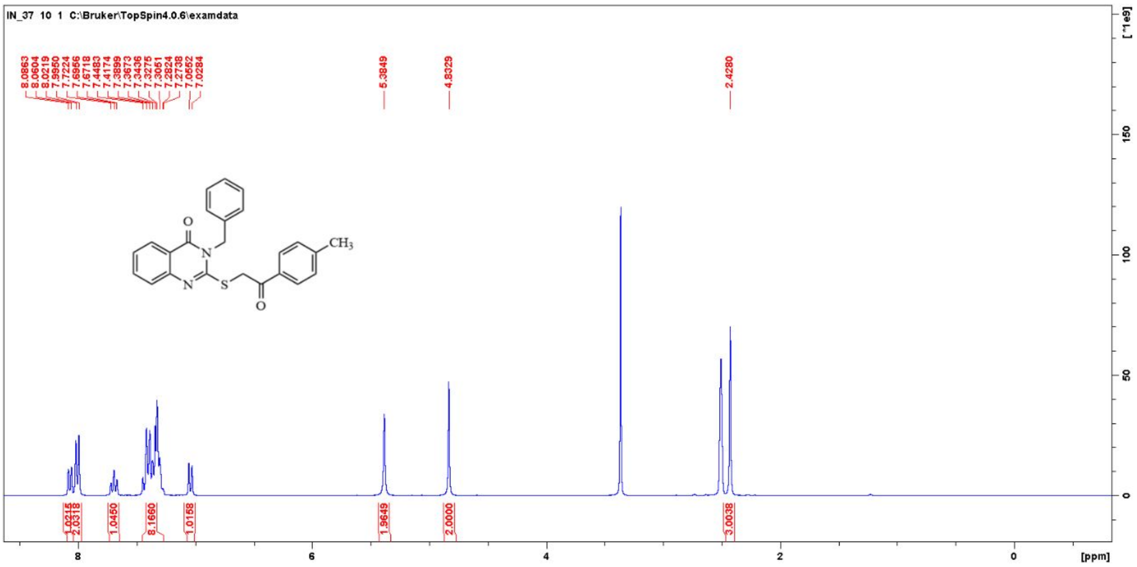

Fig. S53. ^{1}H-NMR for compound **5b**

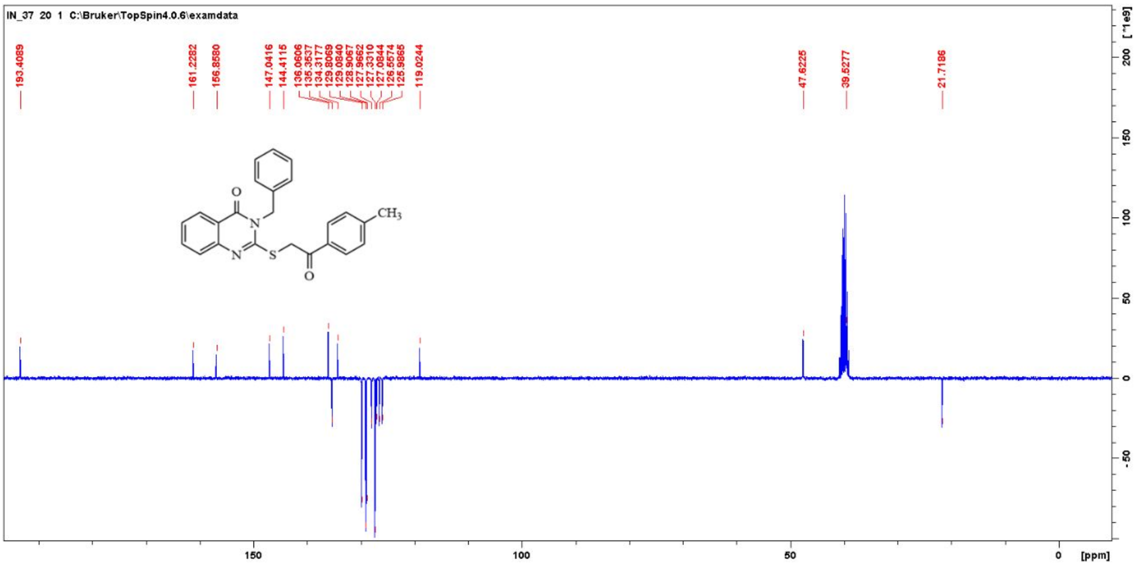

Fig. S54. ^{13}C-NMR for compound **5b**

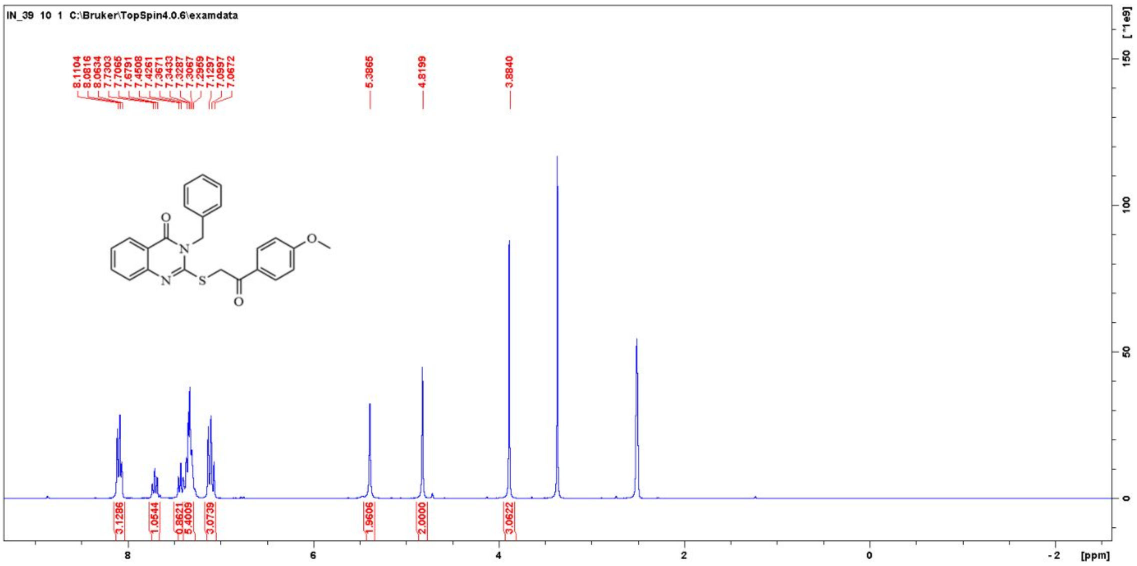

Fig. S55. ^{1}H-NMR for compound **5c**

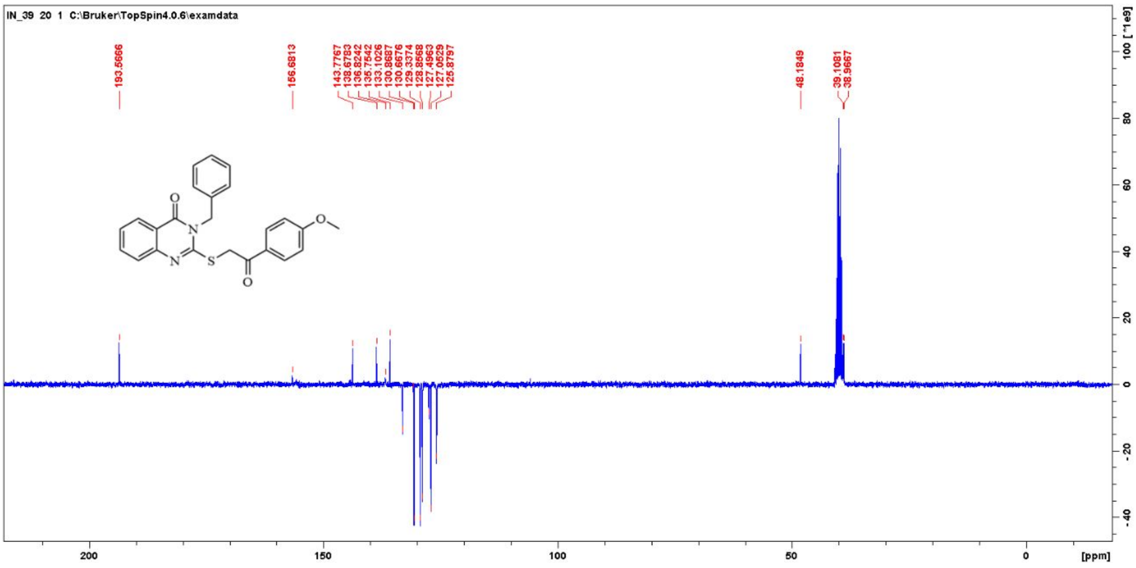

Fig. S56. ^{13}C-NMR for compound **5c**

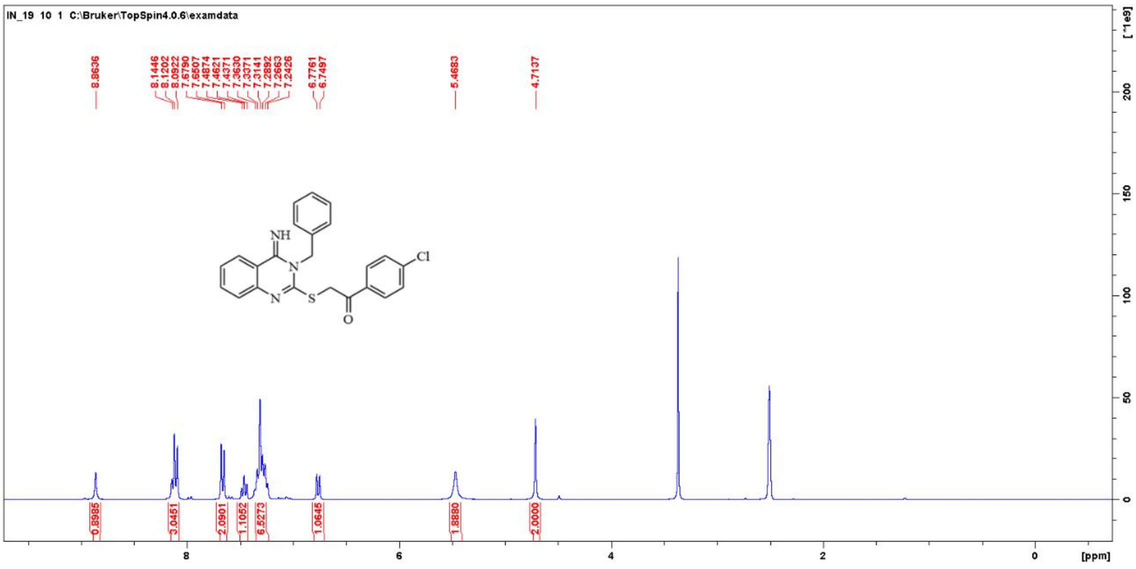

Fig. S57. ^{1}H-NMR for compound **6a**

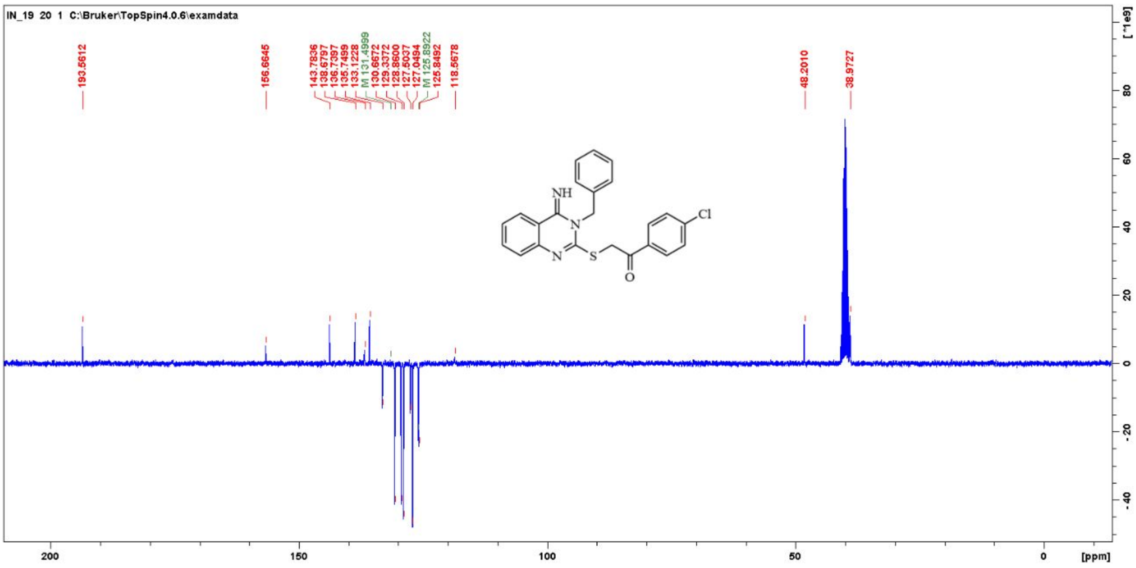

Fig. S58. ^{13}C-NMR for compound **6a**

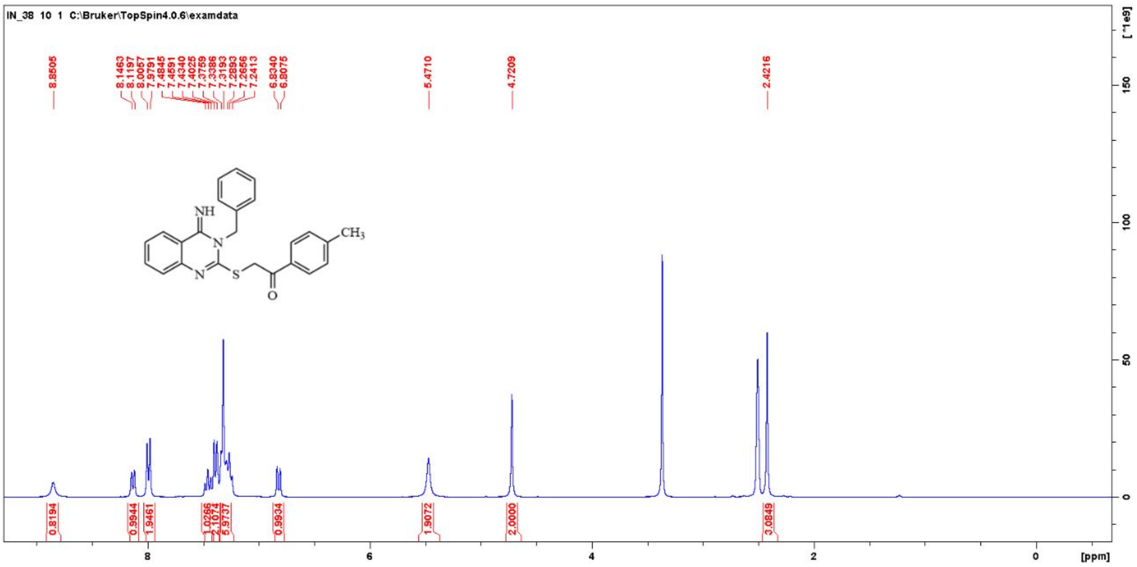

Fig. S59. ^{1}H-NMR for compound **6b**

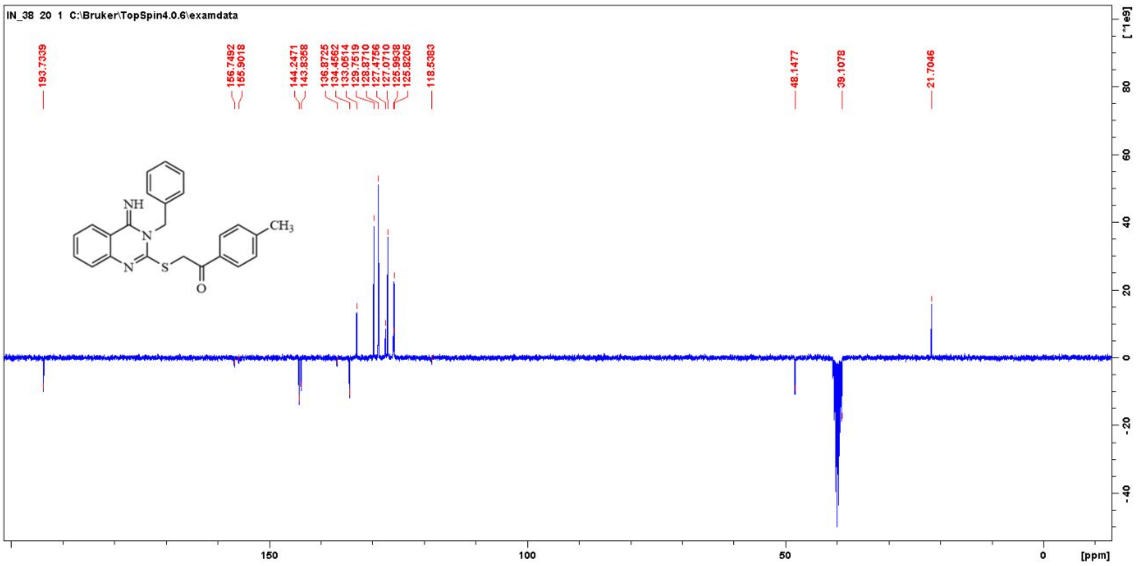

Fig. S60. ^{13}C-NMR for compound **6b**

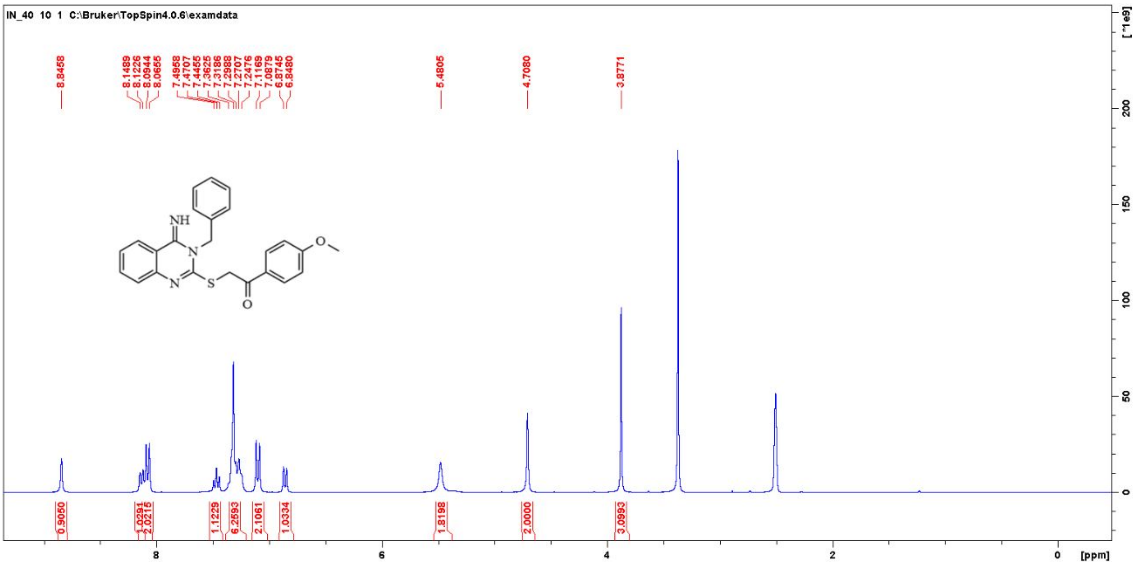

Fig. S61. ^{1}H-NMR for compound **6c**

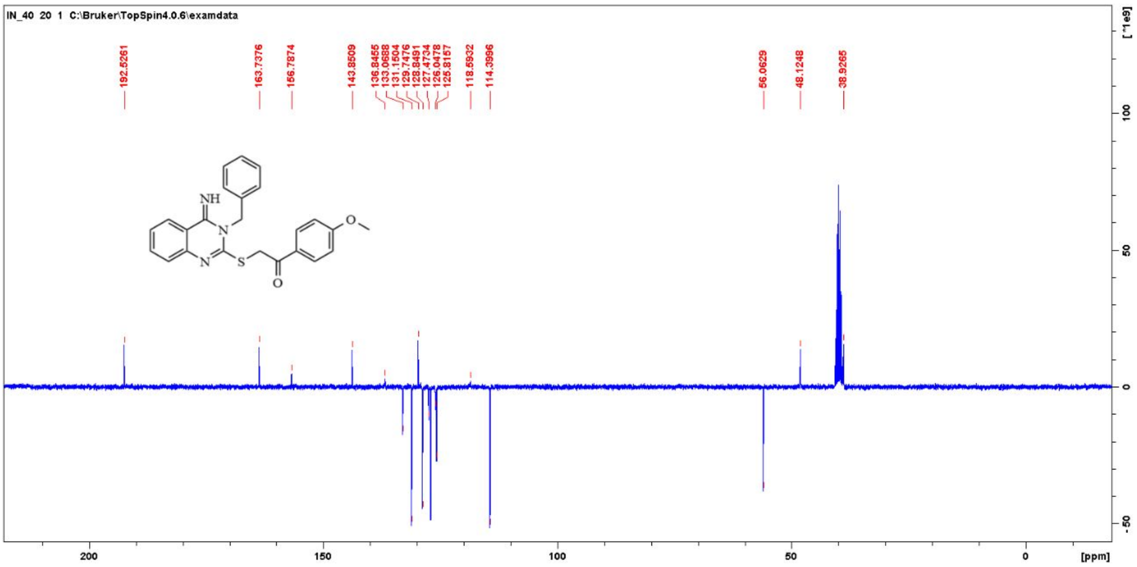

Fig. S62. ^{13}C-NMR for compound **6c**

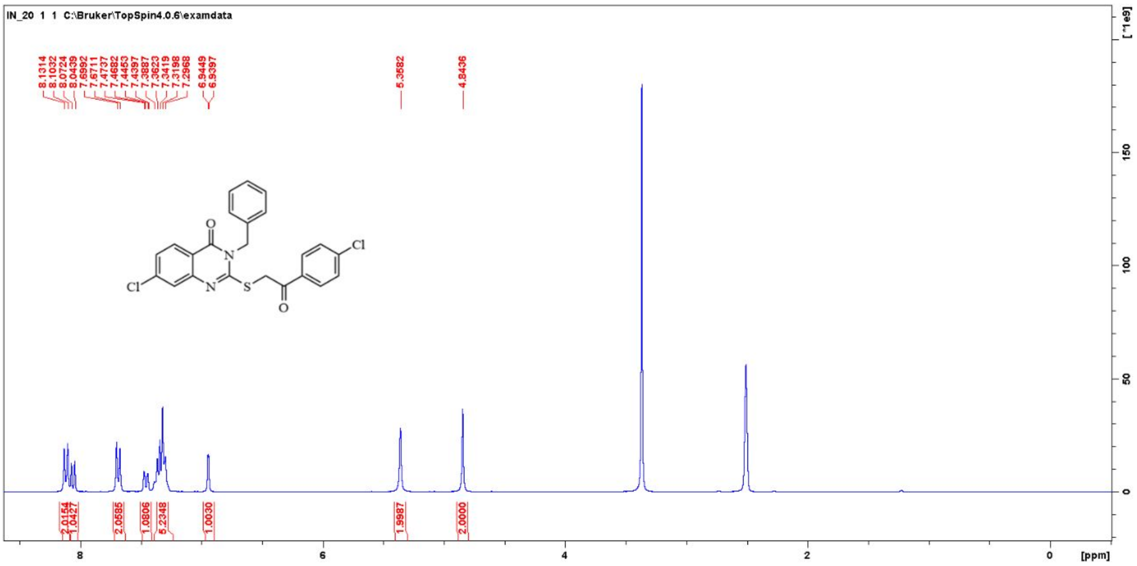

Fig. S63. ^{1}H-NMR for compound **7a**

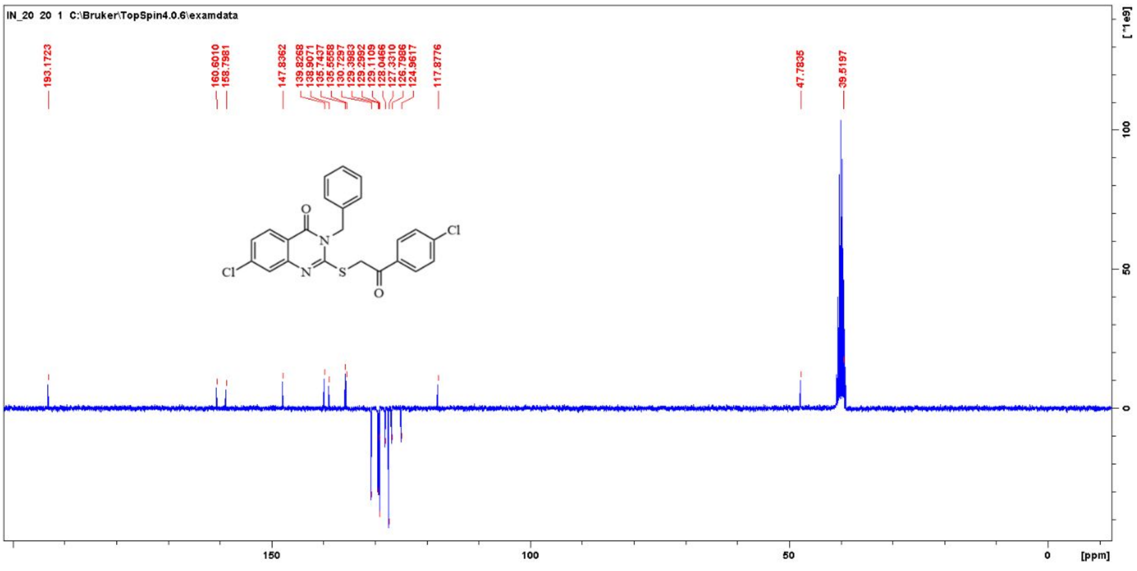

Fig. S64. ^{13}C-NMR for compound **7a**

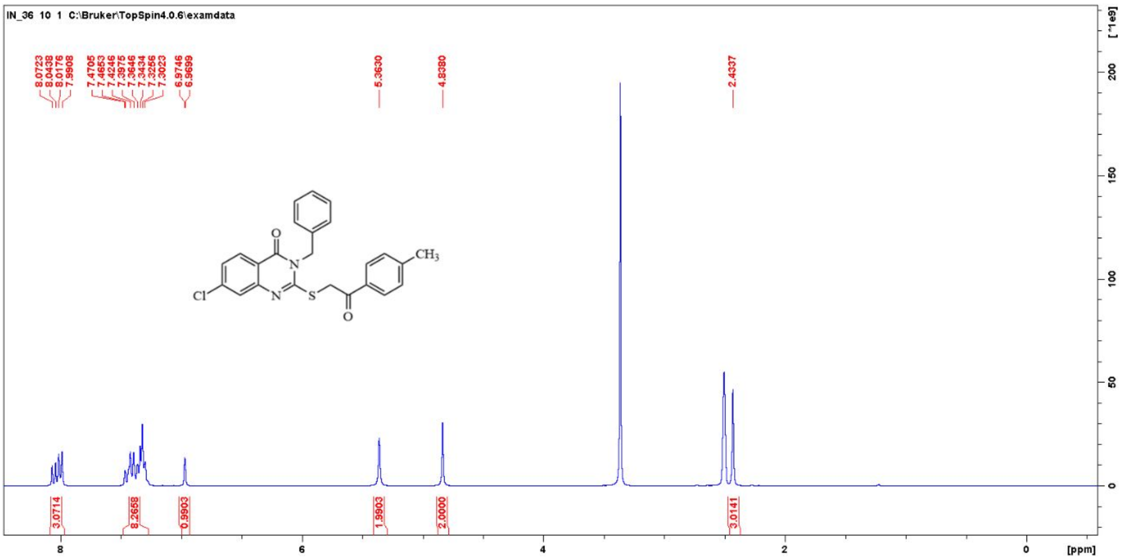

Fig. S65. ^{1}H-NMR for compound **7b**

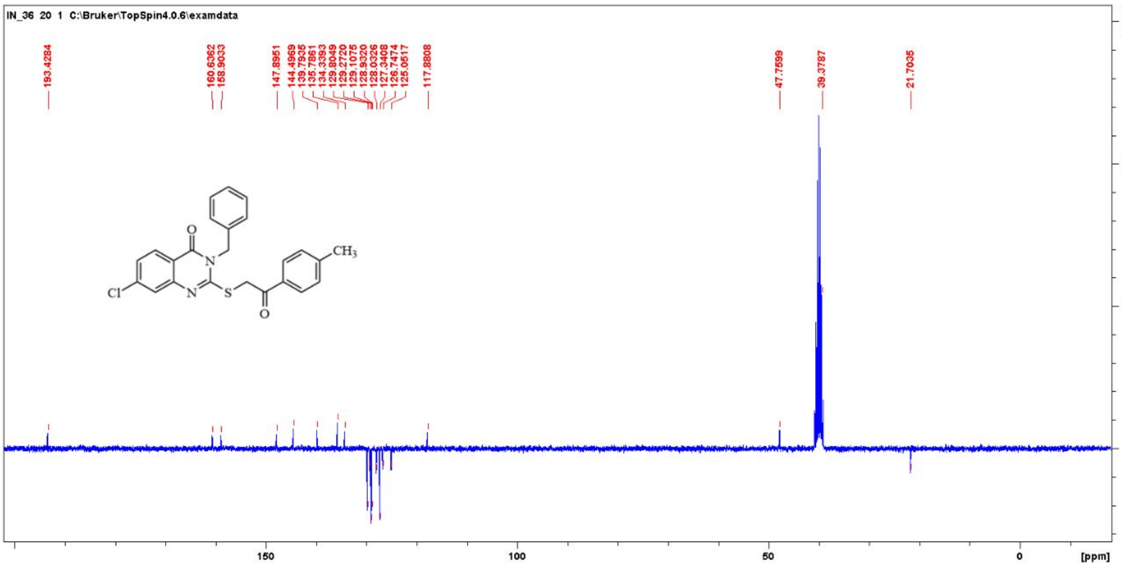

Fig. S66. ^{13}C-NMR for compound **7b**

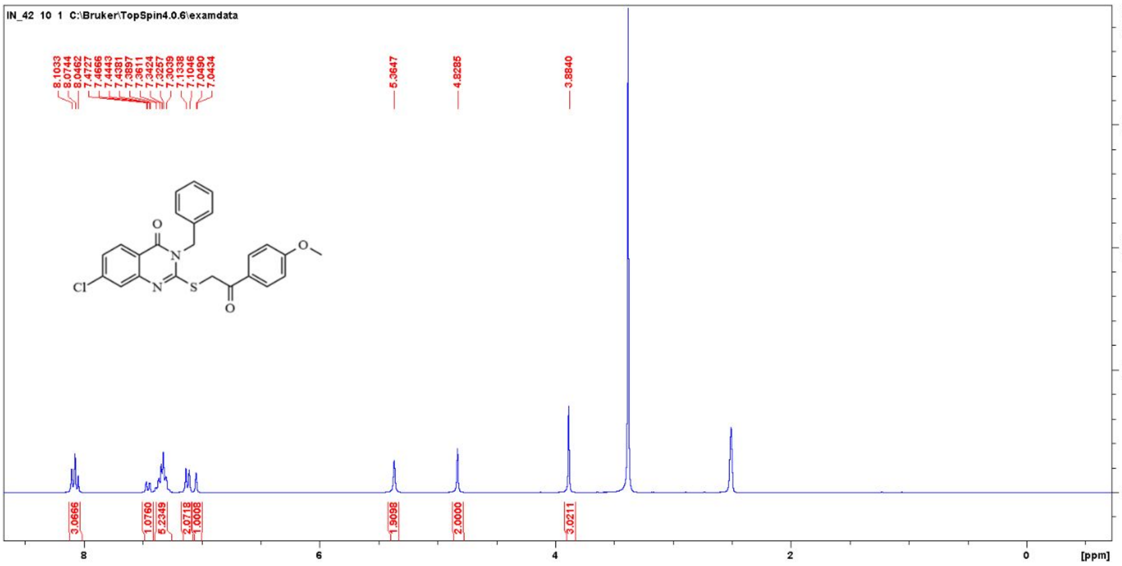

Fig. S67. ^{1}H-NMR for compound **7c**

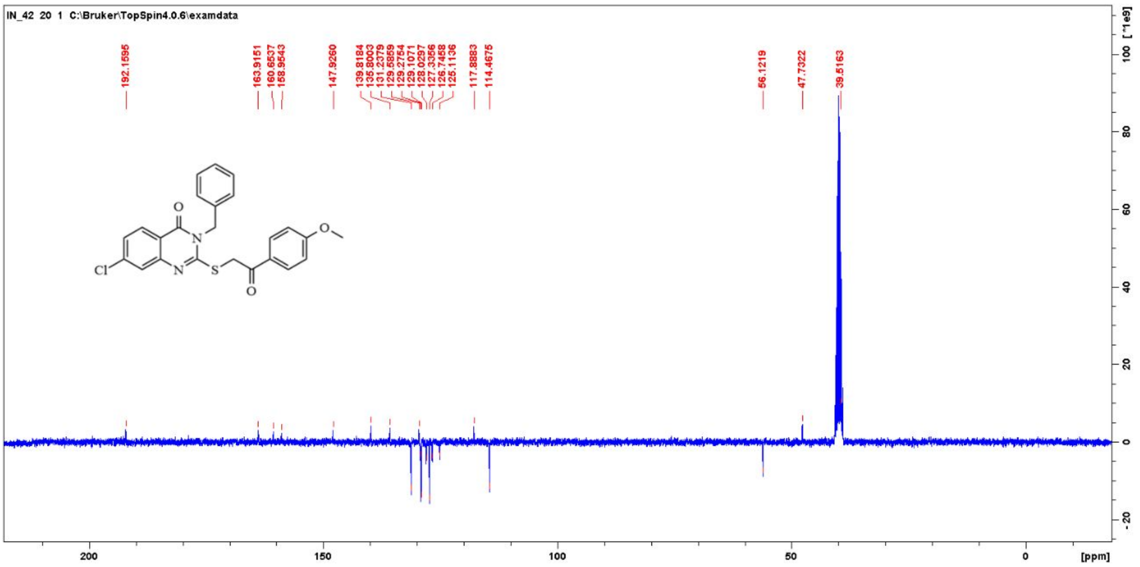

Fig. S68. ^{13}C-NMR for compound **7c**

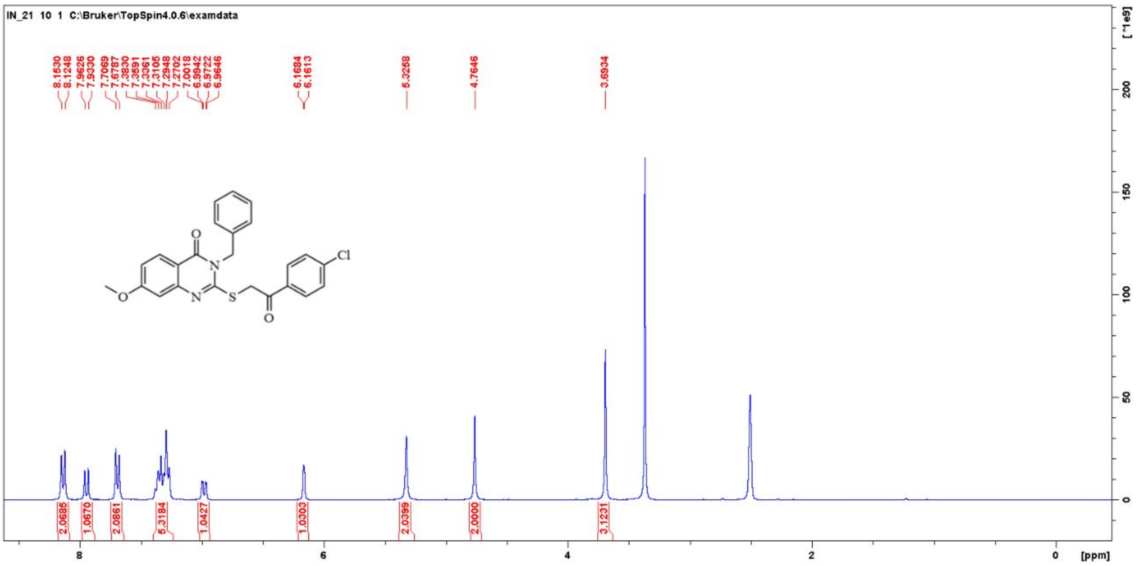

Fig. S69. ^{1}H-NMR for compound **8a**

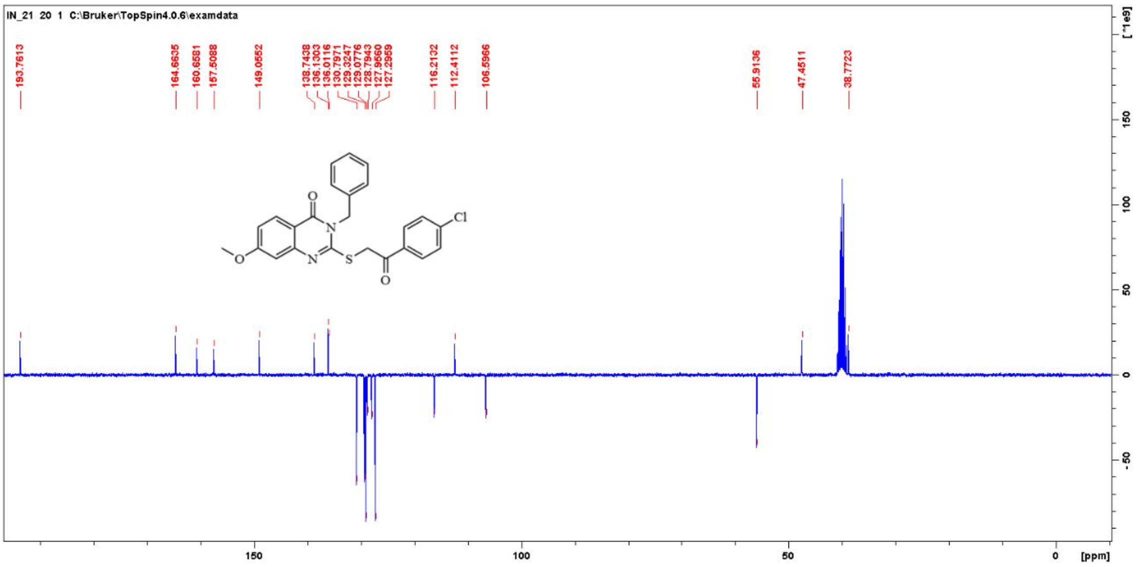

Fig. S70. ^{13}C-NMR for compound **8a**

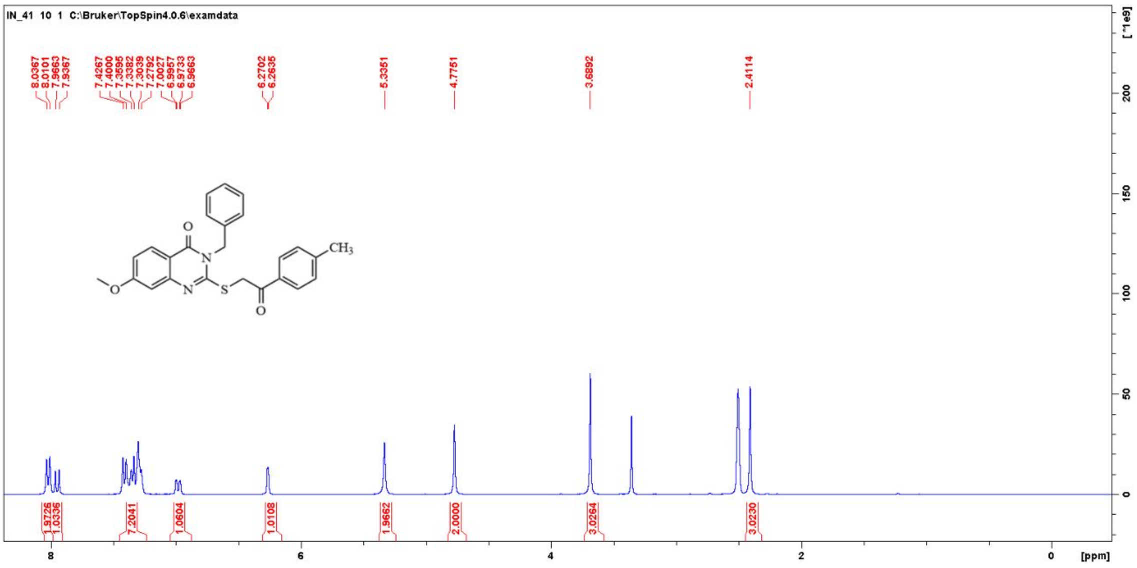

Fig. S71. ^{1}H-NMR for compound **8b**

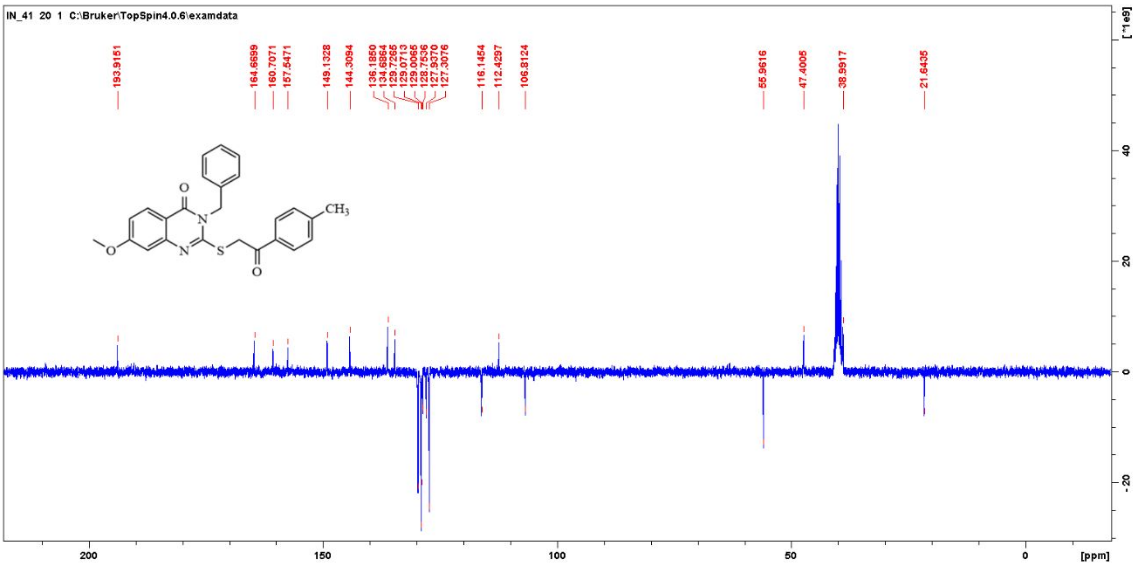

Fig. S72. ^{13}C-NMR for compound **8b**

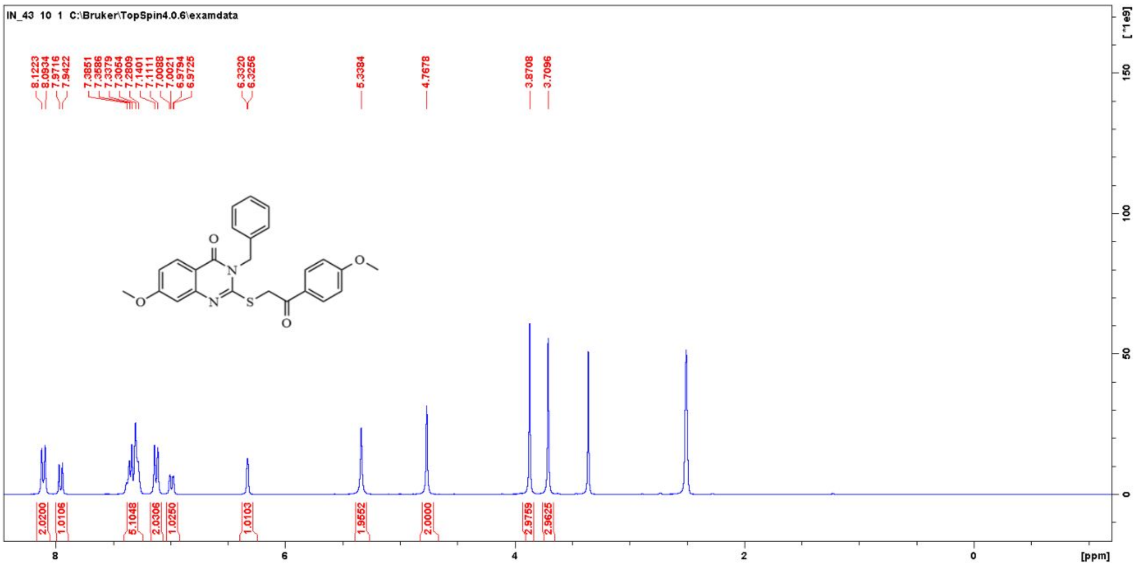

Fig. S73. ^{1}H-NMR for compound **8c**

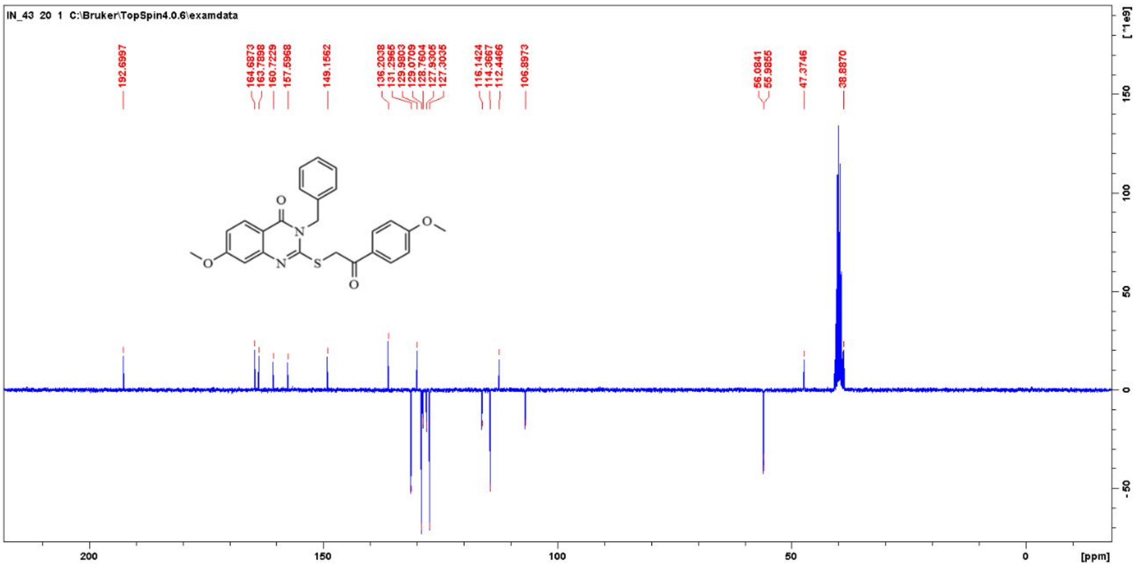

Fig. S74. ^{13}C-NMR for compound **8c**

2. High-resolution mass spectrometry (HRMS) of the compounds

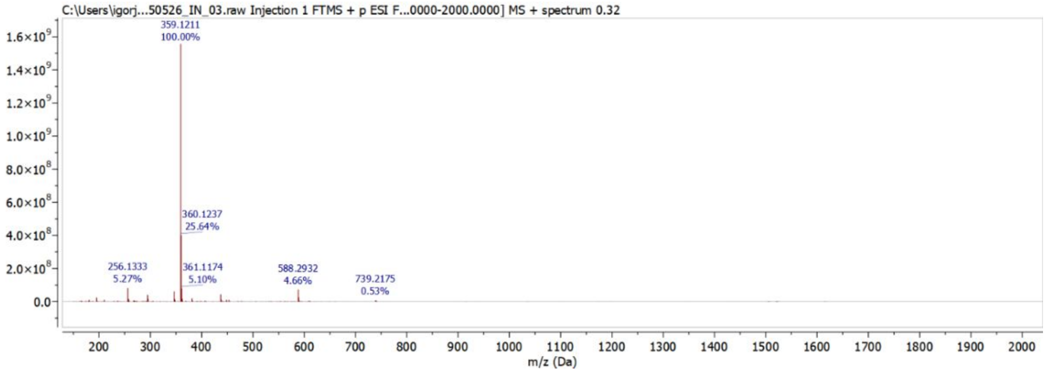

Fig. S75 HRMS for compound **1a**

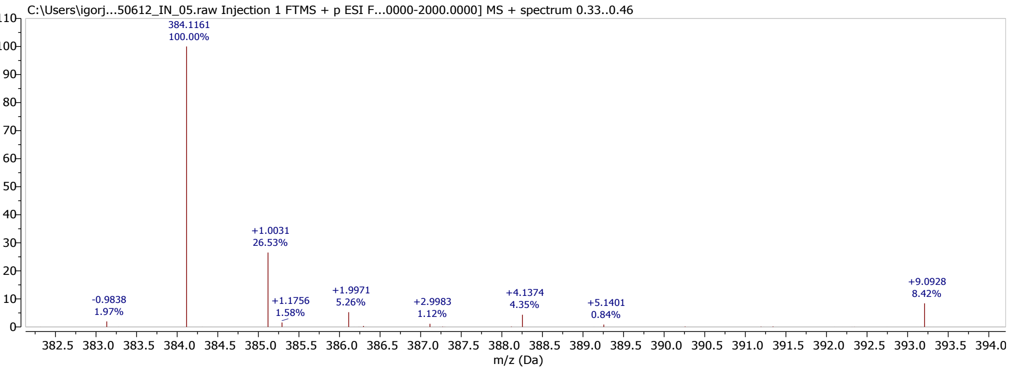

Fig. S76. HRMS for compound **1b**

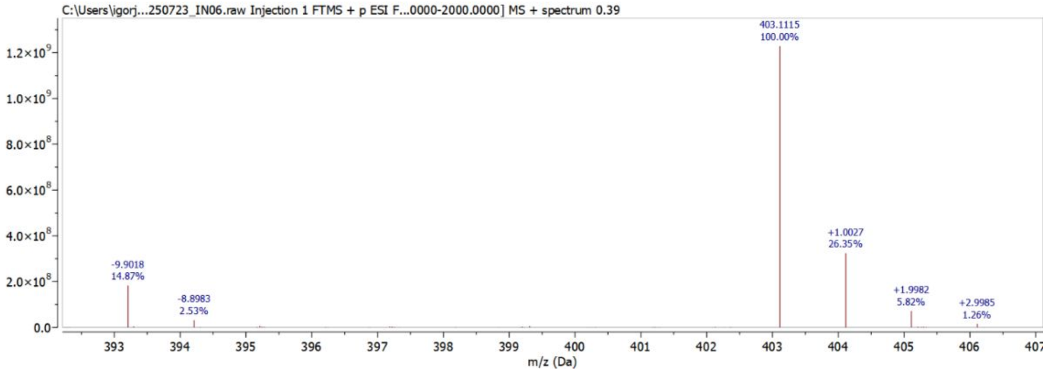

Fig. S77. HRMS for compound **1c**

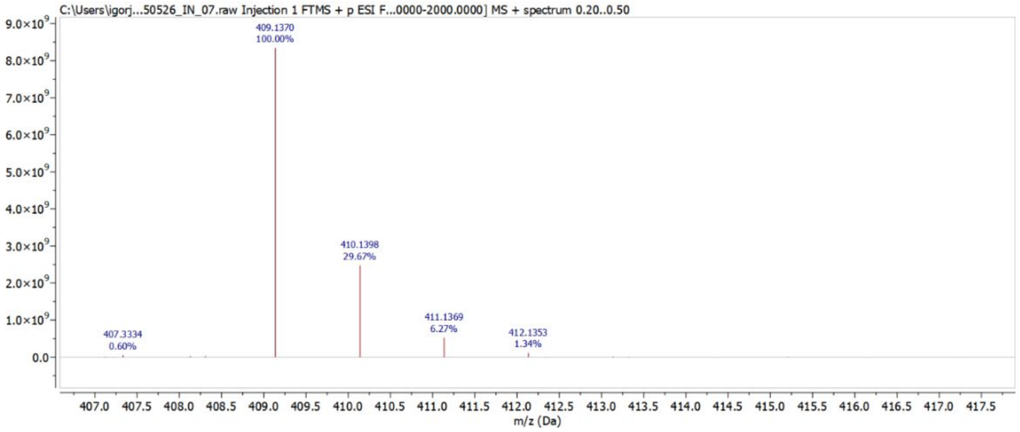

Fig. S78 HRMS for compound **1d**

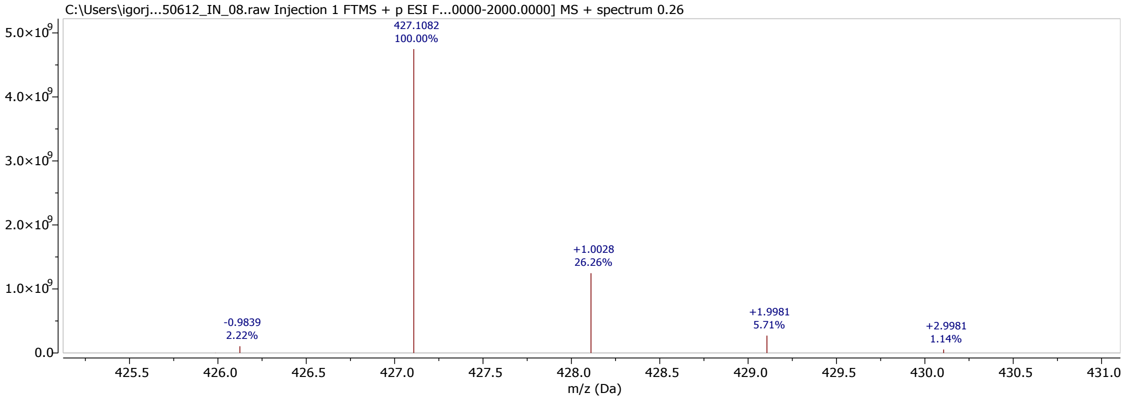

Fig. S79. HRMS for compound **1e**

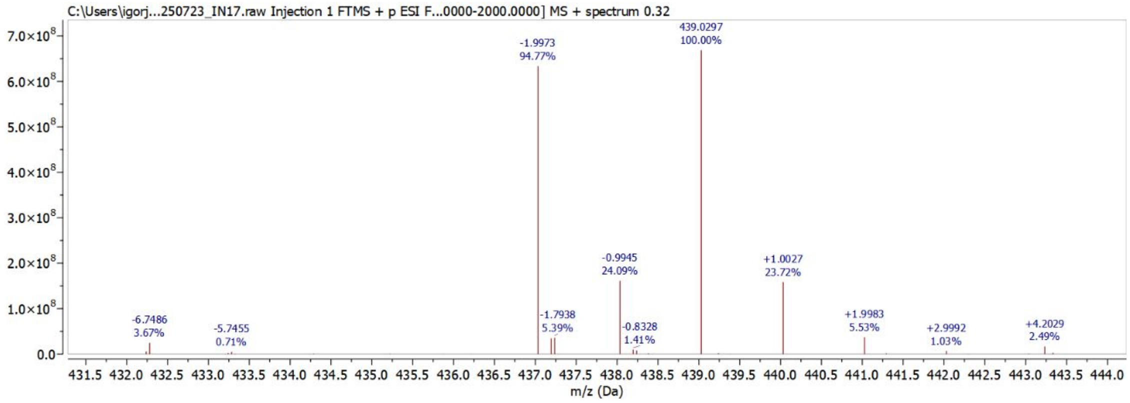

Fig. S80. HRMS for compound **1f**

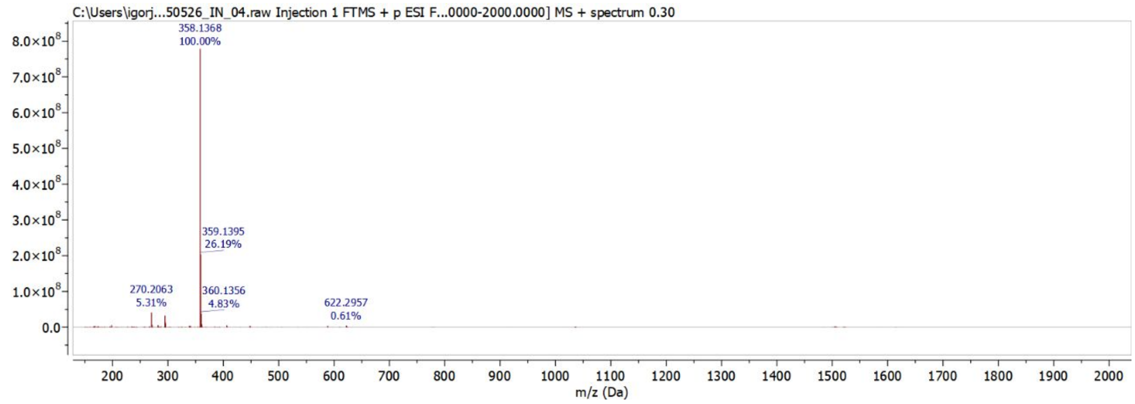

Fig. S81. HRMS for compound **2a**

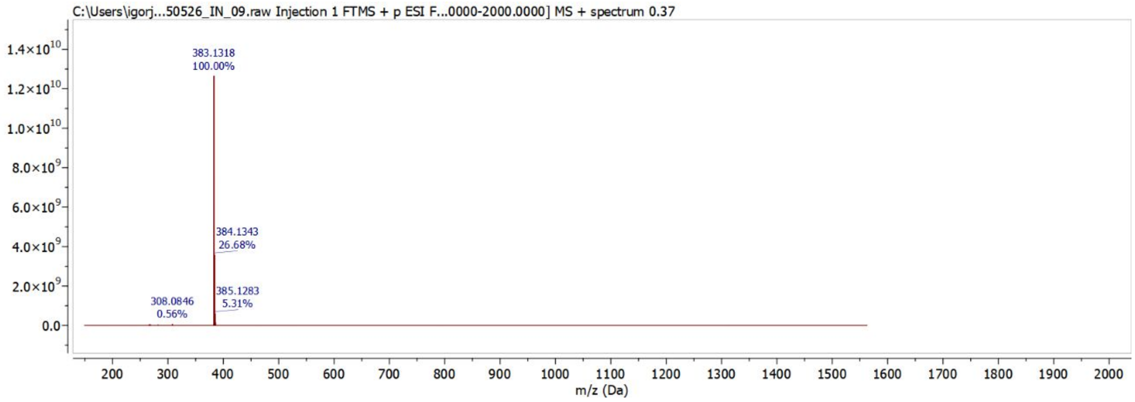

Fig. S82. HRMS for compound **2b**

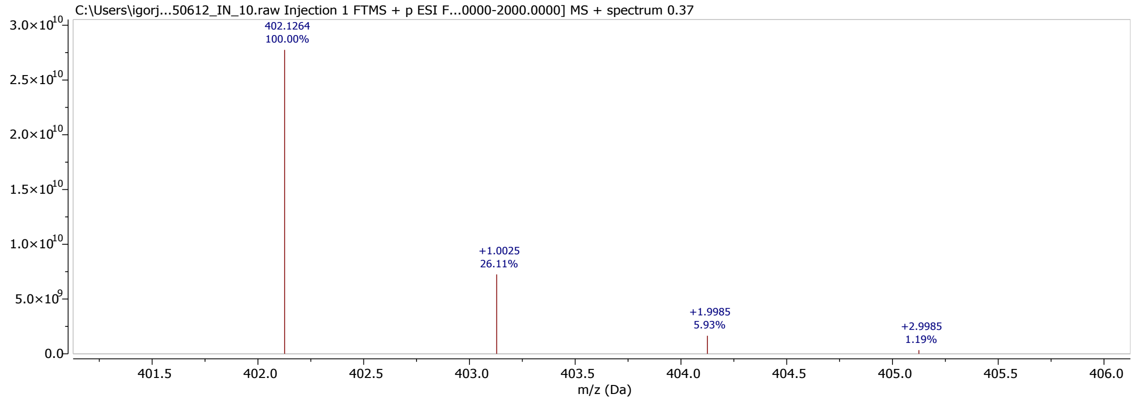

Fig. S83. HRMS for compound **2c**

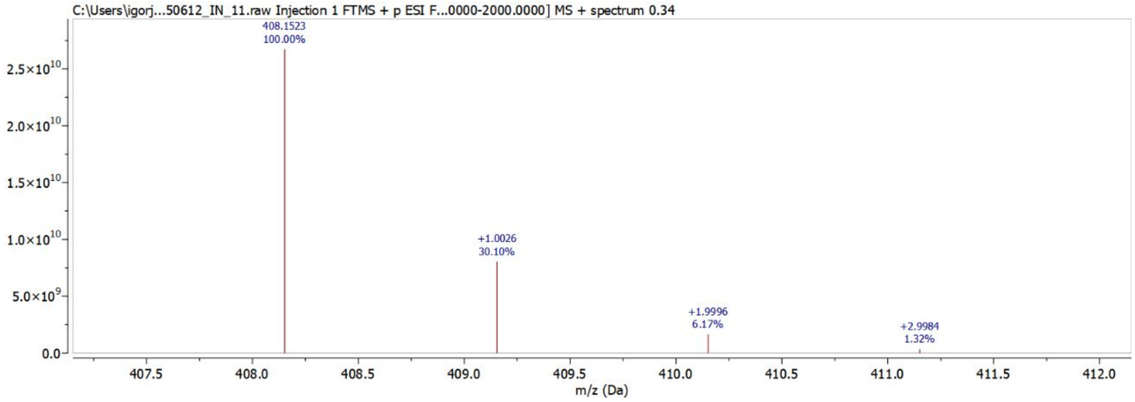

Fig. S84. HRMS for compound **2d**

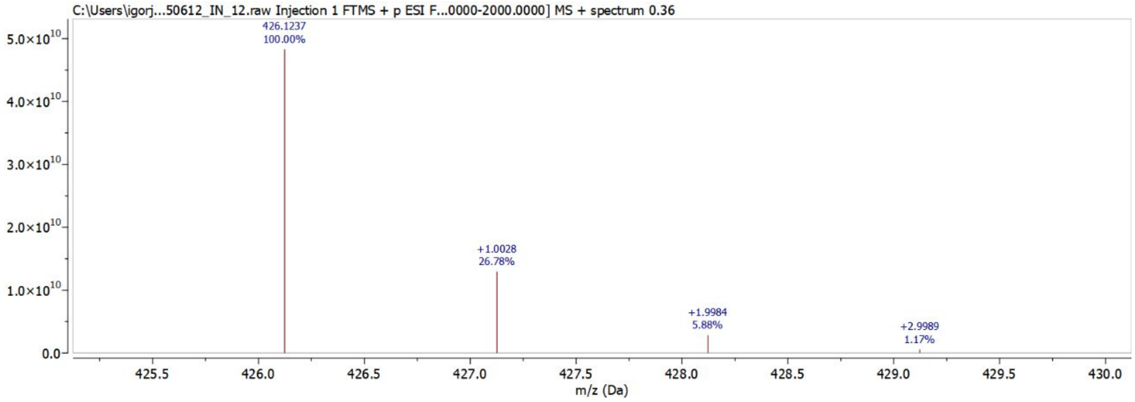

Fig. S85. HRMS for compound **2e**

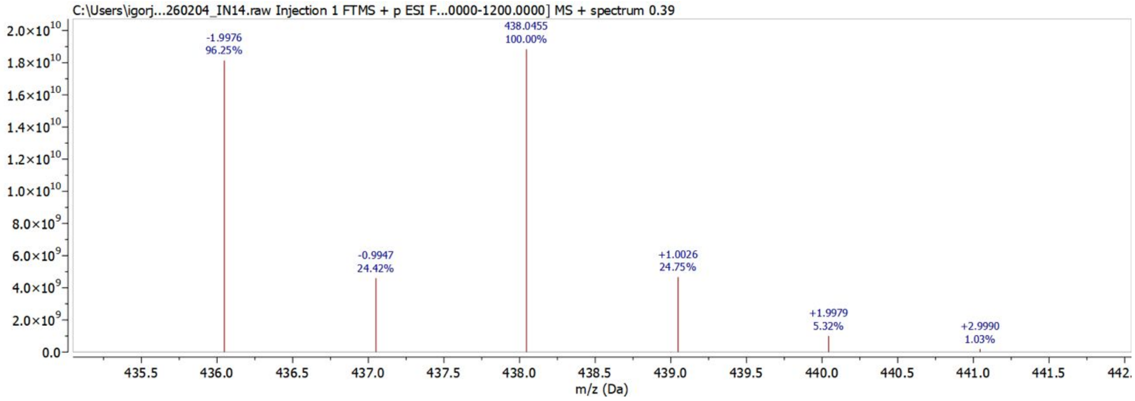

Fig. S86. HRMS for compound **2f**

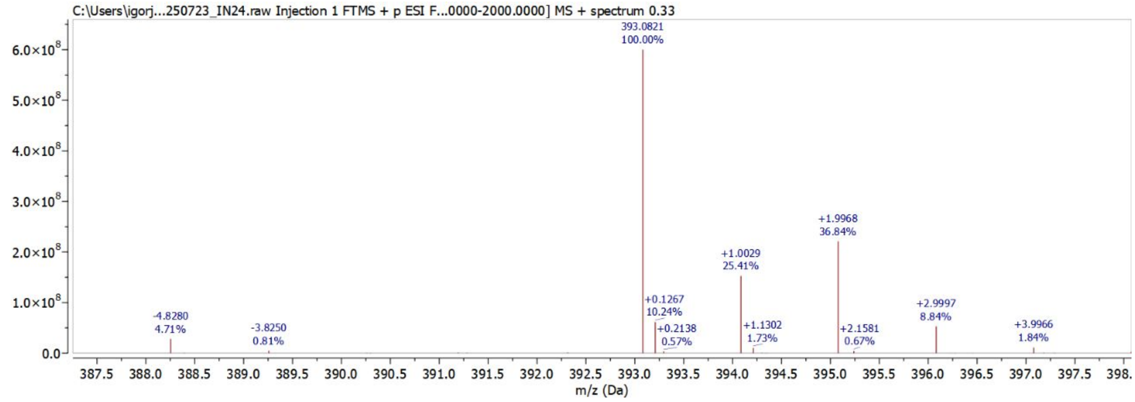

Fig. S87. HRMS for compound **3a**

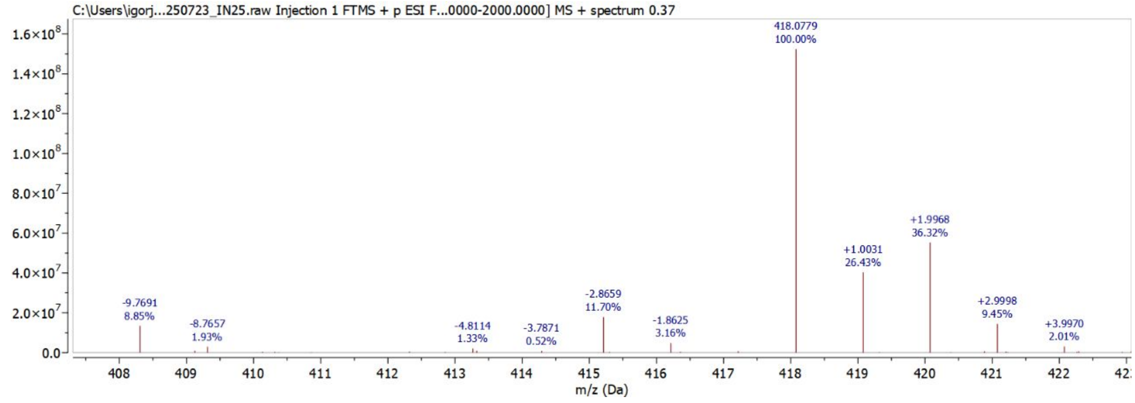

Fig. S88. HRMS for compound **3b**

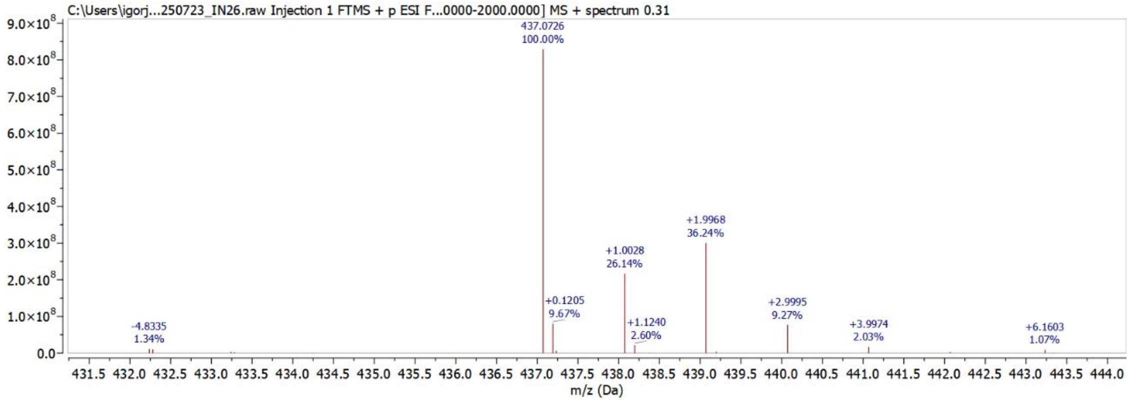

Fig. S89. HRMS for compound **3c**

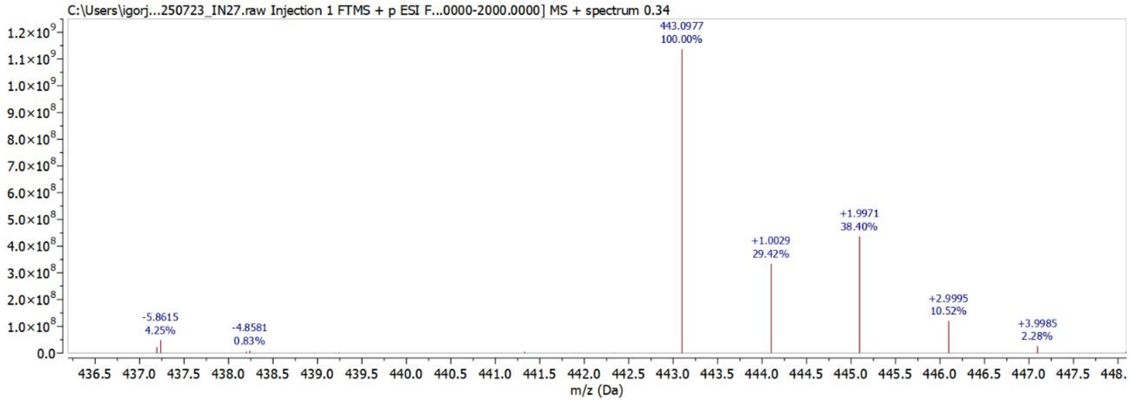

Fig. S90. HRMS for compound **3d**

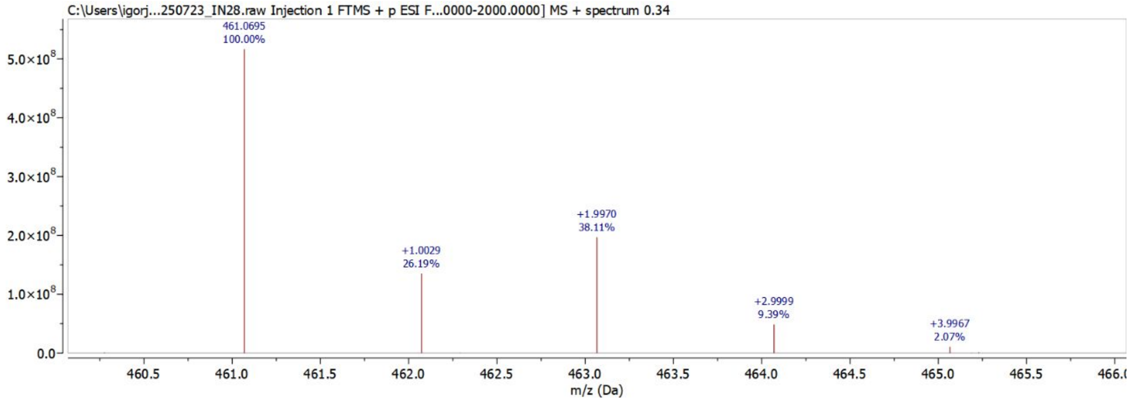

Fig. S91. HRMS for compound **3e**

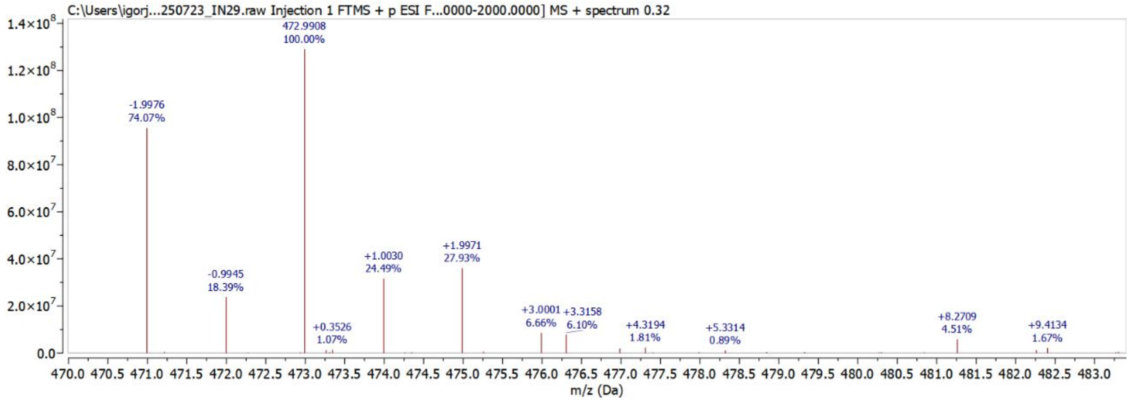

Fig. S92. HRMS for compound **3f**

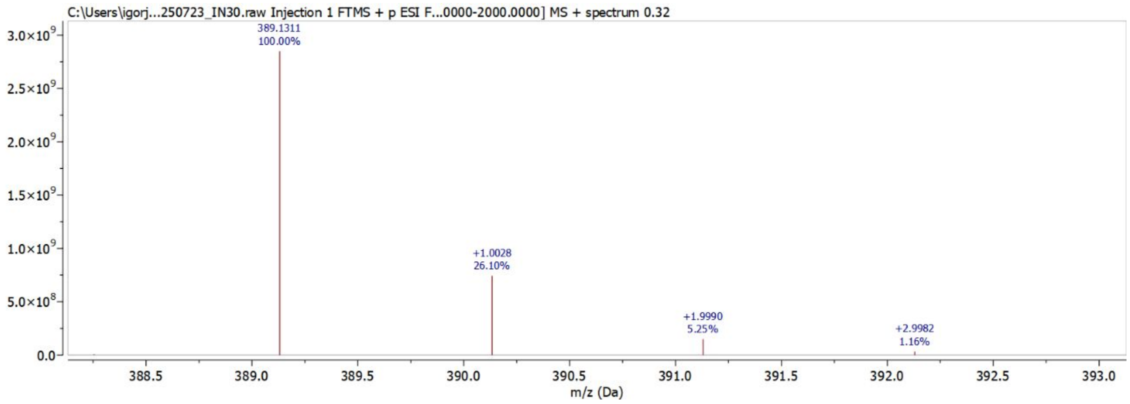

Fig. S93. HRMS for compound **4a**

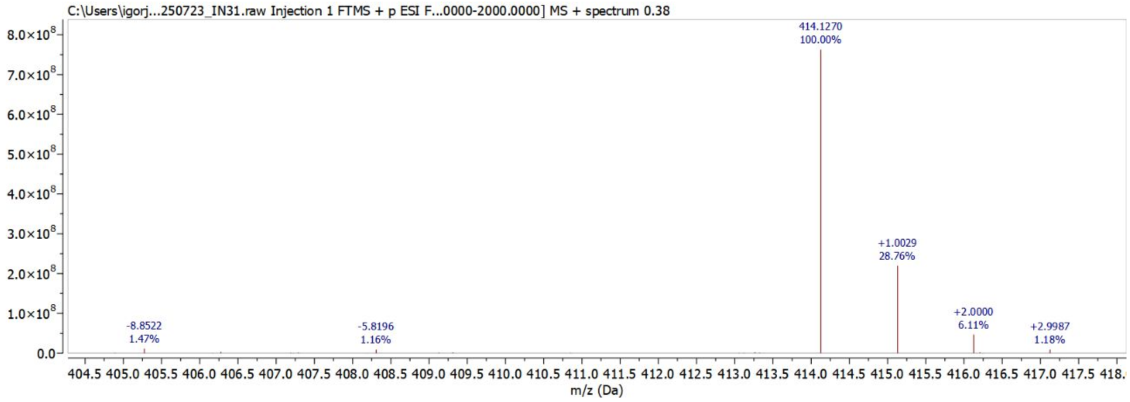

Fig. S94. HRMS for compound **3b**

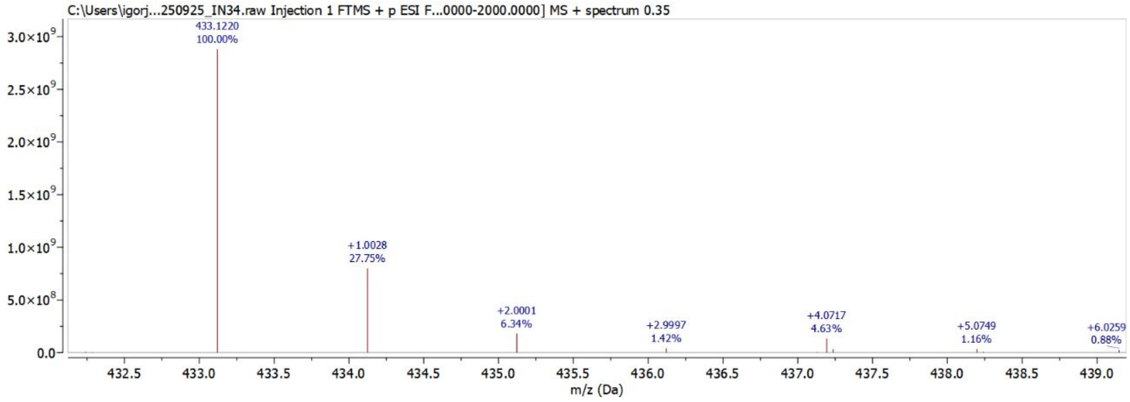

Fig. S95. HRMS for compound **4c**

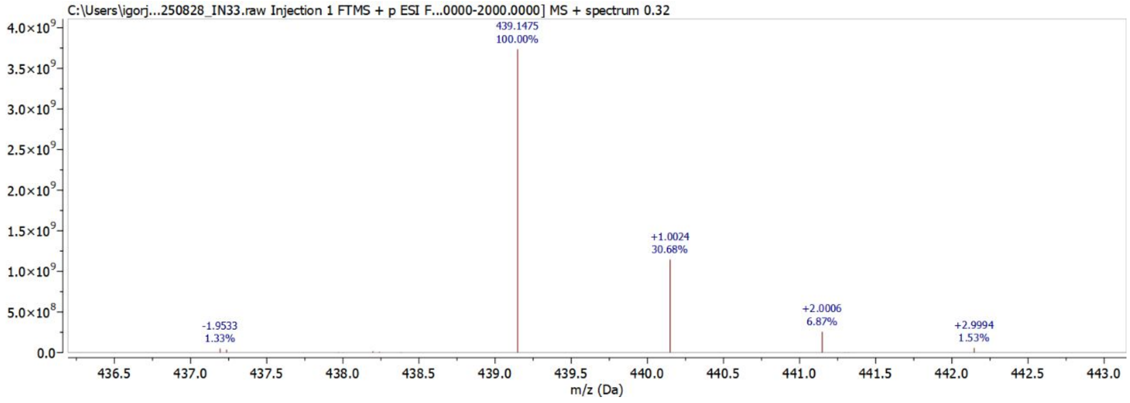

Fig. S96. HRMS for compound **4d**

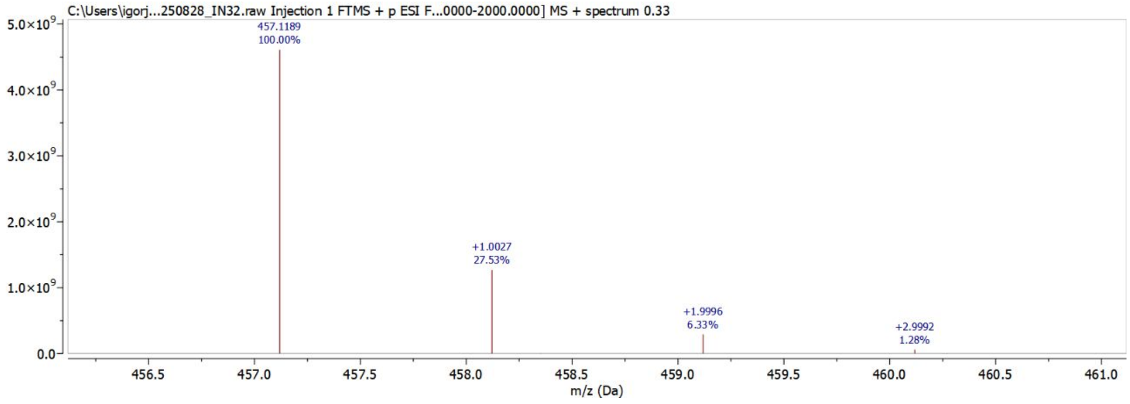

Fig. S97. HRMS for compound **4e**

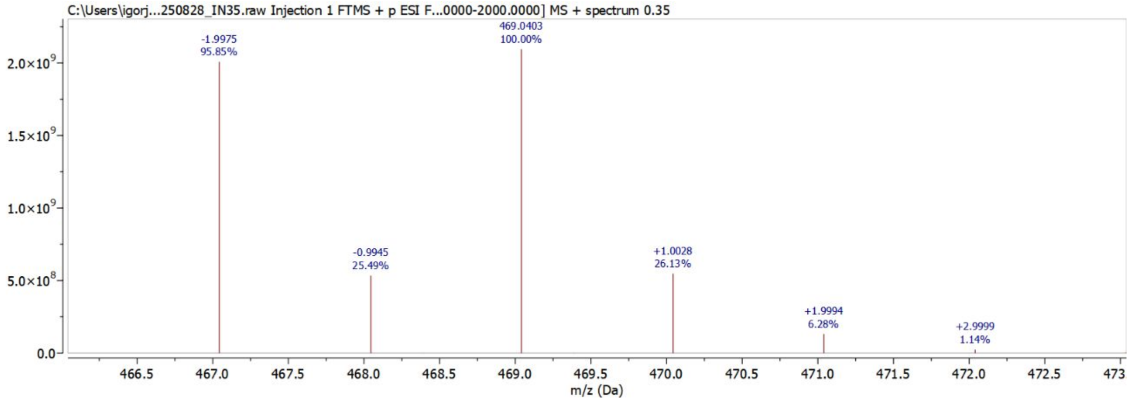

Fig. S98. HRMS for compound **4f**

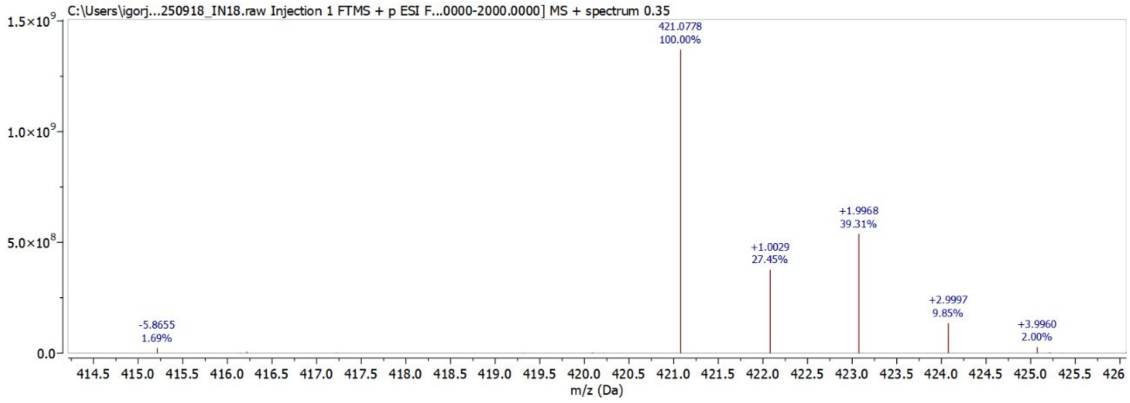

Fig. S99. HRMS for compound **5a**

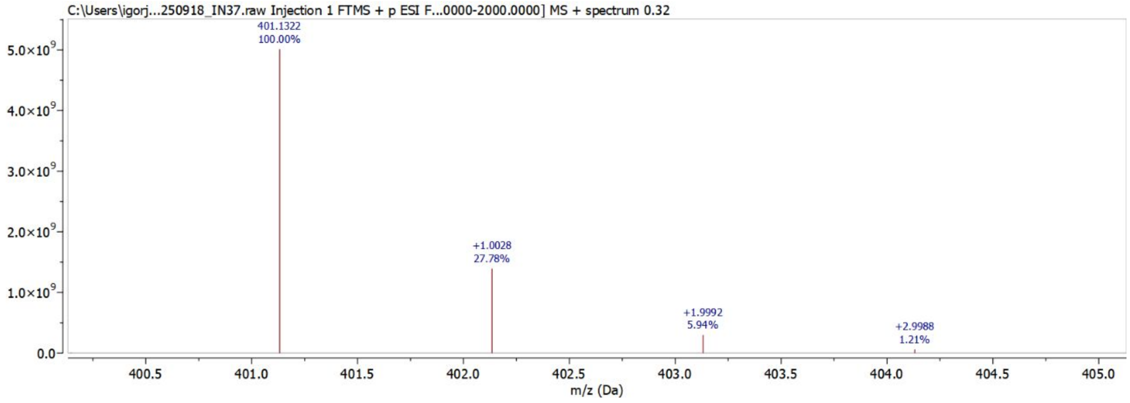

Fig. S100. HRMS for compound **5b**

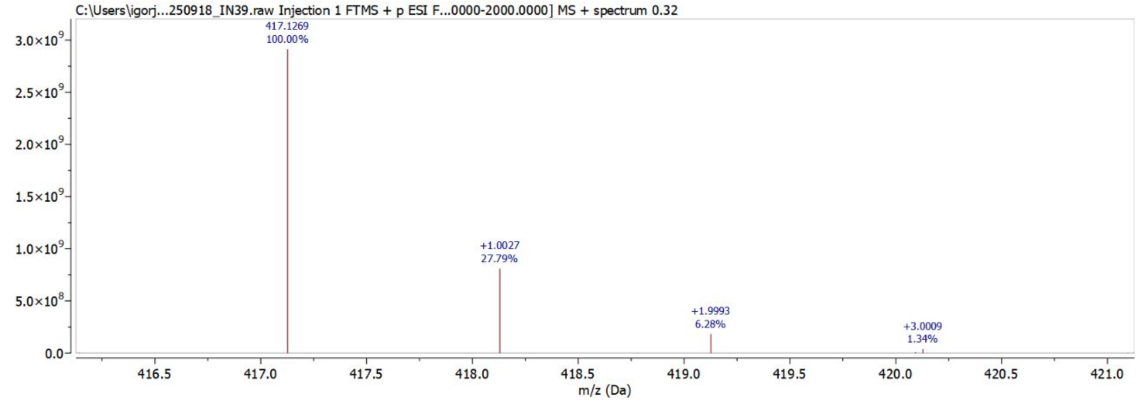

Fig. S101. HRMS for compound **5c**

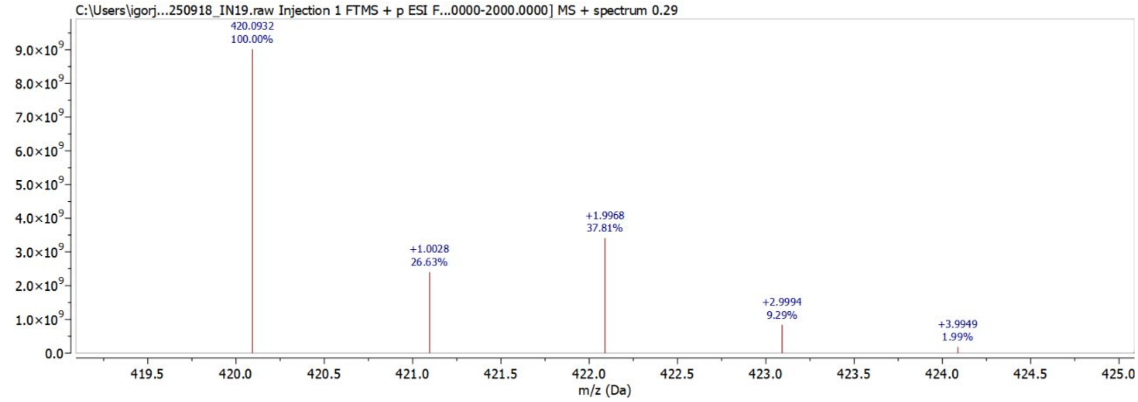

Fig. S102. HRMS for compound **6a**

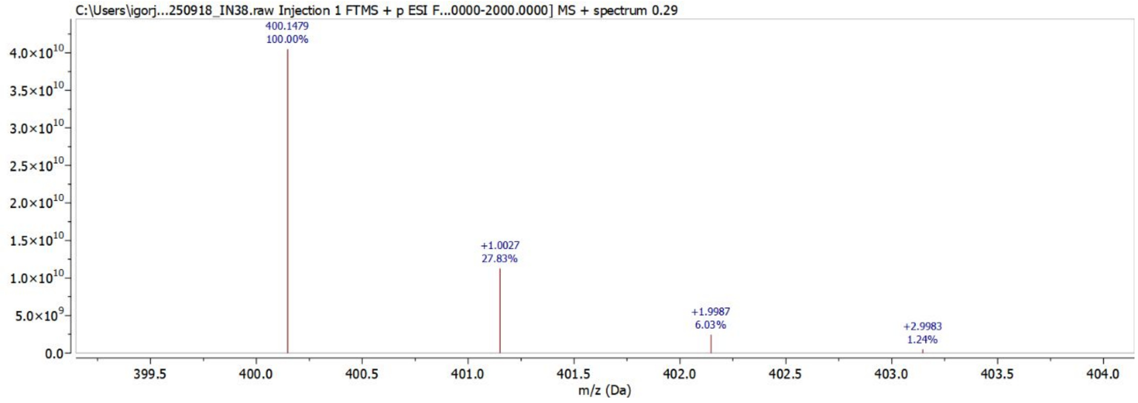

Fig. S103. HRMS for compound **6b**

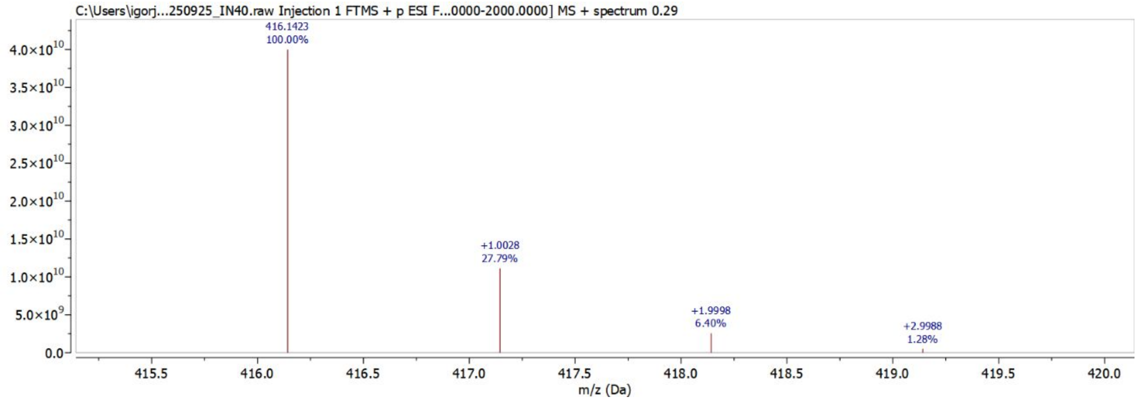

Fig. S104. HRMS for compound **6c**

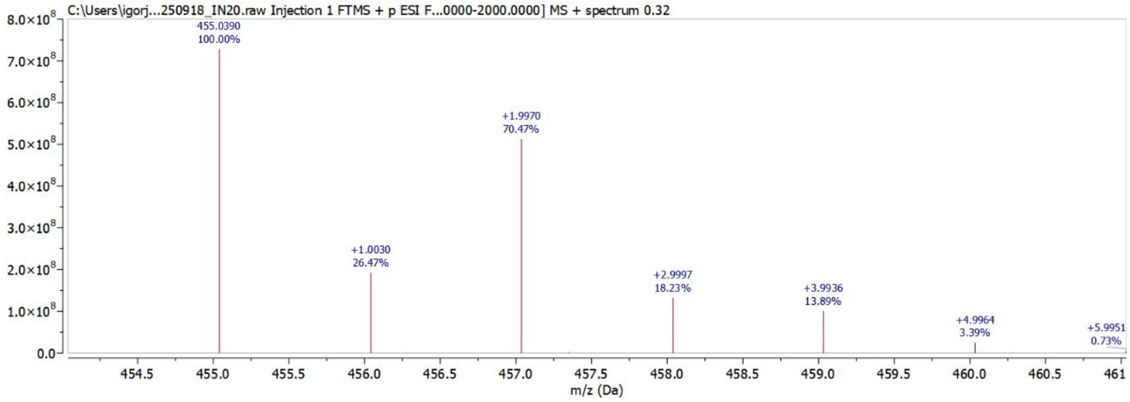

Fig. S105. HRMS for compound **7a**

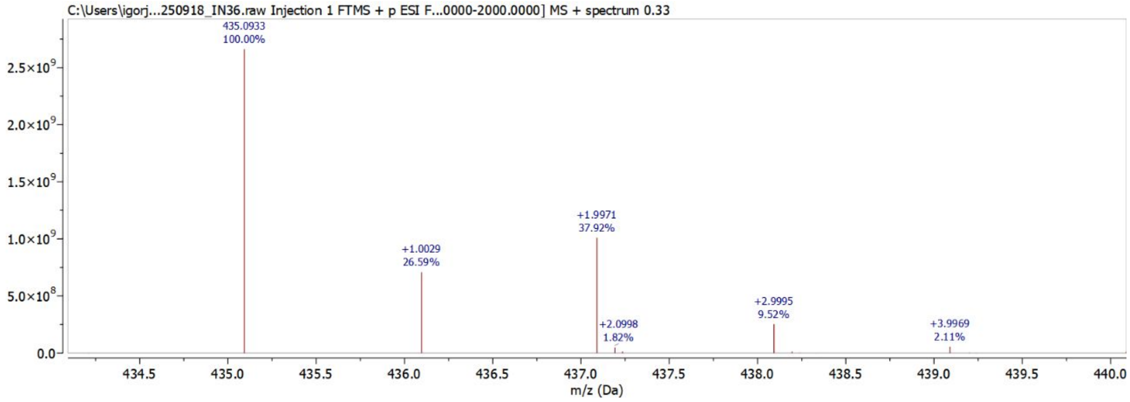

Fig. S106. HRMS for compound **7b**

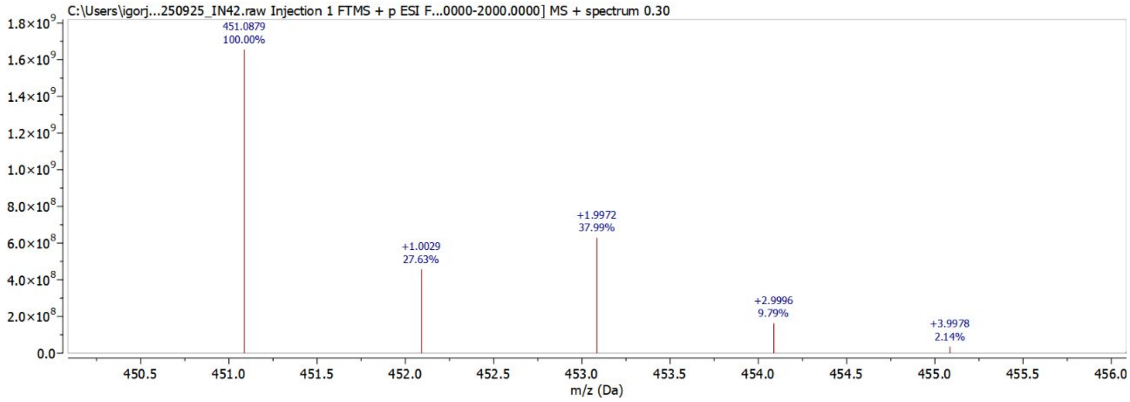

Fig. S107. HRMS for compound **7c**

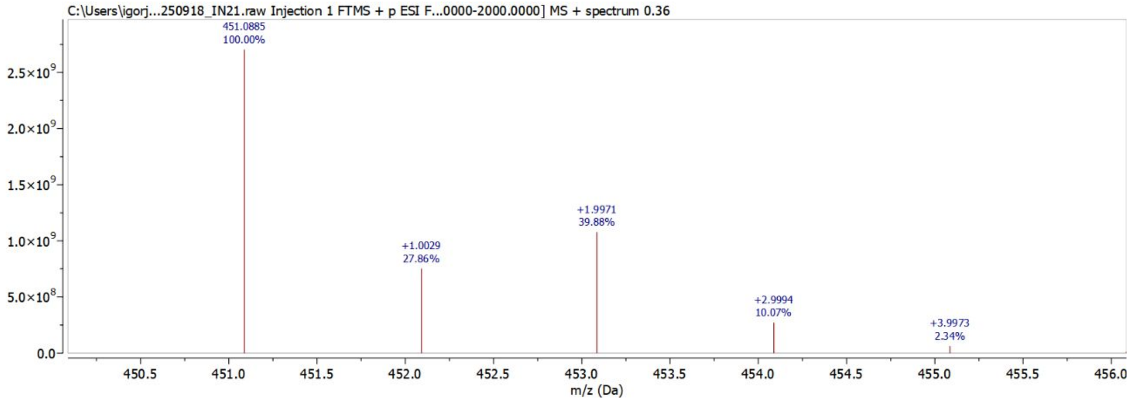

Fig. S108. HRMS for compound **8a**

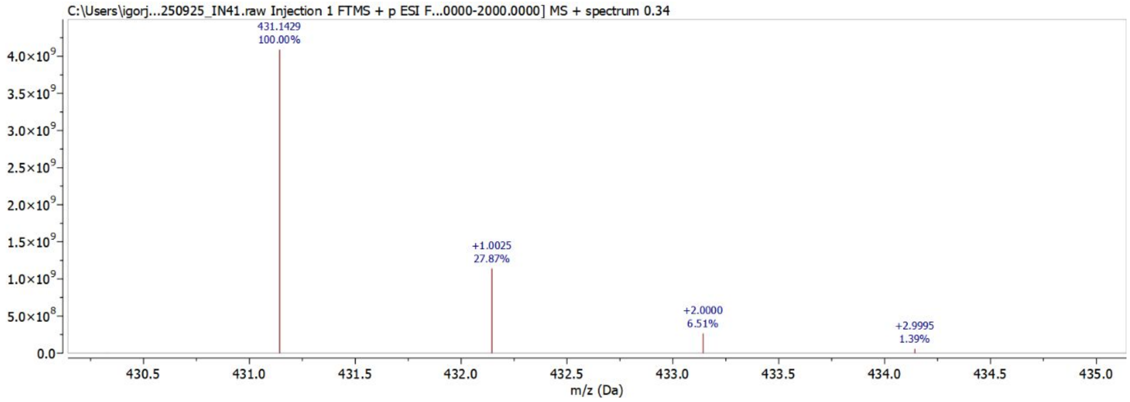

Fig. S109. HRMS for compound **8b**

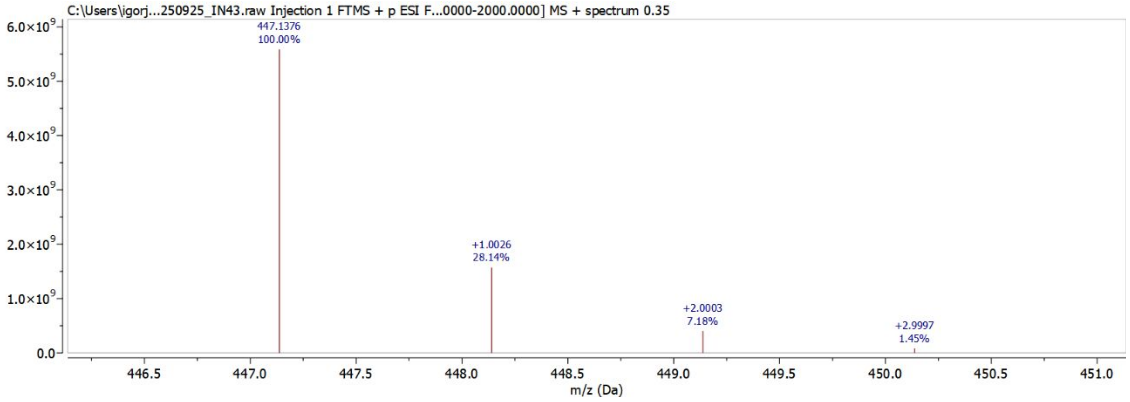

Fig. S110. HRMS for compound **8c**

3. Plots of Half-maximal inhibitory (IC_{50}) of the Antimalarial Assay using *P. falciparum* 3D7HT-GFP Cell Line.

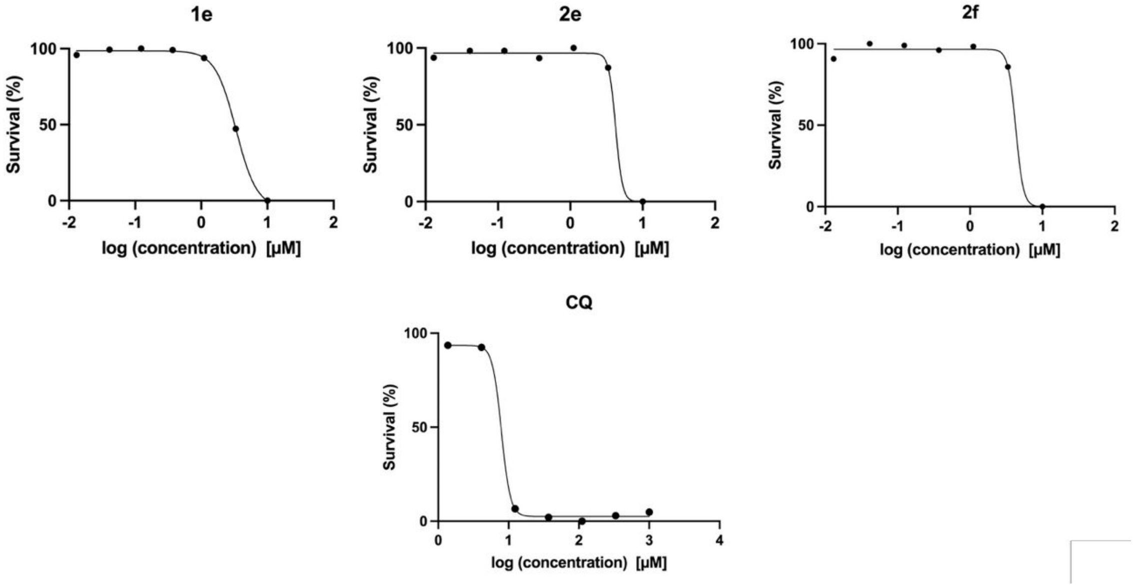

Fig. S111. IC_{50} plots of the compounds **1e**, **2e**, **2f**, and chloroquine (**CQ**).

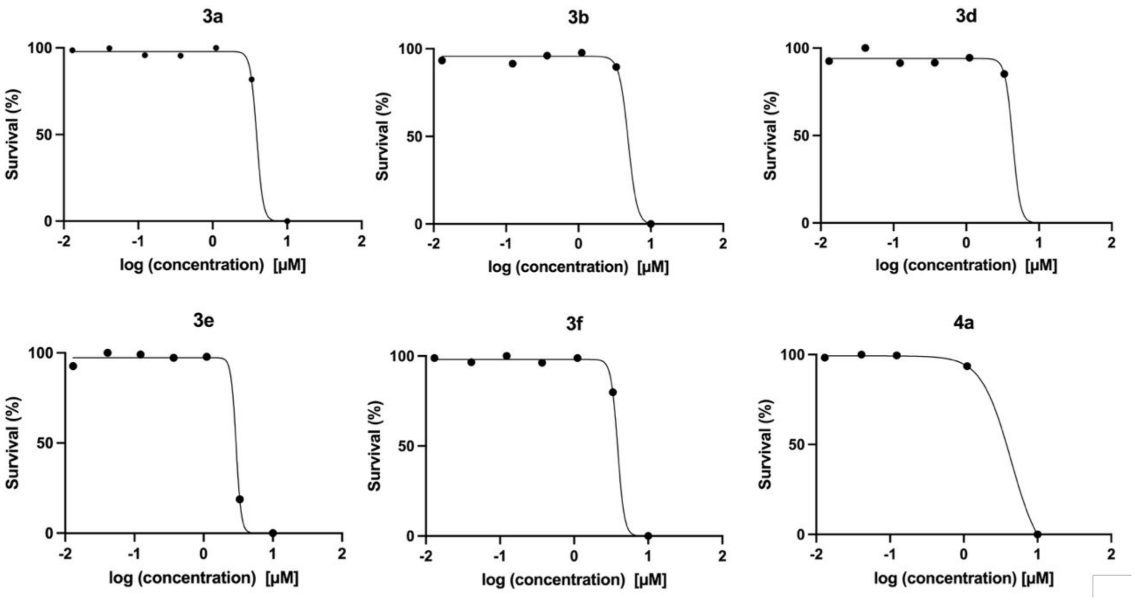

Fig. S112. IC_{50} plots of the compounds **3a, 3b, 3d, 3e, 3f,** and **4a**

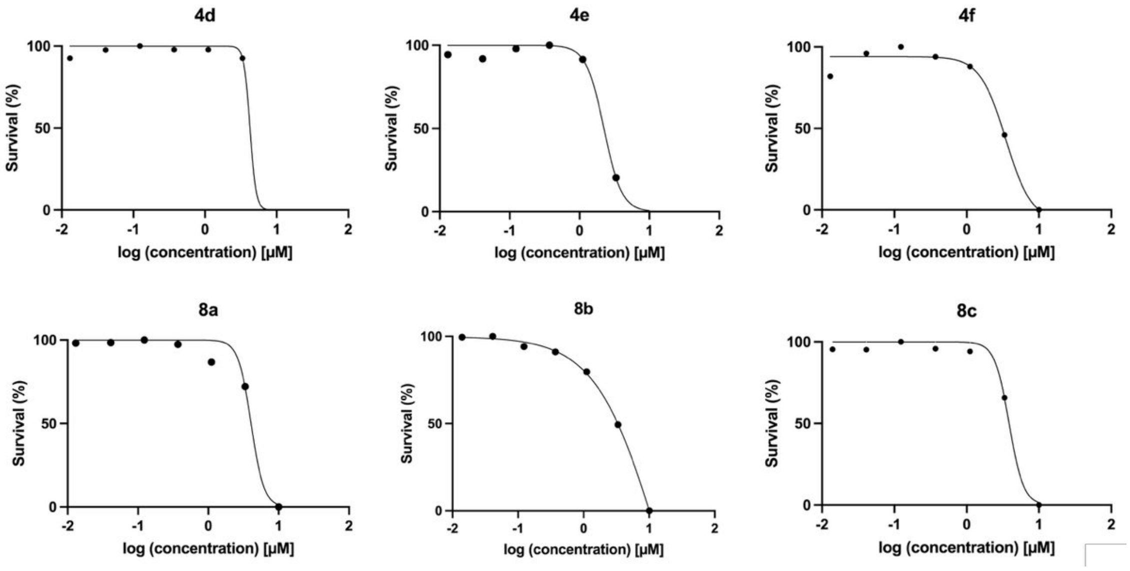

Fig. S113. IC_{50} plots of the compounds **4d**, **4e**, **4f**, **8a**, **8b**, and **8c**.

4. Plots of cytotoxic concentration (CC_{50})

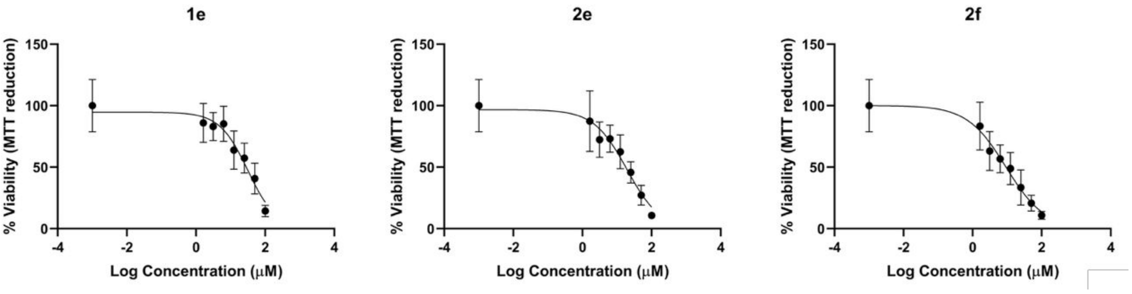

Fig. S114. CC_{50} plots of the compounds **1e**, **2e**, and **2f**

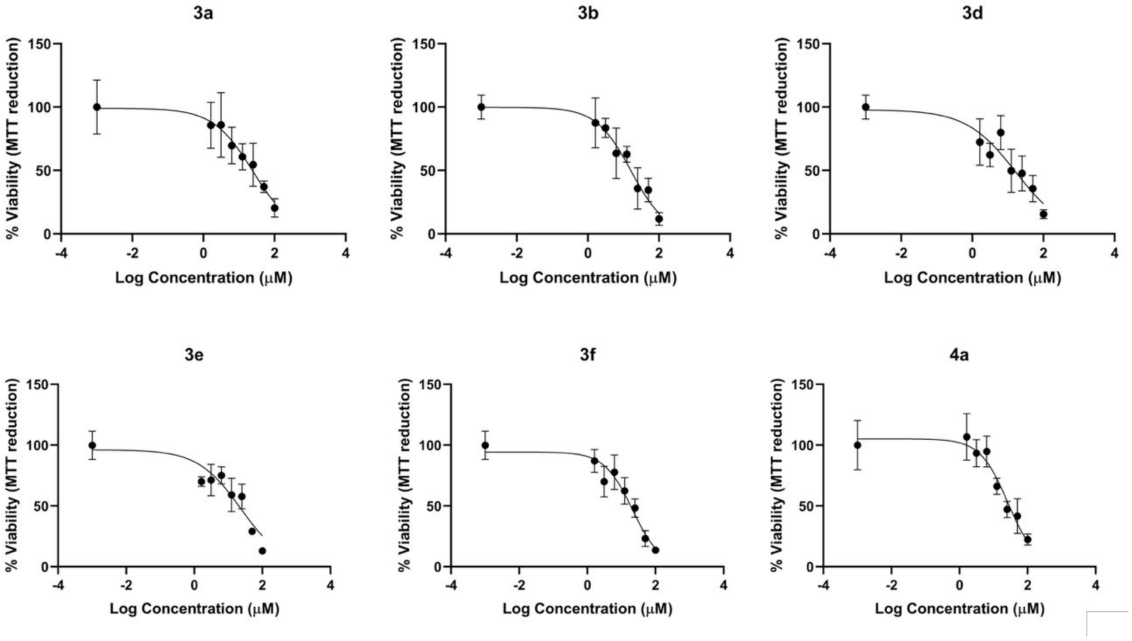

Fig. S115. CC_{50} plots of the compounds **3a, 3b, 3d, 3e, 3f,** and **4a**

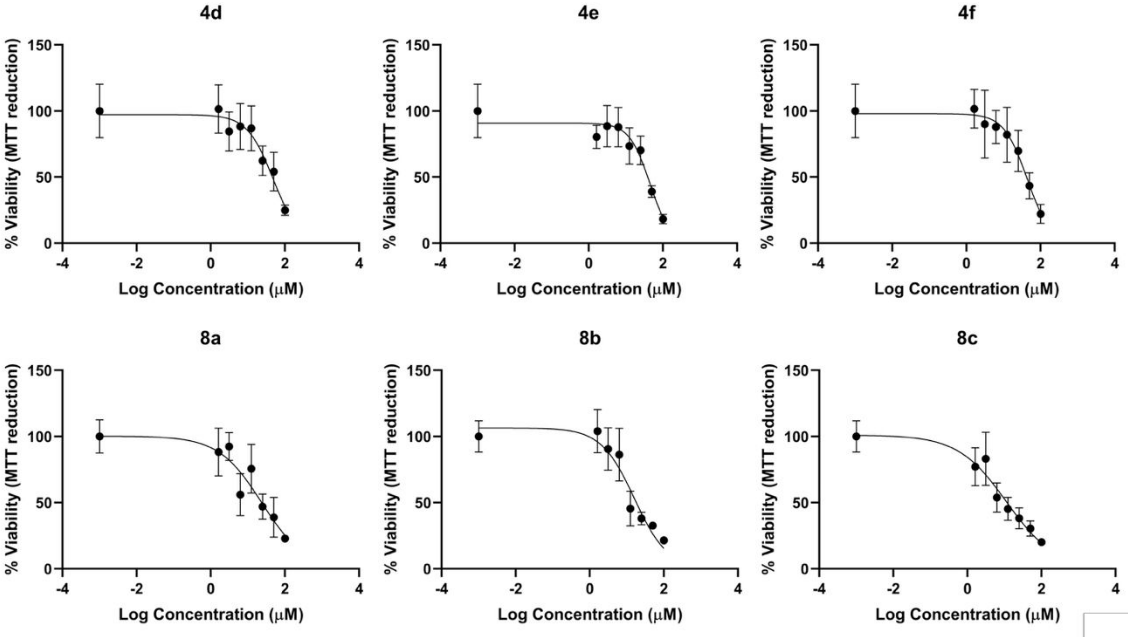

Fig. S116. CC_{50} plots of the compounds **4d**, **4e**, **4f**, **8a**, **8b**, and **8c**.

5. Plots of Molecular Dynamics Simulations

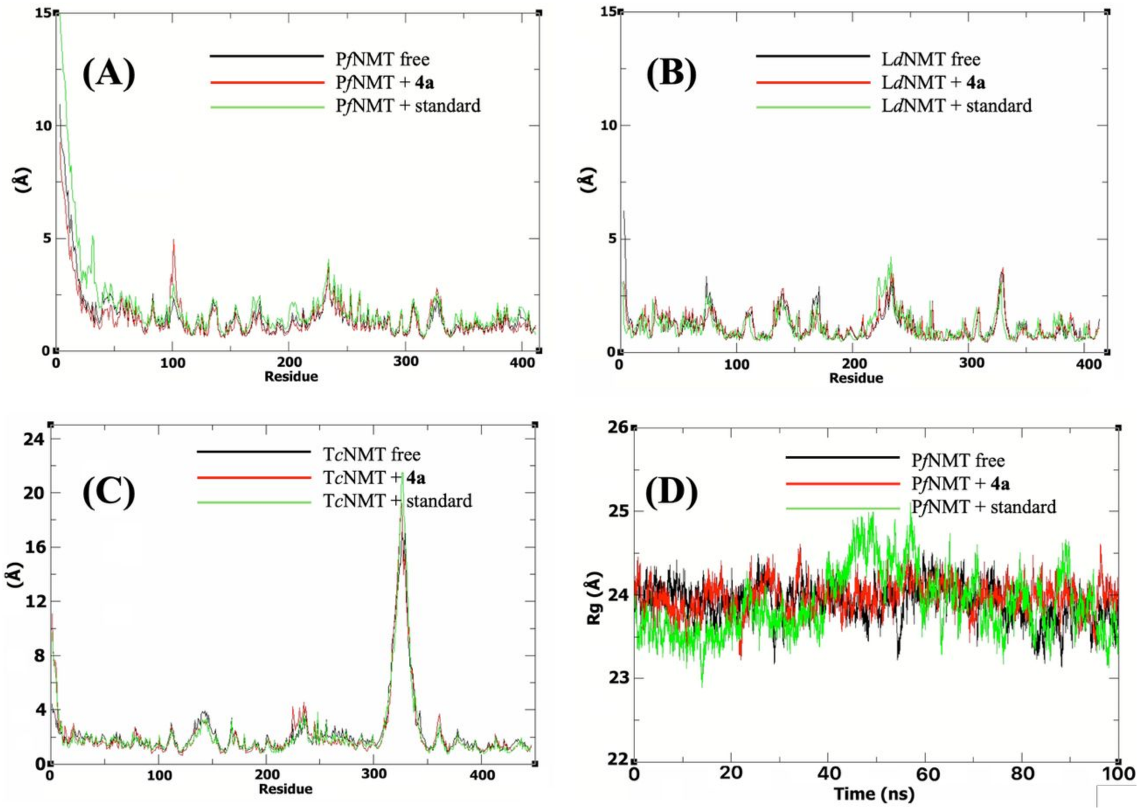

Fig. S117. Molecular dynamics simulation results of RMSF for the: **(A)** P*f*NMT free (black line), in complex with **4a** (red line), and standard compound (green line); **(B)** L*d*NMT free (black line), and in complex with **4a** (red line), and the standard compound (green line); **(C)** T*c*NMT free (black line) and in complex with **4a** (red line) and standard compound (green line); and **(D)** R_g results for the P*f*NMT free (black line) and in complex with **4a** (red line) and the standard compound (green line).

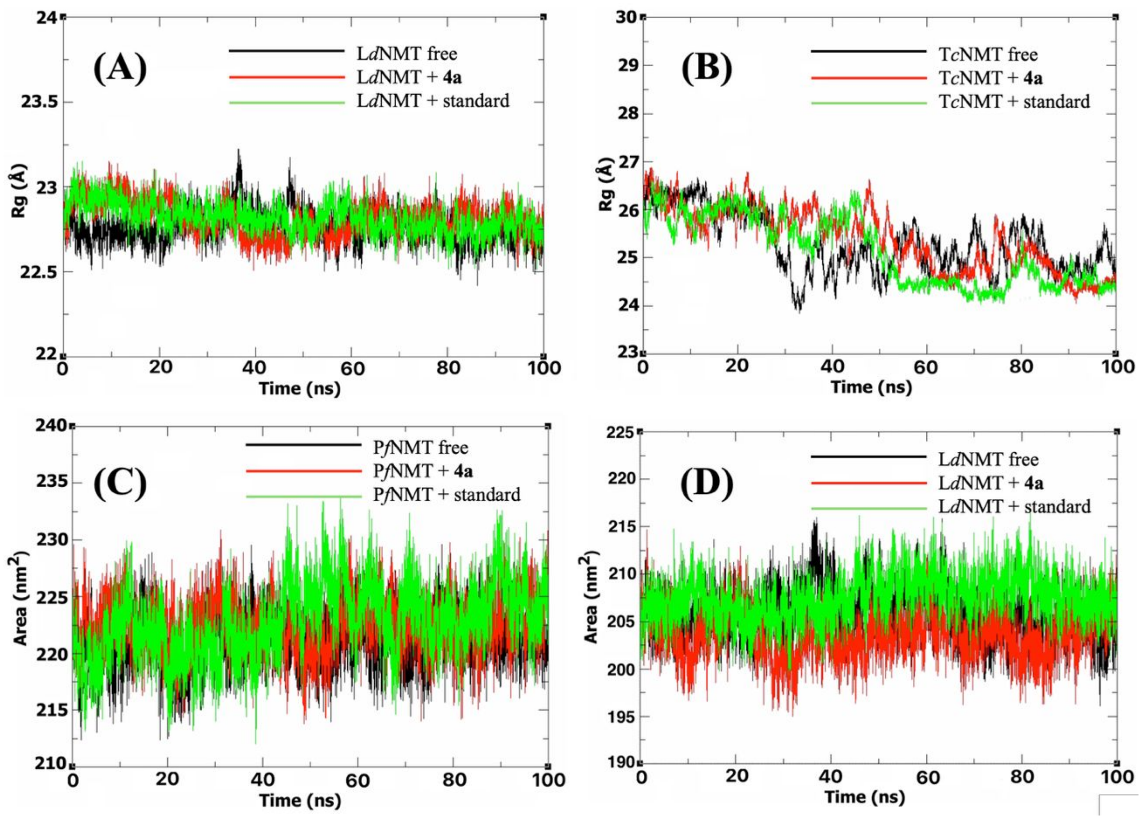

Fig. S118. Molecular dynamics simulations results of R_g for the: **(A)** L*d*NMt free (black line) and complexed with **4a** (red line) and standard compound (green line); **(B)** T*c*NMT free (black line) and complexed with **4a** (red line) and standard compound (green line); **(C)** the results of SASA of the protein for the P*f*NMT free (black line) and complexed with **4a** (red line) and standard compound (green line); and **(D)** SASA of the protein of the L*d*NMT free (black line) and complexed with **4a** (red line) and standard compound (green line).

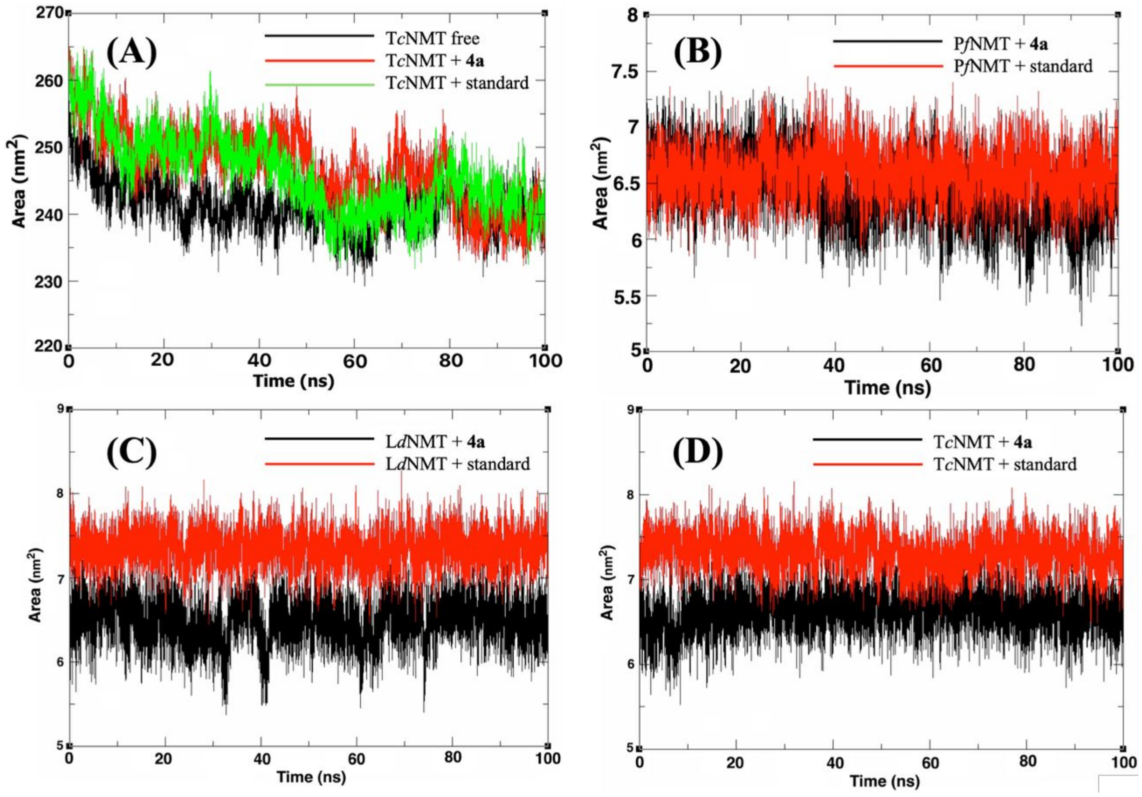

Fig. S119. Molecular dynamics simulation results of SASA of the protein for the: **(A)** T*c*NMT free (black line), and in complex with **4a** (red line) and standard compound (green line); **(B)** SASA of the ligands **4a** (black line) and standard compound (red line) complexed P*f*NMT; **(C)** SASA of the ligands **4a** (black line) and standard compound (red line) complexed with L*d*NMT; and **(D)** SASA of the ligands **4a** (black line) and standard compound (red line) in complex with T*c*NMT.
